# Pd-catalyzed C–C and C–N cross-coupling reactions in 2-aminothieno[3,2-*d*]pyrimidin-4(3*H*)-one series for antiplasmodial pharmacomodulation†

**DOI:** 10.1039/d2ra01687g

**Published:** 2022-07-08

**Authors:** Romain Mustière, Prisca Lagardère, Sébastien Hutter, Céline Deraeve, Florian Schwalen, Dyhia Amrane, Nicolas Masurier, Nadine Azas, Vincent Lisowski, Pierre Verhaeghe, Dominique Mazier, Patrice Vanelle, Nicolas Primas

**Affiliations:** Aix Marseille Université, CNRS, ICR UMR 7273, Equipe Pharmaco-Chimie Radicalaire, Faculté de Pharmacie Marseille France nicolas.primas@univ-amu.fr; Institut des Biomolécules Max Mousseron, UMR 5247, CNRS, Université de Montpellier, ENSCM, UFR des Sciences Pharmaceutiques et Biologiques Montpellier France; Aix Marseille Université, IRD, AP-HM, SSA, VITROME Marseille France; LCC-CNRS, Université de Toulouse, CNRS UPR 8241, UPS Toulouse France; CHU de Nîmes, service de pharmacie Nimes France; Centre d’Immunologie et des Maladies Infectieuses (CIMI), INSERM, CNRS, Sorbonne Université Paris France; Service Central de la Qualité et de l'Information Pharmaceutiques, AP-HM, Hôpital Conception Marseille France

## Abstract

In 2015, we identified gamhepathiopine (M1), a 2-*tert*-butylaminothieno[3,2-*d*]pyrimidin-4(3*H*)-one antiplasmodial hit targeting all development stages of the human malarial parasite *P. falciparum*. However, this hit compound suffers from sensitivity to hepatic oxidative metabolism. Herein, we describe the synthesis of 33 new compounds in the 2-aminothieno[3,2-*d*]pyrimidin-4(3*H*)-one series modulated at position 6 of this scaffold. The modulations were performed using three palladium-catalyzed cross coupling reactions, namely Suzuki–Miyaura, Sonogashira, and Buchwald–Hartwig. For the latter, we developed the reaction conditions. Then, we evaluated the synthesized compounds for their antiplasmodial activity on the K_1_*P. falciparum* strain and their cytotoxicity on the human HepG2 cell line. Although we did not obtain a compound better than M1 in terms of the antiplasmodial activity, we identified compound 1g bearing a piperidine at position 6 of the thieno[3,2-*d*]pyrimidin-4(3*H*)-one ring with an improved cytotoxicity and metabolic stability. 1g is an interesting new starting point for further pharmacomodulation studies. This study also provides valuable antiplasmodial SAR data regarding the nature of the ring at position 6, the possible substituent on this ring, and the introduction of a spacer between this ring and the thienopyrimidinone moiety.

## Abstract 2

Pharmacomodulation at position 6 of a thienopyrimidinone antiplasmodial hit using palladium-catalyzed cross-coupling reactions afforded 33 new compounds, among which a new hit was found with enhanced metabolic stability.

## Graphical abstract

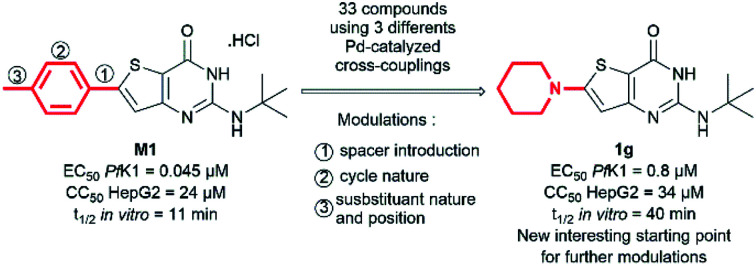

## 1. Introduction

Malaria is a heavy burden for endemic countries, mainly in Africa, with an estimated 241 million cases and 627 000 deaths in 2020, according to the World Health Organization.^1^ Seventy seven percent of the victims were children under 5 years old. The disease is caused by parasites of the *Plasmodium* genus transmitted by the bite of an infected female mosquito belonging to the *Anopheles* genus. Of the five species causing malaria in humans, *P. falciparum* is responsible for the most deaths. Along with the spread of long-lasting insecticide-treated bed nets, the introduction of artemisinin-based combined therapies (ACT) as first line treatments against severe *P. falciparum* infections were key to the fight against malaria.^2^

**Fig. 1:**
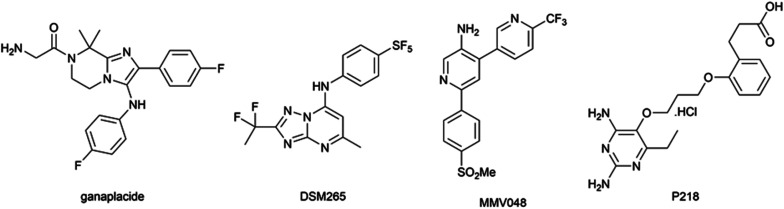
Chemical structures of antimalarial compounds in clinical trial or recently withdrawn from them.

However, the emergence of artemisinin-resistant *P. falciparum* in South-East Asia first reported in 2009,^3^ mediated by *Pf*kelch13 mutations,^4^ and its spread in the greater Mekong subregion is currently limiting the clinical effectiveness of ACT in this area.^5^ While the combination of ganaplacide (Fig. 1) and lumefantrine is the most promising drug combination currently being investigated by Medicines for Malaria Venture (MMV),^6^ the recent withdrawal of MMV048, P218, and DSM265 in clinical trials (for teratogenicity, low half-time *in vivo*, and high selection of resistances, respectively; Fig. 1)^7,8^ increases the urgent need for new antimalarial compounds with novel mechanisms of action.

**Scheme 1:**
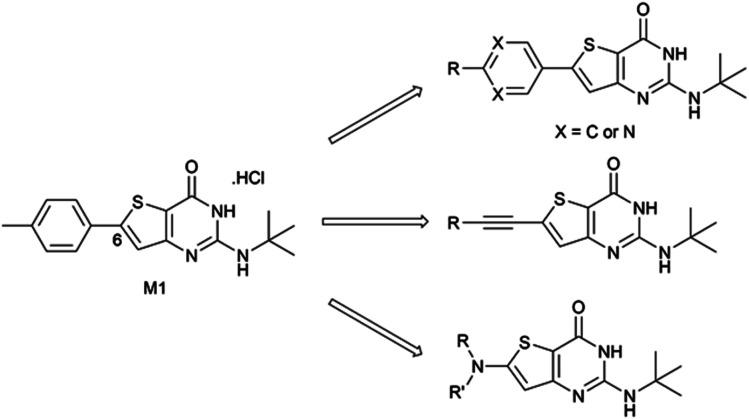
Chemical structure of compound M1 and general structures for the compounds presented in this work.

In 2015, we described the synthesis and activity of a new molecule called gamhepathiopine (also M1, Scheme 1) that is active on all stages of *P. falciparum*, including quiescent parasites of the artemisinin-resistant strain F32-ART5, with an unknown mechanism of action.^9^ We later discovered that the *in vivo* activity of M1 was limited by its sensitivity to mouse hepatic metabolism. Indeed, the *in vivo* activity in *P. berghei* infected mice was only achieved with the addition of 1-aminobenzotriazole, a pan-CYP450 inhibitor.^10^ Therefore, the synthesis of new thieno[3,2-*d*]pyrimidines to afford a new hit compound with improved metabolic stability and similar activity profile was initiated. We also expected to obtain new antiplasmodial SAR data on the thieno[3,2-*d*]pyrimidine series. Indeed, we observed important modifications to the biological values with minor modifications on the molecular scaffold during the first round of pharmacomodulations, leading to compound M1.^9^ Previously explored substituents on the phenyl ring at position 6 of the thienopyrimidinone scaffold were simple, *i.e.*, halogen atoms or methoxy group, but some of them maintained good antiplasmodial potencies. Moreover, on M1, position 6 (and position 2) is sensitive to oxidative metabolism because of the methyl group.^10^ Thus, we decided to explore more diverse substituents at this position with the help of palladium-catalyzed cross-coupling reactions. Herein, we describe the synthesis of 2-aminothieno[3,2-*d*]pyrimidin4(3*H*)-ones, structurally modulated on the west part of the scaffold at position 6 (Scheme 1) by Buchwald–Hartwig, Sonogashira, or Suzuki–Miyaura cross-coupling reactions, along with their antiplasmodial and cytotoxicity evaluations.

## 2. Results and discussion

### 2.1. Buchwald–Hartwig coupling

To our knowledge, no examples of Buchwald–Hartwig coupling on a thienopyrimidine cycle are reported in the literature to date and there are only a few examples of this coupling on a thiophene ring.^11–13^ Therefore, we first explored the reaction conditions between 6-bromo-2-*tert*butylaminothieno[3,2-*d*]pyrimidin-4(3*H*)-one 1 and *para*-toluidine in order to determine the best reaction conditions (Table 1). During different experiments, we used LC-MS to estimate the yield of product 1a.

**Table 1:** Optimization of the Buchwald–Hartwig cross-coupling reaction between 1 and p-toluidine

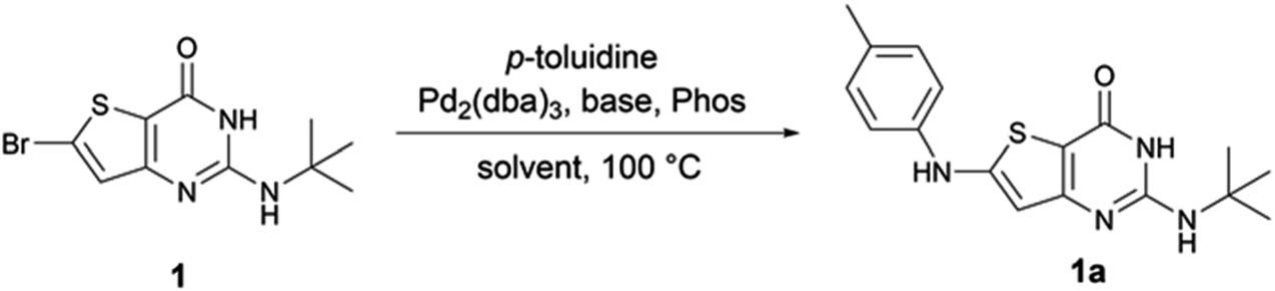								
Entry	Eq. *p*-toluidine	mol% Pd	Phosphine	Mol% Phos	Base	Eq. Base	Solvent	Estimated yield^a^ of 1a (%)
1	1.2	2	Rac-BINAP	4	Cs_2_CO_3_	3	Dioxane	0
2	1.2	10^b^	Rac-BINAP	10	Cs_2_CO_3_	1.4	Toluene	0
3	1.2	10^b^	Rac-BINAP	10	NaO*t*Bu	1.4	Toluene	0^c^
4	1.2	2	RuPhos	8	NaO*t*Bu	1.4	Toluene	0
5	1.2	5	RuPhos	10	NaO*t*Bu	3	Toluene	0
6	1.2	5	SPhos	10	NaO*t*Bu	3	Toluene	Traces^c^
7	1.2	5	RuPhos	10	NaO*t*Bu	3	Dioxane	4
8	1.2	5	SPhos	10	NaO*t*Bu	3	Dioxane	46
9	1.2	5	XPhos	10	NaO*t*Bu	3	Dioxane	47
10	1.2	5	*t*BuXPhos	10	NaO*t*Bu	3	Dioxane	0
11	1.2	5	SPhos	10	LiHMDS	3	Dioxane	0
12	1.2	5	SPhos	10	NaO*t*Bu	3	DME	Traces^c^
13	5	5	SPhos	10	NaO*t*Bu	3	Dioxane	66
14	5	5	XPhos	10	NaO*t*Bu	3	Dioxane	100 (1 h)
15	3	5	XPhos	10	NaO*t*Bu	3	Dioxane	100 (2 h)
16	5	4	XPhos	8	NaO*t*Bu	3	Dioxane	100 (2 h)

a Using LCMS.

b Pd(dba)_2_.

c Dehalogenation of the starting material mainly observed.

First, we used the conditions described in the literature involving a thiophene ring (entry 1,^11^ entries 2–3,^12^ and entries 4–5,^13^Table 1). We did not observe any reaction with these conditions. From the conditions of entry 5, we then decided to study the influence of the phosphine ligand using SPhos instead of RuPhos (entry 6, Table 1). While we observed 1a for the first time, the main product of the reaction resulted from the dehalogenation of the starting material. Also, from entry 5, we decided to improve the solubility of starting material 1 by changing the reaction solvent from toluene to dioxane (entry 6, Table 1). We observed the formation of 1a in an estimated 4% yield. By combining the conditions of entries 6 and 7, the estimated yield increased to 46% (entry 8, Table 1). Using the closely related XPhos phosphine, instead of SPhos, the same estimated yield was maintained (entry 9, Table 1). However, *t*BuXPhos, a more hindered version of XPhos, did not allow the formation of 1a. From entry 8, we tried to explore the changes in the base and the solvent using LiHMDS instead of NaO*t*Bu (entry 11, Table 1) and using DME instead of dioxane (entry 12, Table 1). The use of LiHMDS was unsuccessful in affording 1a, while DME caused a significant dehalogenation of 1. From entries 8 and 9, we decided to increase the load of *p*-toluidine in the reaction (entries 13 and 14, Table 1). We observed an increase in the estimated yield with full conversion in 1 h when XPhos was used. From these conditions, we reduced the load of *p*-toluidine or the phosphine/palladium duo (entries 15 and 16, Table 1), and we observed only an increase in the reaction time from 1 to 2 h. We finally decided to use the conditions of entry 16 thereafter since amines are cheap starting materials compared to palladium and phosphine.

The Ullman reaction, a possible alternative to the Buchwald–Hartwig coupling reaction, was also tried on compound 1 or its precursor 2. In both cases, no reaction was observed after 24 h at 100 °C with the following tested conditions: *para*-toluidine (3 eq.), dimethylglycine (20 mol%), CuI (10 mol%), and Cs_2_CO_3_ (3 eq.).

Therefore, the conditions of entry 16 (Table 1) were then applied on various substrates to explore the scope of the operating procedure and obtain a new series of functionalized 2-*tert*butylaminothieno[3,2-*d*]pyrimidin-4(3*H*)-one (Table 2).

**Table 2:** Scope of the Buchwald–Hartwig cross-coupling reaction on compound 1

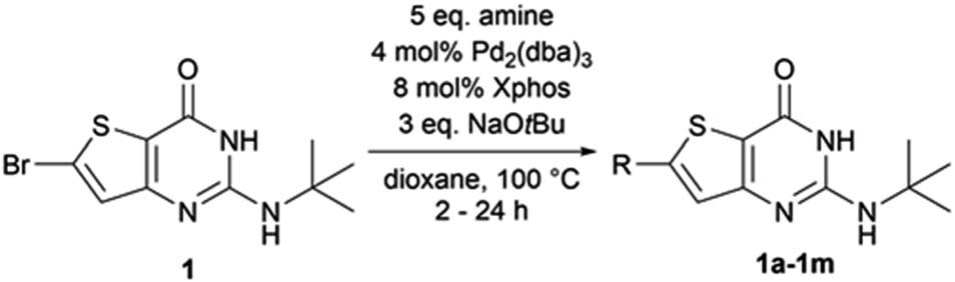		
Compound	R	Isolated yield^a^ (%) or observed outcome
1a	4-Me–Ph–NH–	55
1b	3-Me–Ph–NH–	31
1c	2-Me–Ph–NH–	15 (53)
1d	4-MeO–Ph–NH–	14 (63)
1e	4 F–Ph–NH–	23 (65)
1f	Morpholinyl–	35 (74)
1g	Piperidinyl–	15 (51)
1h	*N*-Methylpiperazinyl–	Complete dehalogenation of 1
1i	Pyrazole	No reaction
1j	Imidazole	No reaction
1k	Pyrimidinyl-3–amine	Traces of 1k and dehalogenated byproduct
1l	Cyclopropylamine	Complete dehalogenation of 1
1m	Ethanolamine	Complete dehalogenation of 1

a Conversion rate (determined by LCMS) indicated between brackets if the conversion was not complete.

Purifications, using flash chromatography, were challenging for the whole series, thus leading to a moderate isolated yield of 55% for compound 1a. The isolated yields for the other compounds were decreased by two additional factors–incomplete conversion and dehalogenation of 1. When moving the methyl substituent from the *para* position to the *ortho* (1c) position, the conversion was not complete after 24 h. Conversion was also incomplete after 24 h of heating when the methyl group was replaced by an electron-donating (1d) or electron-withdrawing (1e) group. When performing the reaction with morpholine (1f) or piperidine (1g), we also observed moderate dehalogenation of the starting material in addition to incomplete conversion after 24 h, both reasons being responsible for the low reaction yields. For *N*-methylpiperazine (1h), we observed a complete and rapid (less than 2 h) dehalogenation of the starting material. The same result was observed with alkyl amines such as cyclopropylamine (1l) and ethanolamine (1m). This dehalogenation could be caused by the choice of our catalyst ligand.^14^ Finally, heteroaromatic amines, such as pyrazole, imidazole, and 3-aminopyridine, did not react using the same conditions. Although our conditions seemed to work for a simple substrate such as *para*-toluidine, they are strongly impacted by the modification of the *para*-substituent and the nature of the amine used.

We applied these Buchwald–Hartwig conditions to compound 2, the synthetic precursor of compound 1, to possibly overcome the dehalogenation problem encountered with aliphatic amines and *N*-methylpiperazine (Table 3). While our conditions properly functionalized position 5 of the thiophene aminoester, we also observed a partial saponification of the methyl ester group. The corresponding carboxylic acid derivative could not be isolated because of its high polarity. We hypothesized that sodium *tert*butoxyde could saponify the ester at position 2 of thiophene. We decided to explore other bases to observe the behavior of the ester; caesium carbonate promoted the homo-coupling of compound 2, while LiHMDS led to the formation of a cyclopropylamide derivative. The replacement of the phosphine ligand with caesium carbonate did not cause any change and the homo-coupling product was still formed.

**Table 3:** Optimization attempts for Buchwald–Hartwig cross-coupling reaction on compound 2

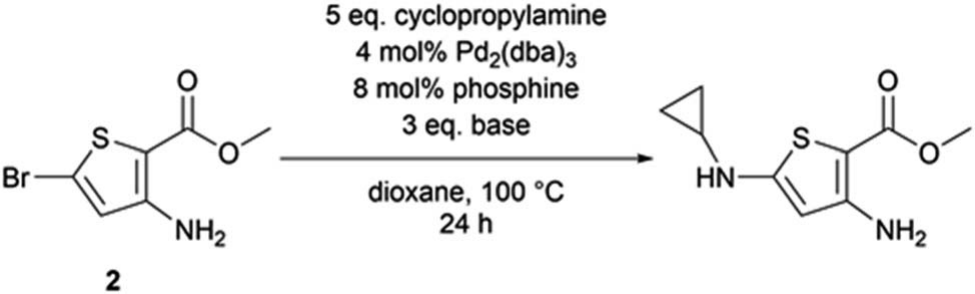			
Entry	Base	Phosphine	Putative outcome^a^
1	*t*BuONa	XPhos	Cyclopropylamine introduced at position 5 – saponification of the methyl ester
2	Cs_2_CO_3_	XPhos	Homocoupling of 2
3	LiHMDS	XPhos	Conversion of the methyl ester into cyclopropylamide
4	Cs_2_CO_3_	BrettPhos	Homocoupling of 2

a from LCMS observations.

### 2.2. Sonogashira coupling

**Scheme 2:**
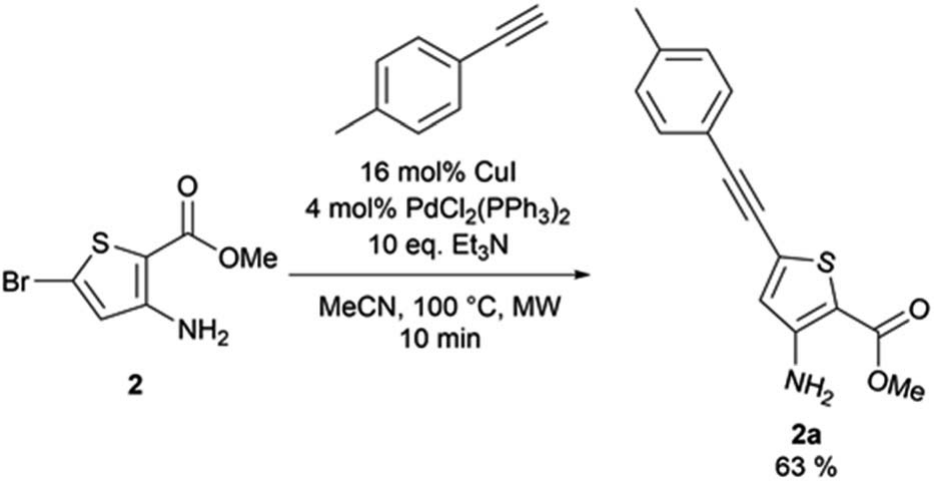
Sonogashira reaction on compound 2.

From the reaction conditions found in the literature,^15^ we performed a conclusive test on compound 2 (Scheme 2). We then used these reaction conditions on compound 1 and its isopropylamine analog 3, leading to seven 2-aminothieno[3,2-*d*]pyrimidin-4(3*H*)-ones bearing an alkyne substituent at position 6 in yields ranging from 23 to 85% (Table 4). The poor yields are due either to purification difficulties (compound 2c and 2g) or the volatile behavior of some alkynes (compound 2e). Depending on the boiling point of the alkyne, we tried to improve the low yields by increasing the load of the alkyne in the reaction, but still observed low yields. Compound 2f was obtained after the protodesilylation of the trimethylsilyl alkyne using potassium carbonate in a mixture of methanol and dichloromethane.

**Table 4:** Synthesis of 6-alkynyl-2-aminothieno[3,2-d]pyrimidin4(3H)-ones 2b–2h

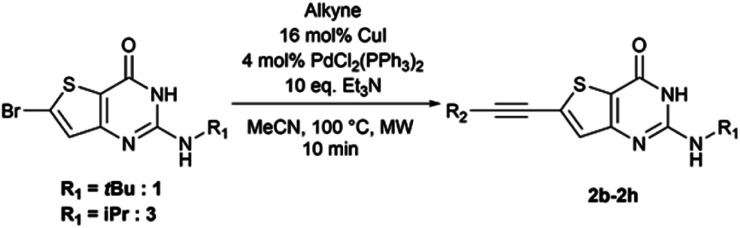			
Compound	R_1_	R_2_	Yield (%)
2b	*t*Bu	4-Me–Ph–	60
2c	*i*Pr	4-Me–Ph–	23
2d	*t*Bu	HO(CH_2_)_2_–	47
2e	*t*Bu	Cyclopropyl–	23
2f	*t*Bu	H–	57^a^
2g	*t*Bu	*N*-Methylpiperazinyl–CH_2_–	35
2h	*i*Pr	Ph–	85

a Two steps.

### 2.3. Suzuki–Miyaura coupling

**Scheme 3:**
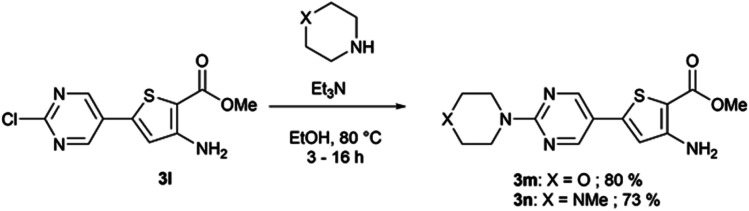
S_N_Ar reaction on compound 3l.

When Suzuki–Miyaura coupling was performed on compound 1 or 3, applying the reaction conditions used for the synthesis of M1 metabolites,^10^ we observed the dehalogenation of the starting material, which complicated the purification. Therefore, we decided to perform Suzuki–Miyaura coupling on 2 with conditions from the literature and previously used in our lab.^16^ Eleven thiophene aminoesters functionalized at position 5 were synthesized in yields above 50%, except for compound 3l (Table 5). In this specific case, we removed water from the reaction medium to avoid the substitution of the chlorine atom contained in the chloroimine moiety. The chlorine atom of compound 3l was then engaged in a S_N_Ar reaction with morpholine or *N*-methylpiperazine (Scheme 3).

**Table 5:** Synthesis of 5-functionalized thiophene aminoesters 3a–3l

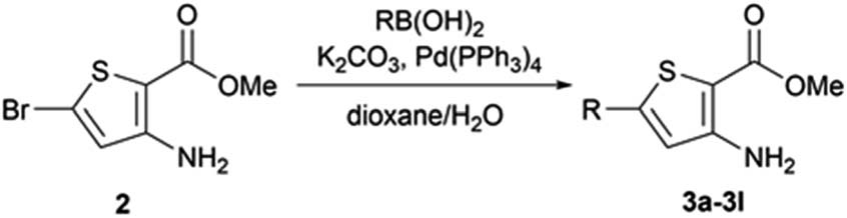		
Compound	R	Isolated yield (%)
3a	4-CO_2_Me–Ph–	55
3b	4-NO_2_–Ph–	65
3c	4-HO–Ph–	64
3d	3-Cl-4-F–Ph–	82
3e	4-Morpholinyl–Ph–	73
3f	4-(*N*-Methylpiperazinyl)–Ph–	71
3g	4-Piperidinyl–Ph–	71
3h	4-MeS–Ph–	72
3i	4-MeSO_2_–Ph–	62
3j	4-(H_2_N–SO_2_)–Ph–	47
3k	4-(Morpholinyl–SO_2_)–Ph–	88
3l	2-Chloropyrimidin-5-yl–	44

**Scheme 4:**
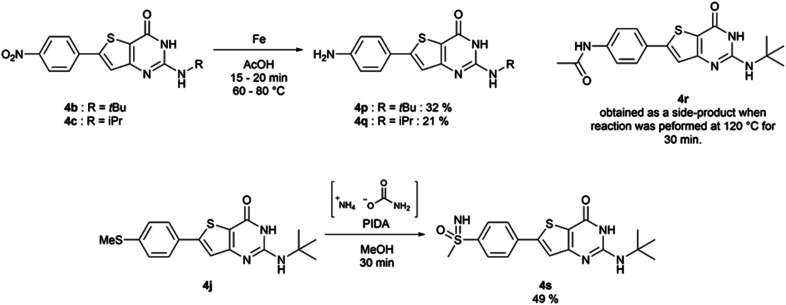
Synthesis of compounds 4p–4s.

The functionalized thiophene aminoesters (3a–3k, 3m, and 3n) were then engaged in a cyclization reaction to form the thieno[3,2-*d*]pyrimidin-4(3*H*)-one core. This three step one-pot reaction afforded 15 compounds, 13 including a *tert*butylamine group and 2 bearing an isopropyl group at position 2 (Table 6). The 4-nitrophenyl-containing compounds 4b and 4c were then engaged in a reduction reaction to afford the 4-aminophenyl compounds 4p and 4q in poor yields (Scheme 4). We initially performed this reduction at 120 °C for 30 min, but this led to the formation of compound 4r, resulting from the acylation of compound 4p, which was isolated. Compound 4j was used to synthesize the sulfoximine derivative 4s (Scheme 4) using a one-step reaction with ammonium carbamate and (diacetoxyiodo)benzene (PIDA).

**Table 6:** Cyclization reaction leading to compounds 4a–4o

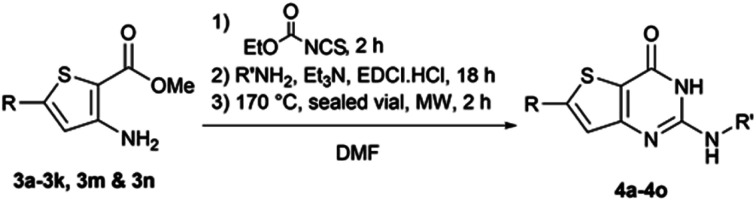			
Compound	R	R′	Isolated yield (%)
4a	4-CO_2_Me–Ph–	*t*Bu	55
4b	4-NO_2_–Ph–	*t*Bu	55
4c		iPr	33
4d	4-HO–Ph–	*t*Bu	13
4e	3-Cl-4-F–Ph–	*t*Bu	49
4f		iPr	37
4g	4-Morpholinyl–Ph–	*t*Bu	69
4h	4-(*N*-Methylpiperazinyl)–Ph–	*t*Bu	23
4i	4-Piperidinyl–Ph–	*t*Bu	51
4j	4-MeS–Ph–	*t*Bu	65
4k	4-MeSO_2_–Ph–	*t*Bu	44
4l	4-(H_2_N–SO_2_)-Ph-	*t*Bu	56
4m	4-(Morpholinyl–SO_2_)–Ph–	*t*Bu	33
4n	2-Morpholinopyrimidin-5-yl–	*t*Bu	57
4o	2-(*N*-Methylpyperazin-1-yl)pyrimidin-5-yl–	*t*Bu	19

### 2.4. Biological evaluation

The synthesized compounds were evaluated for their antiplasmodial activity (EC_50_) on the multi-resistant K_1_ strain of *P. falciparum* (resistant to chloroquine, sulfadoxine, and pyrimethamine) and their cytotoxicity (CC_50_) on the human HepG2 cell-line in order to determine their selectivity index (SI, EC_50_/CC_50_) and to compare them to reference drugs (chloroquine, atovaquone, and doxycycline for activity and doxorubicine for cytotoxicity; Table 7). Of the 33 tested compounds, 16 displayed aqueous solubility problems in the test medium, limiting the determination of precise values for EC_50_, CC_50_, or both. Such behavior for the thieno[3,2-*d*]pyrimidin-4(3*H*)-one series was already seen during the pharmacomodulation, leading to compound M1.^9^ The thienopyrimidinone scaffold is found in cytotoxic compounds,^17^ but apart from compound 4r (CC_50_ = 0.16 μM, more toxic than doxorubicine), the CC_50_ of the compounds ranged widely from 2.1 to 89.1 μM, while the EC_50_ ranged from 0.77 to 43.2; 18 compounds showed an antiplasmodial activity below 5 μM. Compound 1g was the best among all the synthesized compounds with submicromolar antiplasmodial activity (0.77 μM), which is still far from the M1 potency (0.045 μM) but with improved cytotoxicity (33.9 μM) compared to M1 (24 μM).^9^ These results provided valuable data regarding the SAR of the thieno[3,2-*d*]pyrimidin-4(3*H*)-one core.

**Table 7:** Antiplasmodial activity and cytotoxicity of the synthesized 2-aminothieno[3,2-*d*]pyrimidin4(3*H*)-ones

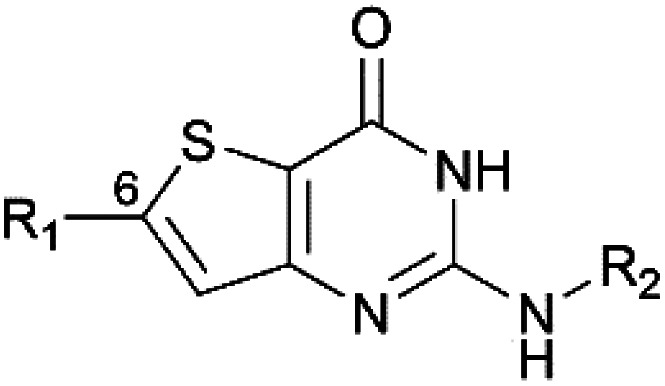						
Entry	R_1_	R_2_	EC_50_*Pf*K_1_ (μM)	CC_50_ HepG2 (μM)	SI	*c* log *D*_7.4_^b^
1	Br	*t*Bu	43.2 ± 2.6	38.2 ± 3.5	0.9	3.19
3		iPr	>50	89.1 ± 9.7	<1.8	2.91
1a	4-Me–Ph–NH–	*t*Bu	10.3 ± 0.2	21.7 ± 2.1	2.1	4.38
1b	3-Me–Ph–NH–	*t*Bu	1.1 ± 0.3	5.3 ± 0.4	4.8	4.38
1c	2-Me–Ph–NH–	*t*Bu	4.3 ± 0.2	14.1 ± 0.3	3.3	4.38
1d	4-MeO–Ph–NH–	*t*Bu	7.2 ± 2.0	24.0 ± 3.1	3.3	3.71
1e	4 F–Ph–NH–	*t*Bu	7.1 ± 1.4	21.3 ± 1.6	3.0	4.01
1f	Morpholinyl–	*t*Bu	4.5 ± 1.1	>50	>11.1	2.31
1g	**Piperidinyl–**	***t*Bu**	**0.77 ± 0.13**	**33.9 ± 1.4**	**44.0**	**3.38**
2b	4-Me–Ph–	*t*Bu	2.35 ± 0.38	>12.5	>5.3	5.03
2c		iPr	4.6 ± 1.0	3.1 ± 0.6	0.7	4.75
2d	HO(CH_2_)_2_–	*t*Bu	>10	>50	—	2.39
2e	Cyclopropyl–	*t*Bu	1.9 ± 0.6	20.8 ± 1.4	10.9	3.65
2f	H–	*t*Bu	>12.5	4.7 ± 1.0	<0.4	2.54
2g	*N*-Methylpiperazine–CH_2_–	*t*Bu	>5	41.6 ± 5.2	<8.3	2.20
2h	Ph–	iPr	2.8 ± 1.0	>12.5	>4.5	4.23
4a	4-CO_2_Me–Ph–	*t*Bu	2.5 ± 0.9	>12.5	>5.0	3.91
4d	4-HO–Ph–	*t*Bu	7.5 ± 1.0	12.2 ± 0.3	1.6	3.60
4e	3-Cl-4-F–Ph–	*t*Bu	2.0 ± 0.9	>12.5	>6.3	4.65
4f		iPr	3.6 ± 1.2	>6.25	>1.7	4.37
4g	4-Morpholinyl–Ph–	*t*Bu	2.4 ± 0.9	7.9 ± 1.3	3.3	3.79
4h	4-(*N*-Methylpiperazinyl)–Ph–	*t*Bu	4.1 ± 0.7	5.8 ± 0.5	1.4	3.29
4i	4-Piperidinyl–Ph-	*t*Bu	4.1 ± 1.2	>6.25	>1.5	4.86
4j	4-MeS–Ph–	*t*Bu	1.0 ± 0.3	>12.5	>12.5	4.53
4k	4-MeSO_2_–Ph–	*t*Bu	3.0 ± 0.6	>25	>8.3	2.74
4l	4-(H_2_N–SO_2_)–Ph–	*t*Bu	>12.5	>6.25	—	2.51
4m	4-(Morpholinyl–SO_2_)–Ph–	*t*Bu	5.1 ± 2.6	>12.5	>2.5	2.74
4n	2-Morpholinopyrimidin-5-yl–	*t*Bu	2.1 ± 0.6	26.4 ± 4.6	12.6	2.55
4o	2-(*N*-Methylpyperazin-1-yl)pyrimidin-5-yl–	*t*Bu	4.4 ± 1.0	11.2 ± 1.3	2.5	2.40
4p	4-NH_2_-Ph–	*t*Bu	3.5 ± 1.2	2.5 ± 1.0	0.7	3.07
4q		iPr	6.0 ± 1.1	11.3 ± 3.5	1.9	2.79
4r	4-(CH_3_–CO–NH)-Ph–	*t*Bu	3.9 ± 1.1	0.16 ± 0.04	<0.1	3.14
4s	4-(CH_3_–SONH)-Ph–	*t*Bu	>12.5	63.3 ± 14.6	<5.1	2.76
	**M1** a		**0.045**	**24**	**533**	**4.42**
	**M1 (free base)** a		**0.2**	**25.6**	**128**	**4.42**
	**Chloroquine** a		**0.5**	**30**	**60**	**0.88**
	**Atovaquone** a		**0.0013**	**>15.6**	**>12 000**	**4.37**
	**Doxycycline** a		**5**	**20**	**4**	**−0.80**
	**Doxorubicine** a		—	0.2	—	**0.04**

a Values from ref. 9.

b See experimental part for details.

The introduction of an amine spacer between the thienopyrimidinone core and the C6-substituent (1a) significantly reduced the antiplasmodial activity. The cytotoxicity value was worsened when the methyl was moved on the phenyl ring (*para*1a < *ortho*1c < *meta*1b). The replacement of the methyl group by an electron-donating (1d) or withdrawing (1e) group moderately improved the antiplasmodial activity. The introduction of an alkyne spacer in the M1 structure (2b) reduced the antiplasmodial activity and aqueous solubility in the test medium of the compound. Among the different alkyne substituents, only cyclopropylalkyne 2e showed a decent combination of the biological values. Therefore, the conformational changes of the C6-sidechain induced by the amine spacer or sidechain elongation following the introduction of an alkyne are not effective strategies to obtain an antiplasmodial hit compound in the thienopyrimidinone series.

We also investigated the *para*-substituent on the phenyl ring at position 6 of the thienopyrimidinone core. The replacement of the methyl substituent by a more polar group to potentially improve the aqueous solubility (methyl ester 4a, phenol 4d, anilines 4p and 4q and acetamide 4r, and sulphur-containing functions 4j–4m and 4s) was favorable for neither the antiplasmodial activity nor the cytotoxicity, while the replacement of the methyl by a fluorine atom was interesting in our previous work;^9^ the addition of a chlorine atom at position 3 of the 4-fluorophenyl cycle induced a loss of activity and aqueous solubility in the test medium (4e and 4f) compared to M1. We also replaced the methyl substituent by different saturated heterocycles (4g, 4h and 4i), but this was associated with an increased cytotoxicity and reduced antiplasmodial activity. The cytotoxicity values were improved when the phenyl ring was replaced by a pyrimidine (4n and 4o).

Finally, Buchwald–Hartwig cross-couplings gave us the opportunity to replace the phenyl ring at position 6 by saturated heterocycles. When the phenyl ring is replaced by a piperidine (1g), we observed a moderate loss of activity and improved cytotoxicity. The addition of an oxygen atom in the cyclic amine, leading to morpholine 1f, significantly decreased the antiplasmodial activity.

**Fig. 2:**
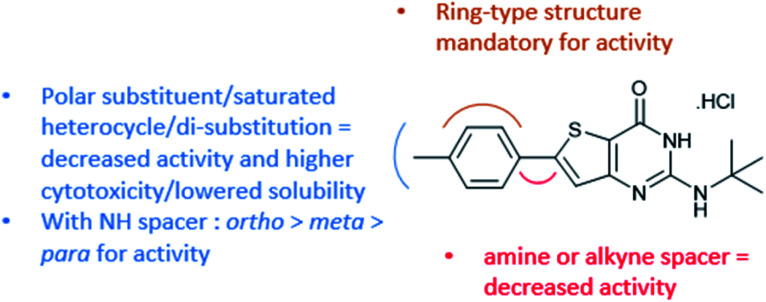
RSA data obtained from the biological results.

With this observation and the biological results of compounds bearing a polar substituent (4a, 4d, 4g, 4h, and 4k–4s), we concluded that a polar head (linked to *c* log *D*_7.4_ decrease) at this position is detrimental to the antiplasmodial activity (Fig. 2). We also concluded that a ring-type structure (aromatic or saturated) is required at position 6 since compounds lacking one of these structures (1, 3, 2d, and 2f) displayed a lack of antiplasmodial activity.

Considering 1g gave good biological results, it was evaluated *in vitro* for its mouse microsomal stability. We observed a more than 3-fold improvement in the half-time between the tolyl moiety (M_1_, *t*_1/2_ = 11 min, Cl_int_ = 240 μLmin^−1^mg) and the piperidine moiety (1g, *t*_1/2_ = 40 min, Cl_int_ = 42 μLmin^−1^mg). This important improvement shows that the piperidine moiety is stable.

## 3. Conclusion

We synthesized 33 original compounds in the thieno[3,2-*d*]pyrimidin-4(3*H*)-one series using three different palladium-catalyzed cross-coupling reactions, namely Buchwald–Hartwig, Sonogashira, and Suzuki–Miyaura. For the first time, we developed reaction conditions to directly functionalize the position 6 of the thienopyrimidinone core with anilines and saturated heterocycles. However, additional work on this reaction is required to extend the scope of useable substrates and to improve the reaction yields. Sonogashira coupling provided compounds with the side chains of various natures. However, the introduction of the alkyne spacer that reduced the antiplasmodial activity led us to stop the further synthesis of 6-alkynyl-thieno[3,2-*d*]pyrimidin-4(3*H*)-ones. Seventeen compounds were obtained using Suzuki–Miyaura coupling with various substituents on the phenyl ring, and there are still a lot of possibilities to explore with this coupling. The synthesized compounds were tested on the blood-stage of *Pf*K_1_ and the human HepG2 cell-line to measure their antiplasmodial activity and cytotoxicity. This work led us to the discovery of compound 1g, which has submicromolar antiplasmodial activity that is associated with a better cytotoxicity value than compound M1 and an improved *in vitro* metabolic stability compared to M1. Moreover, this work provided valuable RSA data on the thienopyrimidinone series (Fig. 2). Considering the microsomal stability of the piperidine moiety, 1g is an interesting starting point to synthesize new thieno[3,2-*d*]pyrimidines bearing, at position 6, a substituted piperidine. The substitution on this piperidine could provide us compounds with biological values at par with the M1 antiplasmodial profile and further improve the metabolic stability of the compounds.

## 4. Experimental part

### 4.1. Chemistry

#### 4.1.1. Materials and methods

Starting materials were purchased from Sigma-Aldrich (Saint Louis, MO, USA) or Fluorochem (Derbyshire, UK). NMR spectra were recorded on a Bruker Avance 250 MHz or a Bruker Avance NEO 400 MHz NanoBay spectrometer at the “Faculté de Pharmacie” of Marseille. The residual proton signal of the deuterated solvent was used as an internal reference: CDCl_3_*δ* = 7.26 ppm for ^1^H and 77.16 for ^13^C, and DMSO-*d*_6_*δ* = 2.50 ppm for ^1^H and 39.52 ppm for ^13^C. Data for ^1^H NMR are reported as follows: chemical shifts (*δ*) in parts per million (ppm), multiplicity (described as follows: s, singlet; bs, broad singlet; d, doublet; t, triplet; q, quadruplet; dd, doublet of doublet; ddd, doublet of doublet of doublet; m, multiplet), coupling constants (*J*) in Hertz (Hz), and integration. Data for ^13^C NMR are reported as follows: chemical shifts (*δ*) in parts per million (ppm). Melting points were determined on a Köfler melting point apparatus (Wagner & MunzGmbH, München, Germany) and are uncorrected. HRMS spectra (ESI) were recorded on a SYNAPT G2 HDMS (Waters) and were performed at the “Faculté des Sciences” of Marseille (St Jérôme campus). Silica Gel 60 (Merk 70–230) was used for column chromatography. Flash chromatography was performed on a puriFlash® 5.020 apparatus (Interchim, Montluçon, France). TLC was performed on aluminium plates coated with silica gel 60F-254 (Merck) using an appropriate eluent. Visualization was carried out with ultraviolet light (254 or 365 nm). Reactions using microwave heating were performed with a Biotage® Initiator or Initiator + apparatus (Biotage, Uppsala, Sweden).

#### 4.1.2. Procedure for the screening of Buchwald–Hartwig coupling conditions

6-Bromo-2-(*tert*-butylamino)thieno[3,2-*d*]pyrimidin-4(3*H*)-one (50 mg, 0.17 mmol), 4-methylaniline, tris(dibenzylideneacetone)dipalladium(0), appropriate phosphine ligand, and appropriate base were put under nitrogen atmosphere and an appropriate dried solvent (0.01 M) was added. The obtained suspension was stirred at 100 °C and the reaction's progress was followed using LC-MS (typically 1, 2, 18, and 48 h after the beginning of the reaction).

#### 4.1.3. General procedure A for the synthesis of compounds through Buchwald–Hartwig coupling

In a sealed microwave vial, 6-bromo-2-(*tert*-butylamino)thieno[3,2-*d*]pyrimidin-4(3*H*)-one (0.3 g, 1 mmol), appropriate amine (5 mmol), tris(dibenzylideneacetone)dipalladium(0) (36 mg, 4% mol), XPhos (38 mg, 8% mol), and sodium *tert*-butoxyde (0.286 g, 3 mmol) were put under nitrogen atmosphere and dried dioxane (0.01 M) was added. The obtained suspension was stirred at 100 °C up to 24 h (reaction progress followed by LC-MS). Excess solvent was removed *in vacuo* and the obtained crude was dissolved in ethyl acetate (40 mL). The organic phase was washed with brine (2 × 40 mL), dried over sodium sulphate, and the excess solvent was removed *in vacuo*. The obtained crude was purified using an appropriate method.

##### 2-*tert*-Butylamino-6-[(4-methylphenyl)amino]thieno[3,2-*d*]pyrimidin-4(3*H*)-one (1a)

Following procedure A with 4-methylaniline (0.532 g, 5 mmol), the obtained crude was purified *via* flash chromatography (using dichloromethane/methanol). Fractions of interest were triturated in diethyl ether, affording 2-*tert*-butylamino-6-[(4-methylphenyl)amino]thieno[3,2-d]pyrimidin-4(3*H*)-one as a brown powder (173 mg, 55% yield). ^1^H NMR (DMSO-*d*_6_, 400 MHz) *δ* 10.06 (s, 1H), 9.47 (s, 1H), 7.17–7.07 (m, 4H), 6.22 (s, 1H), 5.94 (s, 1H), 2.25 (s, 3H), 1.38 (s, 9H). ^13^C NMR (DMSO-*d*_6_, 100 MHz) *δ* 160.5, 156.8, 155.4, 152.3, 139.4, 130.7, 129.8 (2C), 117.5 (2C), 101.5, 98.5, 50.8, 28.7 (3C), 20.3. Mp = 204–206 °C. HRMS (ESI) *m*/*z* calculated for C_17_H_21_N_4_OS [M + H]^+^ 329.1431, found 329.1429.

##### 2-*tert*-Butylamino-6-[(3-methylphenyl)amino]thieno[3,2-d]pyrimidin-4(3*H*)-one (1b)

Following procedure A with 3-methylaniline (0.532 g, 5 mmol), the obtained crude was purified *via* flash chromatography (using dichloromethane and methanol), affording 2-*tert*-butylamino-6-[(3-methylphenyl)amino]thieno[3,2-d]pyrimidin-4(3*H*)-one as a beige powder (0.1 g, 31% yield). ^1^H NMR (DMSO-*d*_6_, 400 MHz) *δ* 10.09 (s, 1H), 9.52 (s, 1H), 7.24–7.17 (m, 1H), 7.04–6.99 (m, 2H), 6.78 (d, ^3^*J* = 7.5 Hz, 1H), 6.28 (s, 1H), 5.98 (bs, 1H), 2.29 (s, 3H), 1.39 (s, 9H). ^13^C NMR (DMSO-*d*_6_, 100 MHz) *δ* 160.3, 156.9, 154.9, 152.3, 141.9, 138.8, 129.3, 122.4, 117.6, 114.3, 102.3, 98.9, 50.9, 28.7 (3C), 21.1. Mp = 225–227 °C. HRMS (ESI) *m*/*z* calculated for C_17_H_21_N_4_OS [M + H]^+^ 329.1431, found 329.1428.

##### 2-*tert*-Butylamino-6-[(2-methylphenyl)amino]thieno[3,2-d]pyrimidin-4(3*H*)-one (1c)

Following procedure A with 2-methylaniline (0.532 mL, 5 mmol), the obtained crude was purified *via* flash chromatography (using dichloromethane and methanol). Fractions of interest were triturated in cyclohexane affording 2-*tert*-butylamino-6-[(2-methylphenyl)amino]thieno[3,2-d]pyrimidin-4(3*H*)-one as a brown powder (50 mg, 15% yield). ^1^H NMR (DMSO-*d*_6_, 400 MHz) *δ* 10.02 (s, 1H), 8.79 (s, 1H), 7.36 (d, ^3^*J* = 7.9 Hz, 1H), 7.27–7.17 (m, 2H), 7.04 (t, ^3^*J* = 7.4 Hz, 1H), 5.97 (s, 1H), 5.91 (s, 1H), 2.23 (s, 3H), 1.37 (s, 9H). ^13^C NMR (DMSO-*d*_6_, 100 MHz) *δ* 160.5, 157.8, 156.8, 152.2, 140.2, 131.1, 130.4, 126.8, 124.1, 121.4, 100.9, 98.6, 50.8, 28.7 (3C), 17.7. Mp = 159–161 °C. HRMS (ESI) *m*/*z* calculated for C_17_H_21_N_4_OS [M + H]^+^ 329.1431, found 329.1429.

##### 2-*tert*-Butylamino-6-[(4-methoxyphenyl)amino]thieno[3,2-d]pyrimidin-4(3*H*)-one (1d)

Following procedure A with 4-methoxyaniline (0.611 g, 5 mmol), the obtained crude was purified *via* two successive flash chromatography runs (using dichloromethane and methanol). Fractions of interest were triturated in diethyl ether affording 2-*tert*-butylamino-6-[(4-methoxyphenyl)amino]thieno[3,2-d]pyrimidin-4(3*H*)-one as a beige powder (49 mg, 14% yield). ^1^H NMR (DMSO-*d*_6_, 400 MHz) *δ* 10.02 (s, 1H), 9.33 (s, 1H), 7.20–7.13 (m, 2H), 6.96–6.90 (m, 2H), 6.11 (s, 1H), 5.92 (s, 1H), 3.73 (s, 3H), 1.38 (s, 9H). ^13^C NMR (DMSO-*d*_6_, 100 MHz) *δ* 160.6, 156.7, 156.7, 154.8, 152.3, 135.1, 120.1 (2C), 114.7 (2C), 100.2, 97.9, 55.3, 50.8, 28.7 (3C). Mp = 205–207 °C. HRMS (ESI) *m*/*z* calculated for C_17_H_21_N_4_O_2_S [M + H]^+^ 345.1380, found 345.1381.

##### 2-*tert*-Butylamino-6-[(4-fluorophenyl)amino]thieno[3,2-d]pyrimidin-4(3*H*)-one (1e)

Following procedure A with 4-fluoroaniline (0.470 mL, 5 mmol), the obtained crude was purified *via* two successive flash chromatography runs (using dichloromethane and methanol). Fractions of interest were triturated in diethyl ether, affording 2-*tert*-butylamino-6-[(4-fluorophenyl)amino]thieno[3,2-d]pyrimidin-4(3*H*)-one as a beige powder (77 mg, 23% yield). ^1^H NMR (DMSO-*d*_6_, 400 MHz) *δ* 10.10 (s, 1H), 9.53 (s, 1H), 7.26–7.20 (m, 2H), 7.20–7.13 (m, 2H), 6.24 (s, 1H), 5.96 (s, 1H), 1.38 (s, 9H). ^13^C NMR (DMSO-*d*_6_, 100 MHz) *δ* 160.4, 157.2 (d, ^1^*J* = 238 Hz, 1C), 156.8, 155.3, 152.3, 138.4 (d, ^4^*J* = 2.8 Hz, 1C), 119.2 (d, ^3^*J* = 8.0 Hz, 2C), 116.0 (d, ^2^*J* = 22.5 Hz, 2C), 101.8, 98.8, 50.8, 28.7 (3C). Mp = 195–197 °C. HRMS (ESI) *m*/*z* calculated for C_16_H_18_FN_4_OS [M + H]^+^ 333.1180, found 333.1182.

##### 2-*tert*-Butylamino-6-(morpholin-4-yl)thieno[3,2-d]pyrimidin-4(3*H*)-one (1f)

Following procedure A with morpholine (0.434 mL, 5 mmol), the obtained crude was purified *via* two successive flash chromatography runs (using dichloromethane and methanol), affording 2-*tert*-butylamino-6-(morpholin-4-yl)thieno[3,2-d]pyrimidin-4(3*H*)-one as a yellow solid (106 mg, 35% yield). ^1^H NMR (DMSO-*d*_6_, 400 MHz) *δ* 10.04 (bs, 1H), 6.07 (s, 1H), 5.94 (s, 1H), 3.75–3.68 (m, 4H), 3.25–3.17 (m, 4H), 1.38 (s, 9H). ^13^C NMR (DMSO-*d*_6_, 100 MHz) *δ* 163.6, 160.8, 156.8, 152.3, 99.5, 99.0, 65.3 (2C), 50.8, 49.1 (2C), 28.6 (3C). Mp = 264–266 °C. HRMS (ESI) *m*/*z* calculated for C_14_H_21_N_4_O_2_S [M + H]^+^ 309.1380, found 309.1379.

##### 2-*tert*-Butylamino-6-(piperidin-1-yl)thieno[3,2-*d*]pyrimidin-4(3*H*)-one (1g)

Following procedure A with piperidine (0.490 mL, 5 mmol), the obtained crude was purified *via* flash chromatography (using dichloromethane and methanol), affording 2-*tert*-butylamino-6-(piperidin-1-yl)thieno[3,2-*d*]pyrimidin-4(3*H*)-one as a pale-yellow powder (45 mg, 15% yield). ^1^H NMR (DMSO-*d*_6_, 400 MHz) *δ* 9.95 (s, 1H), 5.95 (s, 1H), 5.90 (s, 1H), 3.28–3.20 (m, 4H), 1.65–1.51 (m, 6H), 1.37 (s, 9H). ^13^C NMR (DMSO-*d*_6_, 100 MHz) *δ* 163.7, 161.3, 156.8, 152.2, 98.5, 98.2, 50.8, 50.2 (2C), 28.7 (3C), 24.5 (2C), 23.2. Mp = 171–173 °C. HRMS (ESI) *m*/*z* calculated for C_15_H_23_N_4_OS [M + H]^+^ 307.1587, found 307.1590.

#### 4.1.4. General procedure B for the synthesis of compounds through Sonogashira coupling

In a sealed microwave vial under nitrogen atmosphere, the appropriate 2-amino-6-bromothienopyrimidinone, the appropriate alkyne, copper iodide (16% mol), bis(triphenylphosphine)palladium(ii) dichloride (4% mol), and triethylamine (10 eq.) were dissolved in dry acetonitrile (1 : 1 v/v with triethylamine). The obtained suspension was stirred for 10 min under microwave irradiation at 100 °C. Water (20 times the quantity of acetonitrile) was added to the reaction mixture, which was then extracted with dichloromethane. The organic layer was washed with water and the excess was removed *in vacuo*. The obtained crude was purified *via* the appropriate method.

##### 3-Amino-5-[(4-methylphenyl)ethynyl]thiophene-2-carboxylate (2a)

Methyl-3-amino-5-bromothiophene-2-carboxylate (0.3 g, 1.27 mmol), 1-ethynyl-4-methylbenzene (0.155 g, 1.33 mmol), copper(i) iodide (39 mg, 0.2 mmol), triethylamine (1.76 mL, 12.7 mmol), tetrakis(triphenylphosphine)palladium(0) (4 mol%), and dried acetonitrile (0.5 mL) were put under nitrogen atmosphere. The obtained reaction mixture was stirred 10 min under microwave irradiation at 100 °C. Water (10 mL) was added and the mixture was extracted with ethyl acetate (3 × 10 mL). The combined organic layers were washed with brine (3 × 30 mL), dried over anhydrous sodium sulphate, and the excess solvent was removed *in vacuo*. The obtained residue was purified *via* column chromatography on silica gel (90 : 10 cyclohexane/ethyl acetate). The desired fractions were combined, the excess solvent removed *in vacuo*, and the obtained solid was triturated in cyclohexane, affording ethyl-3-amino-5-[(4-methylphenyl)ethynyl]thiophene-2-carboxylate as a brown powder (217 mg, 63% yield). ^1^H NMR (CDCl_3_, 400 MHz) *δ* 7.40 (d, ^3^*J* = 8.2 Hz, 2H), 7.16 (d, ^3^*J* = 8.2 Hz, 2H), 6.65 (s, 1H), 5.42 (bs, 2H), 3.83 (s, 3H), 2.37 (s, 3H). ^13^C NMR (CDCl_3_, 100 MHz) *δ* 164.5, 153.2, 139.5, 131.7 (2C), 129.4 (2C), 128.7, 123.3, 119.3, 102.1, 95.7, 81.9, 51.1, 21.7. Mp = 156–158 °C. HRMS (ESI) *m*/*z* calculated for C_15_H_14_NO_2_S [M + H]^+^ 272.0740, found 272.0743.

##### 2-*tert*-Butylamino-6-[(4-methylphenyl)ethynyl]thieno[3,2-*d*]pyrimidin-4(3*H*)-one (2b)

Following procedure B with 6-bromo-2-(tert-butylamino)thieno[3,2-d]pyrimidin-4(3H)-one (0.3 g, 0.99 mmol) and 1-ethynyl-4-methylbenzene (151 μL, 1.19 mmol), the obtained crude was purified *via* flash chromatography (using cyclohexane/ethyl acetate). Fractions of interest were combined, the excess solvent was removed *in vacuo*, and the solid triturated in diethyl ether, affording 2-*tert*-butylamino-6-[(4-methylphenyl)ethynyl]thieno[3,2-*d*]pyrimidin-4(3*H*)-one as a yellow/brown powder (200 mg, 60% yield). ^1^H NMR (DMSO-*d*_6_, 400 MHz) *δ* 10.59 (s, 1H), 7.49 (d, ^3^*J* = 10.8 Hz, 2H), 7.30–7.24 (m, 3H), 6.13 (s, 1H), 2.35 (s, 3H), 1.41 (s, 9H). ^13^C NMR (DMSO-*d*_6_, 100 MHz) *δ* 159.4, 157.7, 152.8, 140.2, 131.9 (2C), 130.0 (2C), 129.2, 129.1, 118.5, 113.7, 97.0, 82.3, 51.6, 29.0 (3C), 21.6. Mp = 235–237 °C. HRMS (ESI) *m*/*z* calculated for C_19_H_20_N_3_OS [M + H]^+^ 338.1322, found 338.1319.

##### 6-[(4-methylphenyl)ethynyl]-2-(propan-2-ylamino)thieno[3,2-*d*]pyrimidin-4(3*H*)-one (2c)

Following procedure B with 6-bromo-2-(propan-2-ylamino)thieno[3,2-*d*]pyrimidin-4(3*H*)-one (0.172 g, 0.60 mmol) and 1-ethynyl-4-methylbenzene (91 μL, 0.72 mmol), the obtained crude was purified *via* flash chromatography (cyclohexane and ethanol), affording 6-[(4-methylphenyl)ethynyl]-2-(propan-2-ylamino)thieno[3,2-*d*]pyrimidin-4(3H)-one as a yellow solid (44 mg, 23% yield). ^1^H NMR (DMSO-*d*_6_, 400 MHz) *δ* 10.78 (s, 1H), 7.48 (d, ^3^*J* = 8.1 Hz, 2H), 7.30–7.23 (m, 3H), 6.22 (d, ^3^*J* = 7.7 Hz, 1H), 4.08–3.94 (m, 1H), 2.35 (s, 3H), 1.17 (d, ^3^*J* = 6.5 Hz, 6H). ^13^C NMR (DMSO-*d*_6_, 100 MHz) *δ* 159.2, 157.3, 152.8, 139.7, 131.4 (2C), 129.5 (2C), 128.7, 128.5, 118.0, 113.2, 96.4, 81.9, 42.2, 22.4 (2C), 21.1. Mp = 277–279 °C. HRMS (ESI) *m*/*z* calculated for C_18_H_17_N_3_OS [M + H]^+^ 324.1165, found 324.1163.

##### 2-*tert*-Butylamino-6-(4-hydroxybut-1-yn-1-yl)thieno[3,2-*d*]pyrimidin-4(3*H*)-one (2d)

Following procedure B with 6-bromo-2-(*tert*-butylamino)thieno[3,2-d]pyrimidin-4(3*H*)-one (0.3 g, 0.99 mmol) and but-3-yn-1-ol (90 μL, 1.19 mmol), the obtained crude was triturated in acetonitrile. The precipitate was filtered and isolated, while the filtrate was purified *via* column chromatography over silica gel (starting from 100% dichloromethane to 97 : 3 dichloromethane/methanol), affording 2-*tert*-butylamino-6-(4-hydroxybut-1-yn-1-yl)thieno[3,2-*d*]pyrimidin-4(3*H*)-one as a brown powder (137 mg, 47% yield). ^1^H NMR (DMSO-*d*_6_, 400 MHz) *δ* 10.53 (bs, 1H), 7.09 (s, 1H), 6.09 (s, 1H), 4.95 (t, ^3^*J* = 5.6 Hz, 1H), 3.58 (q, ^3^*J* = 6.4 Hz, 2H), 2.63 (t, ^3^*J* = 6.4 Hz, 2H), 1.39 (s, 9H). ^13^C NMR (DMSO-*d*_6_, 100 MHz) *δ* 158.9, 157.1, 152.3, 129.6, 128.0, 112.4, 97.2, 74.2, 59.2, 51.0, 28.5 (3C), 23.6. Mp = 215–217 °C. HRMS (ESI) *m*/*z* calculated for C_14_H_18_N_3_O_2_S [M + H]^+^ 292.1114, found 292.1115.

##### 2-*tert*-Butylamino-6-(cyclopropylethynyl)thieno[3,2-*d*]pyrimidin-4(3*H*)-one (2e)

Following procedure B with 6-bromo-2-(propan-2-ylamino)thieno[3,2-*d*]pyrimidin-4(3*H*)-one (0.3 g, 0.99 mmol) and ethynylcyclopropane (336 μL, 3.97 mmol), the obtained crude was purified *via* flash chromatography (using cyclohexane and ethanol). Fractions of interest were triturated in cyclohexane, affording 2-*tert*-butylamino-6-(cyclopropylethynyl)thieno[3,2-*d*]pyrimidin-4(3*H*)-one as a beige powder (66 mg, 23% yield). ^1^H NMR (DMSO-*d*_6_, 400 MHz) *δ* 10.53 (s, 1H), 7.07 (s, 1H), 6.08 (s, 1H), 1.68–1.59 (m, 1H), 1.38 (s, 9H), 0.98–0.91 (m, 2H), 0.83–0.77 (m, 2H). ^13^C NMR (DMSO-*d*_6_, 100 MHz) *δ* 158.9, 157.1, 152.3, 129.7, 128.0, 112.3, 102.0, 68.9, 51.0, 28.5 (3C), 8.8 (2C), 0.0. Mp = 158–160 °C. HRMS (ESI) *m*/*z* calculated for C_15_H_17_N_3_OS [M + H]^+^ 288.1165, found 288.1166.

##### 2-*tert*-Butylamino-6-ethynylthieno[3,2-*d*]pyrimidin-4(3*H*)-one (2f)

Following procedure B with 6-bromo-2-(*tert*-butylamino)thieno[3,2-d]pyrimidin-4(3*H*)-one (0.3 g, 0.99 mmol) and ethynyltrimethylsilane (550 μL, 3.97 mmol), the obtained crude was purified *via* flash chromatography (using dichloromethane and methanol), affording 2-*tert*-butylamino-6-[(trimethylsilyl)ethynyl]thieno[3,2-*d*]pyrimidin-4(3*H*)-one as a yellow solid (198 mg), which was used without further purification. ^1^H NMR (DMSO-*d*_6_, 400 MHz) *δ* 10.59 (s, 1H), 7.22 (s, 1H), 6.11 (s, 1H), 1.39 (s, 9H), 0.25 (s, 9H).

To a solution of 2-(*tert*-butylamino)-6-((trimethylsilyl)ethynyl)thieno[3,2-*d*]pyrimidin-4(3*H*)-one (0.183 g, 0.57 mmol) in a mixture of dichloromethane and methanol (1 : 1 v/v, 0.2 M), potassium carbonate (0.396 g, 2.86 mmol) was added and the reaction mixture was stirred for 16 h at room temperature. The reaction mixture was poured into water and extracted with dichloromethane. The combined organic layers were dried over sodium sulphate and the excess solvent was removed *in vacuo*. The obtained crude was purified *via* column chromatography on silica gel (starting from 100% dichloromethane to 97 : 3 dichloromethane/methanol), affording 2-*tert*-butylamino-6-ethynylthieno[3,2-*d*]pyrimidin-4(3*H*)-one as a grey powder (150 mg, 92% yield). ^1^H NMR (DMSO-*d*_6_, 400 MHz) *δ* 10.60 (s, 1H), 7.27 (s, 1H), 6.13 (s, 1H), 4.87 (s, 1H), 1.39 (s, 9H). ^13^C NMR (DMSO-*d*_6_, 100 MHz) *δ* 158.6, 157.1, 152.4, 129.7, 127.7, 113.2, 88.5, 76.6, 51.1, 28.5 (3C). Mp = 195–197 °C. HRMS (ESI) *m*/*z* calculated for C_12_H_14_N_3_OS [M + H]^+^ 248.0852, found 248.0854.

##### 2-*tert*-Butylamino-6-[3-(4-methylpiperazin-1-yl)prop-1-yn-1-yl]thieno[3,2-*d*]pyrimidin-4(3*H*)-one (2g)

Following procedure B with 6-bromo-2-(*tert*-butylamino)thieno[3,2-d]pyrimidin-4(3*H*)-one (0.3 g, 0.99 mmol) and 1-methyl-4-(prop-2-yn-1-yl)piperazine (0.36 g, 2.60 mmol), the obtained crude was purified *via* flash chromatography (using dichloromethane and methanol), affording 2-*tert*-butylamino-6-[3-(4-methylpiperazin-1-yl)prop-1-yn-1-yl]thieno[3,2-*d*]pyrimidin-4(3*H*)-one as an orange solid (135 mg, 35% yield). ^1^H NMR (DMSO-*d*_6_, 400 MHz) *δ* 10.58 (bs, 1H), 7.18 (s, 1H), 6.13 (s, 1H), 3.57 (s, 2H), 2.34 (bs, 4H), 2.16 (s, 3H), 1.39 (s, 9H), 4H presumably situated below DMSO signal. ^13^C NMR (DMSO-*d*_6_, 100 MHz) *δ* 158.8, 157.1, 152.4, 128.7, 128.6, 112.7, 93.7, 77.9, 54.5, 51.2 (2C), 51.1 (2C), 46.8, 45.6, 28.5 (3C). Mp = 109–111 °C. HRMS (ESI) *m*/*z* calculated for C_18_H_26_N_5_OS [M + H]^+^ 360.1853, found 360.1853.

##### 6-(phenylethynyl)-2-(propan-2-ylamino)thieno[3,2-*d*]pyrimidin-4(3*H*)-one (2h)

Following procedure B with 6-bromo-2-(propan-2-ylamino)thieno[3,2-*d*]pyrimidin-4(3*H*)-one (0.3 g, 1.04 mmol) and ethynylbenzene (137.2 μL, 1.25 mmol), the obtained crude was triturated in diethyl ether, affording 6-(phenylethynyl)-2-(propan-2-ylamino)thieno[3,2-*d*]pyrimidin-4(3*H*)-one as a brown solid (285 mg, 85% yield). ^1^H NMR (DMSO-*d*_6_, 400 MHz) *δ* 10.94 (bs, 1H), 7.64–7.56 (m, 2H), 7.51–7.42 (m, 3H), 7.32 (s, 1H), 6.41 (bs, 1H), 4.08–3.95 (m, 1H), 1.17 (d, ^3^*J* = 6.4 Hz, 6H). ^13^C NMR (DMSO-*d*_6_, 100 MHz) *δ* 159.1, 157.3, 153.1, 131.5 (2C), 129.7, 128.9 (2C), 128.7, 128.2, 121.0, 113.4, 96.0, 82.4, 42.1, 20.8 (2C). Mp = 276–278 °C. HRMS (ESI) *m*/*z* calculated for C_17_H_16_N_3_OS [M + H]^+^ 310.1009, found 310.1003.

#### 4.1.5. General procedure C for the synthesis of thiophene aminoesters functionalized at position 5 through Suzuki–Miyaura coupling

To a mixture of dioxane (0.5 mL per 100 mg) and water (0.25 mL per 100 mg) in a sealed microwave vial, methyl-3-amino-5-bromothiophene-2-carboxylate (1 eq.), appropriate boronic acid (1.6 eq.), and potassium carbonate (2 eq.) were added. The mixture was then put under nitrogen atmosphere and tetrakis(triphenylphosphine)palladium(0) (0.05% mol) was added, and the mixture was stirred at 75 °C for the appropriate time. The reaction mixture was poured into cold water.

•If a precipitate was formed, this precipitate was filtered, washed with water, and dried *in vacuo*. If needed, the precipitate was then purified with the appropriate method.

•If no precipitate was formed, the mixture was extracted with ethyl acetate. The organic layer was washed with brine, dried over sodium sulphate, and the excess solvent was removed *in vacuo*. Then, the obtained crude was purified with the appropriate method.

##### Methyl-3-amino-5-[4-(methoxycarbonyl)phenyl]thiophene-2-carboxylate (3a)

Following procedure C starting from methyl-3-aminothiophene-2-carboxylate (0.5 g, 2.12 mmol) and 4-methoxycarbonylphenylboronic acid (0.610, 3.39 mmol) with a reaction time of 6 h, the obtained crude was purified *via* column chromatography on silica gel (deactivated with 3% triethylamine using 80 : 20 cyclohexane/dichloromethane), affording methyl 3-amino-5-[4-(methoxycarbonyl)phenyl]thiophene-2-carboxylate as a yellow solid (340 mg, 55% yield). ^1^H NMR (CDCl_3_, 250 MHz) *δ* 8.05 (d, ^3^*J* = 8.5 Hz, 2H), 7.64 (d, ^3^*J* = 8.5 Hz, 2H), 6.86 (s, 1H), 5.49 (bs, 2H), 3.93 (s, 3H), 3.85 (s, 3H). ^13^C NMR (CDCl_3_, 62.5 MHz) *δ* 166.6, 164.9, 154.3, 147.5, 137.6, 130.4 (2C), 130.3, 125.9 (2C), 116.7, 101.7, 52.4, 51.6. Mp = 190–192 °C. HRMS (ESI) *m*/*z* calculated for C_14_H_14_NO_4_S [M + H]^+^ 292.0638, found 292.0637.

##### Methyl-3-amino-5-(4-nitrophenyl)thiophene-2-carboxylate (3b)

Following procedure C starting from methyl-3-aminothiophene-2-carboxylate (0.4 g, 1.69 mmol) and 4-nitrophenylboronic acid (0.453 g, 2.71 mmol) with a reaction time of 18 h, the obtained crude was purified *via* column chromatography on silica gel (starting from 75 : 25 cyclohexane/ethyl acetate to 50 : 50 cyclohexane/ethyl acetate), affording methyl-3-amino-5-(4-nitrophenyl)thiophene-2-carboxylate as an orange solid (316 mg, 65% yield), which was used without further purification. ^1^H NMR (DMSO-*d*_6_, 250 MHz) *δ* 8.32–8.23 (m, 2H), 7.95–7.87 (m, 2H), 7.18 (s, 1H), 6.68 (s, 2H), 3.76 (s, 3H). ^13^C NMR (DMSO-*d*_6_, 75 MHz) *δ* 163.8, 155.3, 147.2, 144.3, 138.8, 126.6 (2C), 124.5 (2C), 118.7, 98.8, 51.2. Mp = 212–214 °C.

##### Methyl-3-amino-5-(4-hydroxyphenyl)thiophene-2-carboxylate (3c)

Following procedure C starting from methyl-3-aminothiophene-2-carboxylate (0.8 g, 3.39 mmol) and 4-hydroxyphenylboronic acid (0.748 g, 5.42 mmol) with a reaction time of 24 h, the obtained crude was purified *via* flash chromatography (using cyclohexane and ethyl acetate), affording methyl-3-amino-5-(4-hydroxyphenyl)thiophene-2-carboxylate as a light brown solid (540 mg, 64% yield). ^1^H NMR (DMSO-*d*_6_, 400 MHz) *δ* 9.86 (s, 1H), 7.45 (d, ^3^*J* = 8.6 Hz, 2H), 6.82 (d, ^3^*J* = 8.6 Hz, 2H), 6.79 (s, 1H), 6.54 (bs, 2H), 3.71 (s, 3H). ^13^C NMR (DMSO-*d*_6_, 100 MHz) *δ* 164.0, 158.6, 155.8, 148.4, 127.1 (2C), 123.8, 115.9 (2C), 114.3, 95.3, 50.8. Mp = 175–177 °C. HRMS (ESI) *m*/*z* calculated for C_12_H_11_NO_3_SNa [M + Na]^+^ 272.0352, found 272.0352.

##### Methyl-3-amino-5-(3-chloro-4-fluorophenyl)thiophene-2-carboxylate (3d)

Following procedure C starting from methyl-3-aminothiophene-2-carboxylate (0.8 g, 3.39 mmol) and (3-chloro-4-fluorophenyl)boronic acid (0.945 g, 5.42 mmol) with a reaction time of 24 h, the obtained crude was purified *via* column chromatography on silica gel (starting from 100% cyclohexane to 80 : 20 cyclohexane/ethyl acetate), affording methyl-3-amino-5-(3-chloro-4-fluorophenyl)thiophene-2-carboxylate as a beige solid (790 mg, 82% yield). ^1^H NMR (DMSO-*d*_6_, 400 MHz) *δ* 7.61 (dd, ^3^*J* = 6.8 Hz, ^4^*J* = 2.3 Hz, 1H), 7.42 (ddd, ^3^*J* = 8.6 Hz, ^4^*J* = 4.4 Hz, ^4^*J* = 2.3 Hz, 1H), 7.15 (t, ^3^*J* = 8.6 Hz, 1H), 6.71 (s, 1H), 3.85 (s, 3H). ^13^C NMR (DMSO-*d*_6_, 100 MHz) *δ* 164.9, 158.5 (d, ^1^*J* = 252 Hz, 1C), 154.3, 146.4, 130.9 (d, ^3^*J* = 4.3 Hz, 1C), 128.6, 128.3, 125.9 (d, ^3^*J* = 7.3 Hz, 1C), 121.9 (d, ^2^*J* = 18.2 Hz, 1C), 117.3 (d, ^2^*J* = 21.7 Hz, 1C), 116.2, 51.5. Mp = 161–163 °C. HRMS (ESI) *m*/*z* calculated for C_12_H_10_ClFNO_2_S [M + H]^+^ 286.0099, found 286.0102.

##### Methyl-3-amino-5-[4-(morpholin-4-yl)phenyl]thiophene-2-carboxylate (3e)

Following procedure C starting from methyl 3-aminothiophene-2-carboxylate (0.4 g, 1.69 mmol) and (4-(morpholin-4-yl)phenyl)boronic acid (0.561 g, 2.71 mmol) with a reaction time of 16 h, the obtained crude was purified *via* flash chromatography (using cyclohexane/ethyl acetate). Fractions of interest were combined and triturated in diethyl ether, affording methyl-3-amino-5-[4-(morpholin-4-yl)phenyl]thiophene-2-carboxylate as a yellow powder (0.395 g, 73% yield). ^1^H NMR (CDCl_3_, 400 MHz) *δ* 7.50 (d, ^3^*J* = 8.8 Hz, 2H), 6.89 (d, ^3^*J* = 8.8 Hz, 2H), 6.56 (s, 1H), 5.46 (bs, 2H), 3.90–3.85 (m, 4H), 3.83 (s, 3H), 3.25–3.17 (m, 4H). ^13^C NMR (CDCl_3_, 100 MHz) *δ* 165.1, 154.7, 151.7, 149.6, 127.1 (2C), 124.9, 115.4 (2C), 114.1, 99.4, 66.8, 51.3 (2C), 48.8 (2C). Mp = 208–210 °C. HRMS (ESI) *m*/*z* calculated for C_16_H_19_NO_3_S [M + H]^+^ 319.1111, found 319.1110.

##### Methyl-3-amino-5-[4-(4-methylpiperazin-1-yl)phenyl]thiophene-2-carboxylate (3f)

Following procedure C starting from methyl-3-aminothiophene-2-carboxylate (0.4 g, 1.69 mmol) and (4-(4-methylpiperazin-1-yl)phenyl)boronic acid (0.597 g, 2.71 mmol) with a reaction time of 16 h, the obtained crude was purified *via* flash chromatography (using dichloromethane and methanol). Fractions of interest were combined and triturated in diethyl ether affording methyl-3-amino-5-[4-(4-methylpiperazin-1-yl)phenyl]thiophene-2-carboxylate as a yellow powder (0.4 g, 71% yield). ^1^H NMR (CDCl_3_, 400 MHz) *δ* 7.47 (d, ^3^*J* = 8.8 Hz, 2H), 6.89 (d, ^3^*J* = 8.8 Hz, 2H), 6.64 (s, 1H), 5.46 (s, 2H), 3.83 (s, 3H), 3.30–3.23 (m, 4H), 2.60–2.53 (m, 4H), 2.35 (s, 3H). ^13^C NMR (CDCl_3_, 100 MHz) *δ* 165.1, 154.7, 151.7, 149.8, 127.0 (2C), 124.3, 115.5 (2C), 113.9, 99.1, 55.0 (2C), 51.3, 48.4 (2C), 46.3. Mp = 191–193 °C. HRMS (ESI) *m*/*z* calculated for C_17_H_22_N_3_O_2_S [M + H]^+^ 332.1427, found 332.1427.

##### Methyl-3-amino-5-[4-(piperidin-1-yl)phenyl]thiophene-2-carboxylate (3g)

Following procedure C starting from methyl 3-aminothiophene-2-carboxylate (0.4 g, 1.69 mmol) and (4-(piperidin-1-yl)phenyl)boronic acid (0.556 g, 2.71 mmol) with a reaction time of 16 h, the obtained crude was purified *via* flash chromatography (using cyclohexane/ethyl acetate). Fractions of interest were combined and triturated in diethyl ether affording methyl-3-amino-5-[4-(piperidin-1-yl)phenyl]thiophene-2-carboxylate as a yellow powder (0.38 g, 71% yield). ^1^H NMR (CDCl_3_, 400 MHz) *δ* 7.46 (d, ^3^*J* = 8.8 Hz, 2H), 6.89 (d, ^3^*J* = 8.8 Hz, 2H), 6.63 (s, 1H), 5.45 (s, 2H), 3.83 (s, 3H), 3.29–3.18 (m, 4H), 1.76–1.53 (m, 6H). ^13^C NMR (CDCl_3_, 100 MHz) *δ* 165.2, 154.8, 152.4, 150.1, 127.0 (2C), 123.5, 115.7 (2C), 113.6, 98.9, 51.3 (2C), 49.8, 25.7 (2C), 24.4. Mp = 191–193 °C. HRMS (ESI) *m*/*z* calculated for C_17_H_21_N_2_O_2_S [M + H]^+^ 317.1318, found 317.1319.

##### Methyl-3-amino-5-[4-(methylsulfanyl)phenyl]thiophene-2-carboxylate (3h)

Following procedure C starting from methyl-3-aminothiophene-2-carboxylate (0.8 g, 3.39 mmol) and [4-(methylsulfanyl)phenyl]boronic acid (0.911 g, 5.42 mmol) with a reaction time of 24 h, the obtained crude was purified *via* flash chromatography (using cyclohexane/ethyl acetate), affording methyl-3-amino-5-[4-(methylsulfanyl)phenyl]thiophene-2-carboxylate as a pale-yellow solid (0.678 g, 72% yield). ^1^H NMR (CDCl_3_, 400 MHz) *δ* 7.52–7.46 (m, 2H), 7.26–7.21 (m, 2H), 6.73 (s, 1H), 3.84 (s, 3H), 2.50 (s, 3H). ^13^C NMR (CDCl_3_, 100 MHz) *δ* 165.1, 154.5, 148.8, 140.2, 130.1, 126.6 (2C), 126.3 (2C), 115.2, 100.2, 51.4, 15.6. Mp = 173–175 °C. HRMS (ESI) *m*/*z* calculated for C_13_H_14_NO_2_S_2_ [M + H]^+^ 280.0460, found 280.0460.

##### Methyl-3-amino-5-[4-(methylsulfonyl)phenyl]thiophene-2-carboxylate (3i)

Following procedure C starting from methyl 3-aminothiophene-2-carboxylate (0.4 g, 1.69 mmol) and [4-(methylsulfonyl)phenyl]boronic acid (0.542 g, 2.71 mmol) with a reaction time of 4 h, the obtained crude was purified *via* flash chromatography (using cyclohexane/ethyl acetate), affording methyl-3-amino-5-[4-(methylsulfonyl)phenyl]thiophene-2-carboxylate as a yellow solid (0.325 g, 62% yield). ^1^H NMR (CDCl_3_, 400 MHz) *δ* 7.96 (d, ^3^*J* = 8.5 Hz, 2H), 7.75 (d, ^3^*J* = 8.5 Hz, 2H), 6.88 (s, 1H), 3.86 (s, 3H), 3.07 (s, 3H). ^13^C NMR (CDCl_3_, 100 MHz) *δ* 164.8, 154.2, 146.1, 140.4, 138.8, 128.4 (2C), 126.7 (2C), 117.4, 102.4, 51.6, 44.7. Mp = 213–214 °C. HRMS (ESI) *m*/*z* calculated for C_14_H_14_NO_4_S_2_ [M + H]^+^ 312.0359, found 312.0359.

##### Methyl-3-amino-5-(4-sulfamoylphenyl)thiophene-2-carboxylate (3j)

Following procedure C starting from methyl 3-aminothiophene-2-carboxylate (0.4 g, 1.69 mmol) and (4-sulfamoylphenyl)boronic acid (0.545, 2.71 mmol) with a reaction time of 16 h, the obtained crude was purified *via* flash chromatography (using cyclohexane/ethyl acetate), affording methyl-3-amino-5-(4-sulfamoylphenyl)thiophene-2-carboxylate as a yellow powder (0.249 g, 47%), which was used without further purification. ^1^H NMR (DMSO-*d*_6_, 400 MHz) *δ* 7.90–7.79 (m, 4H), 7.44 (s, 2H), 7.09 (s, 1H), 6.64 (s, 2H), 3.75 (s, 3H). ^13^C NMR (DMSO-*d*_6_, 100 MHz) *δ* 163.8, 155.4, 145.5, 144.1, 135.7, 126.6 (2C), 126.0 (2C), 117.6, 97.7, 51.1. Mp = 225–227 °C. HRMS (ESI) *m*/*z* calculated for C_12_H_13_N_2_O_4_S_2_ [M + H]^+^ 313.0311, found 313.0312.

##### Methyl-3-amino-5-[4-(morpholin-4-ylsulfonyl)phenyl]thiophene-2-carboxylate (3k)

Following procedure C starting from methyl 3-aminothiophene-2-carboxylate (0.4 g, 1.69 mmol) and [4-(morpholinosulfonyl)phenyl]boronic acid (0.734 g, 2.71 mmol) with a reaction time of 24 h, the obtained crude was purified *via* column chromatography on silica gel (starting from 50 : 50) (cyclohexane/ethyl acetate to 100% ethyl acetate), affording methyl-3-amino-5-[4-(morpholin-4-ylsulfonyl)phenyl]thiophene-2-carboxylate as an orange powder (570 mg, 88% yield). ^1^H NMR (DMSO-*d*_6_, 400 MHz) *δ* 7.90 (d, ^3^*J* = 8.5 Hz, 2H), 7.78 (d, ^3^*J* = 8.5 Hz, 2H), 7.15 (s, 1H), 6.65 (s, 2H), 3.75 (s, 3H), 3.67–3.60 (m, 4H), 2.94–2.86 (m, 4H). ^13^C NMR (DMSO-*d*_6_, 100 MHz) *δ* 163.8, 155.3, 145.0, 137.2, 134.4, 128.6 (2C), 126.4 (2C), 118.1, 98.2, 65.3 (2C), 51.2, 45.9 (2C). Mp = 272–274 °C. HRMS (ESI) *m*/*z* calculated for C_16_H_19_N_2_O_5_S_2_ [M + H]^+^ 405.0549, found 405.0546.

##### Methyl-3-amino-5-(2-chloropyrimidin-5-yl)thiophene-2-carboxylate (3l)

To a solution of methyl-3-aminothiophene-2-carboxylate (0.5 g, 2.12 mmol) in dioxane (3.75 mL), (2-chloropyrimidin-5-yl)boronic acid (0.537 g, 3.39 mmol) and potassium carbonate (0.585 g, 4.24 mmol) were added and the solution was deoxygenated with nitrogen during 5 min. Tetrakis(triphenylphosphine)palladium(0) (0.05% mol) was added, the mixture was put under nitrogen atmosphere, and stirred at 85 °C for 24 h. The reaction mixture was poured into water (75 mL) and extracted with ethyl acetate (3 × 75 mL). The combined organic phases were washed with brine (3 × 150 mL), dried over sodium sulphate, and the excess solvent was removed *in vacuo*. The obtained crude was purified *via* column chromatography on silica gel (using 100% dichloromethane), affording methyl 3-amino-5-(2-chloropyrimidin-5-yl)thiophene-2-carboxylate as a yellow powder (0.25 g, 44% yield). ^1^H NMR (CDCl_3_, 400 MHz) *δ* 8.79 (s, 2H), 6.84 (s, 1H), 5.55 (bs, 2H), 3.86 (s, 3H). ^13^C NMR (CDCl_3_, 100 MHz) *δ* 164.6, 161.0, 156.2 (2C), 154.1, 139.2, 126.7, 117.7, 102.6, 51.8. Mp = 176–178 °C. HRMS (ESI) *m*/*z* calculated for C_10_H_9_ClN_3_O_2_S [M + H]^+^ 270.0099, found 270.0097.

#### 4.1.6. Substitution reactions on methyl-3-amino-5-(2-chloropyrimidin-5-yl)thiophene-2-carboxylate

##### Methyl-3-amino-5-[2-(morpholin-4-yl)pyrimidin-5-yl]thiophene-2-carboxylate (3m)

To a solution of methyl-3-amino-5-(2-chloropyrimidin-5-yl)thiophene-2-carboxylate (0.25 g, 0.93 mmol) in ethanol (10 mL), morpholine (77.3 μL, 0.88 mmol) and triethylamine (0.33 mL, 2.38 mmol) were added. The reaction mixture was stirred for 3 h at 80 °C. After being allowed to cool to room temperature, the reaction mixture was filtered and the obtained precipitate was washed with ethanol, brine, and water, affording methyl 3-amino-5-[2-(morpholin-4-yl)pyrimidin-5-yl]thiophene-2-carboxylate as a white solid (0.225 g, 80% yield). ^1^H NMR (DMSO-*d*_6_, 400 MHz) *δ* 8.66 (s, 2H), 6.88 (s, 1H), 6.60 (s, 2H), 3.79–3.74 (m, 4H), 3.72 (s, 3H), 3.69–3.64 (m, 4H). ^13^C NMR (DMSO-*d*_6_, 100 MHz) *δ* 163.8, 160.7, 155.6, 155.0 (2C), 142.4, 116.2, 114.9, 95.6, 65.9 (2C), 50.9, 44.0 (2C). Mp = 170–172 °C. HRMS (ESI) *m*/*z* calculated for C_14_H_17_N_4_O_3_S [M + H]^+^ 321.1016, found 321.1017.

##### Methyl-3-amino-5-[2-(4-methylpiperazin-1-yl)pyrimidin-5-yl]thiophene-2-carboxylate (3n)

To a solution of methyl-3-amino-5-(2-chloropyrimidin-5-yl)thiophene-2-carboxylate (0.25 g, 0.93 mmol) in ethanol (10 mL), *N*-methylpyperazine (97.4 μL, 0.88 mmol) and triethylamine (0.33 mL, 2.38 mmol) were added. The reaction mixture was stirred for 16 h at 80 °C. The reaction mixture was poured into water (100 mL) and extracted with ethyl acetate (3 × 100 mL). The combined organic layers were washed with water (2 × 200 mL), dried over sodium sulphate, and the excess solvent was removed *in vacuo*, affording methyl-3-amino-5-[2-(4-methylpiperazin-1-yl)pyrimidin-5-yl]thiophene-2-carboxylate as a yellow powder (215 mg, 73% yield), which was used without further purification. ^1^H NMR (DMSO-*d*_6_, 400 MHz) *δ* 8.63 (s, 2H), 6.87 (s, 1H), 6.60 (s, 2H), 3.82–3.75 (m, 4H), 3.72 (s, 3H), 2.41–2.34 (m, 4H), 2.22 (s, 3H). ^13^C NMR (DMSO-*d*_6_, 100 MHz) *δ* 164.3, 161.1, 156.1, 155.5 (2C), 143.0, 116.4, 115.2, 95.9, 54.7 (2C), 51.4, 46.2, 43.8 (2C). Mp = 148–150 °C. HRMS (ESI) *m*/*z* calculated for C_15_H_20_N_5_O_2_S [M + H]^+^ 334.1332, found 334.1331.

#### 4.1.7. General procedure D for cyclization reactions

To a solution of the appropriate functionalized methyl-3-aminothiophene-2-carboxylate (1 eq.) in *N*,*N*-dimethylformamide (2.25 mL per 100 mg), ethoxycarbonyl isothiocyanate (1 eq.) was added and the solution was stirred for 2 h at room temperature. To the reaction mixture, *tert*-butylamine or isopropylamine (2 eq.), triethylamine (3 eq.), and *N*-(3-dimethylaminopropyl)-*N*′-ethylcarbodiimide hydrochloride (1.05 eq.) were added and was stirred for 16 h at room temperature. The reaction mixture was then stirred at 170 °C under microwave irradiation for 2 h. The reaction mixture was poured into cold water.

•If a precipitate was formed, this precipitate was filtered, washed with water, and dried *in vacuo*. If needed, the precipitate was then purified with the appropriate method.

•If no precipitate was obtained, the mixture was extracted with ethyl acetate. The organic layer was washed with brine, dried over sodium sulphate, and the excess solvent was removed *in vacuo*. Then, the obtained crude was purified with the appropriate method.

##### 6-Bromo-2-(*tert*-butylamino)thieno[3,2-*d*]pyrimidin-4(3*H*)-one (1)

Following procedure D with methyl-3-amino-5-bromothiophene-2-carboxylate (2 g, 8.47 mmol) and *tert*-butylamine (1.78 mL, 16.9 mmol), the obtained crude was triturated in acetonitrile, affording 6-Bromo-2-(*tert*-butylamino)thieno[3,2-*d*]pyrimidin-4(3*H*)-one as a beige powder (1.3 g, 51% yield). ^1^H NMR (DMSO-*d*_6_, 250 MHz) *δ* 10.53 (bs, 1H), 7.23 (s, 1H), 6.12 (s, 1H), 1.39 (s, 9H). ^13^C NMR (DMSO-*d*_6_, 62.5 MHz) *δ* 159.3, 156.5, 152.4, 127.8, 121.9, 113.4, 51.1, 28.5 (3C). Mp: degradation observed at 162–164 °C. HRMS (ESI) *m*/*z* calculated for C_10_H_13_BrN_3_OS [M + H]^+^ 301.9957, found 301.9954.

##### 6-Bromo-2-(propan-2-ylamino)thieno[3,2-*d*]pyrimidin-4(3*H*)-one (3)

Following procedure D with methyl 3-amino-5-bromothiophene-2-carboxylate (1 g, 4.24 mmol) and with isopropylamine (0.728 mL, 8.47 mmol), the obtained crude was triturated in acetonitrile. The precipitate was isolated and the filtrate was further purified *via* flash chromatography (using dichloromethane and methanol), affording 6-bromo-2-(propan-2-ylamino)thieno[3,2-*d*]pyrimidin-4(3*H*)-one as a beige powder (0.695 g, 57% yield). ^1^H NMR (DMSO-*d*_6_, 250 MHz) *δ* 10.72 (bs, 1H), 7.25 (s, 1H), 6.21 (d, ^3^*J* = 7.6 Hz, 1H), 3.99 (m, 1H), 1.16 (d, ^3^*J* = 6.5 Hz, 6H). ^13^C NMR (DMSO-*d*_6_, 62.5 MHz) *δ* 161.2, 156.7, 152.9, 127.5, 122.0, 113.3, 42.2, 22.3 (2C). Mp = 240–242 °C. HRMS (ESI) *m*/*z* calculated for C_9_H_11_BrN_3_OS [M + H]^+^ 287.9801, found 287.9801.

##### Methyl-4-(2-*tert*-butylamino-4-oxo-3,4-dihydrothieno[3,2-*d*]pyrimidin-6-yl)benzoate (4a)

Following procedure D with methyl-3-amino-5-(4-(methoxycarbonyl)phenyl)thiophene-2-carboxylate (0.2 g, 0.69 mmol) and with *tert*-butylamine (144 μL, 1.37 mmol), the obtained crude was triturated in methanol, affording methyl-4-(2-*tert*-butylamino-4-oxo-3,4-dihydrothieno[3,2-*d*]pyrimidin-6-yl)benzoate as a beige powder (0.134 mg, 55% yield). ^1^H NMR (DMSO-*d*_6_, 400 MHz) *δ* 10.54 (bs, 1H), 8.01 (d, ^3^*J* = 8.5 Hz, 2H), 7.94 (d, ^3^*J* = 8.5 Hz, 2H), 7.66 (s, 1H), 6.12 (s, 1H), 3.87 (s, 3H), 1.42 (s, 9H). ^13^C NMR (DMSO-*d*_6_, 100 MHz) *δ* 165.7, 160.0, 157.6, 152.3, 148.1, 137.2, 130.0 (2C), 129.7, 126.1 (2C), 122.3, 112.6, 52.3, 51.0, 28.6 (3C). Mp > 300 °C. HRMS (ESI) *m*/*z* calculated for C_18_H_20_N_3_O_3_S [M + H]^+^ 358.1220, found 358.1216.

##### 2-*tert*-Butylamino-6-(4-nitrophenyl)thieno[3,2-*d*]pyrimidin-4(3*H*)-one (4b)

Following procedure D with methyl 3-amino-5-(4-nitrophenyl)thiophene-2-carboxylate (0.41 g, 1.47 mmol) and with *tert*-butylamine (313 μL, 2.95 mmol), the obtained crude was purified *via* column chromatography on silica gel (starting from 60 : 40 to 50 : 50 cyclohexane/ethyl acetate), affording 2-*tert*-butylamino-6-(4-nitrophenyl)thieno[3,2-*d*]pyrimidin-4(3*H*)-one as an orange solid (0.277 g, 55% yield). ^1^H NMR (DMSO-*d*_6_, 400 MHz) *δ* 10.56 (s, 1H), 8.26 (d, ^3^*J* = 8.8 Hz, 2H), 8.06 (d, ^3^*J* = 9.2 Hz, 2H), 7.76 (s, 1H), 6.13 (s, 1H), 1.43 (s, 9H). ^13^C NMR (DMSO-*d*_6_, 100 MHz) *δ* 159.9, 157.5, 152.3, 147.2, 146.7, 139.1, 126.8 (2C), 124.3 (2C), 123.5, 113.5, 51.0, 28.5 (3C). Mp > 300 °C. HRMS (ESI) *m*/*z* calculated for C_16_H_17_N_4_O_3_S [M + H]^+^ 345.1016, found 345.1016.

##### 6-(4-nitrophenyl)-2-(propan-2-ylamino)thieno[3,2-*d*]pyrimidin-4(3*H*)-one (4c)

Following procedure D with methyl 3-amino-5-(4-nitrophenyl)thiophene-2-carboxylate (0.4 g, 1.44 mmol) and with isopropylamine (244 μL, 2.88 mmol), the obtained crude was purified *via* column chromatography on silica gel (staring from 70 : 30 to 50 : 50 dichloromethane/ethyl acetate), affording 6-(4-nitrophenyl)-2-(propan-2-ylamino)thieno[3,2-*d*]pyrimidin-4(3*H*)-one as an orange solid (0.156 g, 33% crude yield), which was used without further purifications. ^1^H NMR (DMSO-*d*_6_, 400 MHz) *δ* 10.79 (s, 1H), 8.31–8.25 (m, 2H), 8.09–8.02 (m, 2H), 7.79 (s, 1H), 6.26 (d, ^3^*J* = 7.1 Hz, 1H), 4.08–3.98 (m, 1H), 1.19 (d, ^3^*J* = 6.5 Hz, 6H).

##### 2-(*tert*-butylamino)-6-(4-hydroxyphenyl)thieno[3,2-*d*]pyrimidin-4(3*H*)-one (4d)

Following procedure D with methyl-3-amino-5-(4-hydroxyphenyl)thiophene-2-carboxylate (0.44 g, 1.77 mmol) and with *tert*-butylamine (371 μL, 3.53 mmol), the obtained crude was purified *via* flash chromatography (using dichloromethane and methanol), affording 2-(*tert*-butylamino)-6-(4-hydroxyphenyl)thieno[3,2-*d*]pyrimidin-4(3*H*)-one as a light brown solid (70 mg, 13%). ^1^H NMR (DMSO-*d*_6_, 400 MHz) *δ δ* 10.39 (bs, 1H), 9.89 (bs, 1H), 7.61 (d, ^3^*J* = 8.7 Hz, 2H), 7.30 (s, 1H), 6.83 (d, ^3^*J* = 8.7 Hz, 2H), 6.05 (s, 1H), 1.42 (s, 3H). ^13^C NMR (DMSO-d6, 100 MHz) *δ* 160.9, 159.1, 157.9, 152.6, 151.2, 127.9 (2C), 124.5, 118.9, 116.4 (2C), 110.6, 51.4, 29.1. Mp = 276–278 °C. HRMS (ESI) *m*/*z* calculated for C_16_H_18_N_3_O_2_S [M + H]+ 316.1120, found 316.1123.

##### 2-*tert*-Butylamino-6-(3-chloro-4-fluorophenyl)thieno[3,2-*d*]pyrimidin-4(3*H*)-one (4e)

Following procedure D with methyl-3-amino-5-(3-chloro-4-fluorophenyl)thiophene-2-carboxylate (0.3 g, 1.05 mmol) and *tert*-butylamine (220 μL, 2.1 mmol), the obtained crude was triturated in diethyl ether, then purified *via* flash chromatography (using dichloromethane and methanol), affording 2-*tert*-butylamino-6-(3-chloro-4-fluorophenyl)thieno[3,2-*d*]pyrimidin-4(3*H*)-one as a white powder (180 mg, 49% yield). ^1^H NMR (DMSO-*d*_6_, 400 MHz) *δ* 10.51 (s, 1H), 8.07 (dd, ^4^*J* = 7 Hz, ^4^*J* = 2.4 Hz, 1H), 7.79 (ddd, ^3^*J* = 8.6 Hz, ^4^*J* = 4.6 Hz, ^4^*J* = 2.4 Hz, 1H), 7.61 (s, 1H), 7.50 (t, ^3^*J* = 9 Hz, 1H), 6.11 (s, 1H), 1.42 (s, 9H). ^13^C NMR (DMSO-*d*_6_, 100 MHz) *δ* 160.1, 157.5 (d, ^1^*J* = 250 Hz, 1C), 157.5, 152.3, 147.0, 130.9 (d, ^3^*J* = 3.7 Hz, 1C), 127.9, 126.7 (d, ^3^*J* = 7.5 Hz, 1C), 121.9, 120.6 (d, ^2^*J* = 18.1 Hz, 1C), 117.7 (d, ^3^*J* = 21.6, 1C), 112.1, 51.0, 28.6 (3C). Mp = 259–261 °C. HRMS (ESI) *m*/*z* calculated for C_16_H_16_ClFN_3_OS [M + H]^+^ 352.0681, found 352.0681.

##### 2-(Propan-2-ylamino)-6-(3-chloro-4-fluorophenyl)thieno[3,2-*d*]pyrimidin-4(3*H*)-one (4f)

Following procedure D with methyl-3-amino-5-(3-chloro-4-fluorophenyl)thiophene-2-carboxylate (0.3 g, 1.05 mmol) and isopropylamine (180 μL, 2.1 mmol), the obtained crude was purified *via* flash chromatography (using dichloromethane and methanol). Fractions of interest were triturated in diethyl ether, affording 2-(propan-2-ylamino)-6-(3-chloro-4-fluorophenyl)thieno[3,2-*d*]pyrimidin-4(3*H*)-one as a grey solid (130 mg, 37% yield). ^1^H NMR (DMSO-*d*_6_, 400 MHz) *δ* 10.67 (s, 1H), 8.04 (dd, ^4^*J* = 7 Hz, ^4^*J* = 2.4 Hz, 1H), 7.78 (ddd, ^3^*J* = 8.7 Hz, ^4^*J* = 4.6 Hz, ^4^*J* = 2.4 Hz, 1H), 7.63 (s, 1H), 7.51 (t, ^3^*J* = 8.9 Hz, 1H), 6.15 (d, ^3^*J* = 7.5 Hz, 1H), 4.09–3.94 (m, 1H), 1.18 (d, ^3^*J* = 6.5 Hz, 6H). ^13^C NMR (DMSO-*d*_6_, 100 MHz) *δ* 160.4, 157.6, 157.5 (d, ^1^*J* = 250 Hz, 1C), 152.7, 147.1, 131.0 (d, ^3^*J* = 3.9 Hz, 1C), 127.9, 126.7 (d, ^3^*J* = 7.5 Hz, 1C), 121.6, 120.6 (d, ^2^*J* = 18.1 Hz, 1C), 117.7 (d, ^2^*J* = 21.5 Hz, 1C), 112.0, 42.2, 22.4 (2C). Mp > 300 °C. HRMS (ESI) *m*/*z* calculated for C_15_H_14_ClFN_3_OS [M + H]^+^ 338.0525, found 338.0522.

##### 2-*tert*-Butylamino-6-[4-(morpholin-4-yl)phenyl]thieno[3,2-*d*]pyrimidin-4(3*H*)-one (4g)

Following procedure D with methyl 3-amino-5-(4-(morpholin-4-yl)phenyl)thiophene-2-carboxylate (0.36 g, 1.13 mmol) and *tert*-butylamine (238 μL, 2.26 mmol), the obtained crude was triturated in acetonitrile, affording 2-*tert*-butylamino-6-[4-(morpholin-4-yl)phenyl]thieno[3,2-*d*]pyrimidin-4(3*H*)-one as a pale-yellow powder (0.3 g, 69% yield). ^1^H NMR (DMSO-*d*_6_, 400 MHz) *δ* 10.41 (s, 1H), 7.64 (d, ^3^*J* = 8.4 Hz, 2H), 7.33 (s, 1H), 6.99 (d, ^3^*J* = 8.4 Hz, 2H), 6.07 (s, 1H), 3.74 (s, 4H), 3.18 (s, 4H), 1.42 (s, 9H). ^13^C NMR (DMSO-*d*_6_, 100 MHz) *δ* 160.5, 157.5, 152.2, 151.6, 150.7, 126.8 (2C), 123.4, 118.2, 114.8 (2C), 110.0, 65.9 (2C), 50.9, 47.6 (2C), 28.6 (3C). Mp > 300 °C. HRMS (ESI) *m*/*z* calculated for C_20_H_25_N_4_O_2_S [M + H]^+^ 385.1683, found 385.1689.

##### 2-*tert*-Butylamino-6-[4-(4-methylpiperazin-1-yl)phenyl]thieno[3,2-*d*]pyrimidin-4(3*H*)-one (4h)

Following procedure D with methyl-3-amino-5-(4-(4-methylpiperazin-1-yl)phenyl)thiophene-2-carboxylate (0.325 g, 0.98 mmol) and *tert*-butylamine (200 μL, 1.96 mmol), the obtained crude was purified *via* flash chromatography (using dichloromethane and methanol), affording 2-*tert*-butylamino-6-[4-(4-methylpiperazin-1-yl)phenyl]thieno[3,2-*d*]pyrimidin-4(3*H*)-one as a yellow powder (90 mg, 23% yield). ^1^H NMR (DMSO-*d*_6_, 400 MHz) *δ* 10.39 (s, 1H), 7.62 (d, ^3^*J* = 8.8 Hz, 2H), 7.31 (s, 1H), 6.97 (d, ^3^*J* = 9.2 Hz, 2H), 6.07 (s, 1H), 3.27–3.18 (m, 4H), 2.47–2.41 (m, 4H), 2.22 (s, 3H), 1.41 (s, 9H). ^13^C NMR (DMSO-*d*_6_, 100 MHz) *δ* 160.5, 157.5, 152.2, 151.4, 150.8, 126.8 (2C), 122.9, 118.1, 114.9 (2C), 109.9, 54.4 (2C), 50.9, 47.2 (2C), 45.7, 28.6 (3C). Mp = 282–284 °C. HRMS (ESI) *m*/*z* calculated for C_21_H_28_N_5_OS [M + H]^+^ 398.2009, found 398.2006.

##### 2-*tert*-Butylamino-6-[4-(piperidin-1-yl)phenyl]thieno[3,2-*d*]pyrimidin-4(3*H*)-one (4i)

Following procedure D with methyl-3-amino-5-(4-(piperidin-1-yl)phenyl)thiophene-2-carboxylate (0.350 g, 1.11 mmol) and *tert*-butylamine (232 μL, 2.21 mmol), the obtained crude was purified *via* flash chromatography (using dichloromethane and methanol), affording 2-*tert*-butylamino-6-[4-(piperidin-1-yl)phenyl]thieno[3,2-*d*]pyrimidin-4(3*H*)-one as a yellow powder (216 mg, 51% yield). ^1^H NMR (DMSO-*d*_6_, 400 MHz) *δ* 10.37 (s, 1H), 7.60 (d, ^3^*J* = 8.8 Hz, 2H), 7.28 (s, 1H), 6.95 (d, ^3^*J* = 8.8 Hz, 2H), 6.04 (s, 1H), 3.29–3.20 (m, 4H), 1.64–1.51 (m, 6H), 1.45–1.36 (m, 9H). ^13^C NMR (DMSO-*d*_6_, 100 MHz) *δ* 160.5, 157.5, 152.2, 151.7, 150.9, 126.8 (2C), 122.2, 117.8, 115.0 (2C), 109.7, 50.9, 48.5 (2C), 28.6 (3C), 24.9 (2C), 23.9. Mp = 289–291 °C. HRMS (ESI) *m*/*z* calculated for C_21_H_27_N_4_OS [M + H]^+^ 383.1900, found 383.1898.

##### 2-*tert*-Butylamino-6-[4-(methylsulfanyl)phenyl]thieno[3,2-*d*]pyrimidin-4(3*H*)-one (4j)

Following procedure D with methyl-3-amino-5-[4-(methylsulfanyl)phenyl]thiophene-2-carboxylate (0.65 g, 2.45 mmol) and *tert*-butylamine (515 μL, 4.9 mmol), the obtained crude was purified *via* flash chromatography (using dichloromethane and methanol). Fractions of interest were triturated in diethyl ether, affording 2-*tert*-butylamino-6-[4-(methylsulfanyl)phenyl]thieno[3,2-*d*]pyrimidin-4(3*H*)-one as an off-white solid (547 mg, 65% yield). ^1^H NMR (DMSO-*d*_6_, 400 MHz) *δ* 10.46 (s, 1H), 7.73 (d, ^3^*J* = 8.5 Hz, 2H), 7.48 (s, 1H), 7.32 (d, ^3^*J* = 8.5 Hz, 2H), 6.09 (s, 1H), 2.52 (s, 3H), 1.42 (s, 9H). ^13^C NMR (DMSO-*d*_6_, 100 MHz) *δ* 160.3, 157.6, 152.3, 149.6, 140.0, 129.4, 126.3 (2C), 126.1 (2C), 120.0, 111.1, 51.0, 28.6 (3C), 14.4. Mp = 272–274 °C. HRMS (ESI) *m*/*z* calculated for C_17_H_20_N_3_OS_2_ [M + H]^+^ 346.1042, found 346.1039.

##### 2-*tert*-Butylamino-6-[4-(methylsulfonyl)phenyl]thieno[3,2-*d*]pyrimidin-4(3*H*)-one (4k)

Following general procedure D with methyl-3-amino-5-[4-(methylsulfonyl)phenyl]thiophene-2-carboxylate (0.3 g, 0.96 mmol) and *tert*-butylamine (200 μL, 1.93 mmol), the obtained crude was triturated in acetonitrile, affording 2-*tert*-butylamino-6-[4-(methylsulfonyl)phenyl]thieno[3,2-*d*]pyrimidin-4(3*H*)-one as a beige powder (160 mg, 44% yield). ^1^H NMR (DMSO-*d*_6_, 400 MHz) *δ* 10.56 (s, 1H), 8.08 (d, ^3^*J* = 8.5 Hz, 2H), 7.98 (d, ^3^*J* = 8.5 Hz, 2H), 6.13 (s, 1H), 3.26 (s, 3H), 1.43 (s, 9H). ^13^C NMR (DMSO-*d*_6_, 100 MHz) *δ* 160.0, 157.6, 152.3, 147.5, 140.8, 137.6, 127.9 (2C), 126.6 (2C), 122.9, 112.9, 51.1, 43.4, 28.6 (3C). Mp = 294–296 °C. HRMS (ESI) *m*/*z* calculated for C_17_H_20_N_3_O_3_S_2_ [M + H]^+^ 378.0941, found 378.0934.

##### 4-(2-*tert*-Butylamino-4-oxo-3,4-dihydrothieno[3,2-*d*]pyrimidin-6-yl)benzenesulfonamide (4l)

Following general procedure D with methyl-3-amino-5-(4-sulfamoylphenyl)thiophene-2-carboxylate (0.2 g, 0.64 mmol) and *tert*-butylamine (135 μL, 1.28 mmol), the obtained crude was purified *via* column chromatography on silica gel (from 100% dichloromethane to 97 : 3 dichloromethane/methanol), affording 4-(2-*tert*-butylamino-4-oxo-3,4-dihydrothieno[3,2-*d*]pyrimidin-6-yl)benzenesulfonamide as a yellow powder (135 mg, 56% yield). ^1^H NMR (DMSO-*d*_6_, 400 MHz) *δ* 10.55 (s, 1H), 8.00 (d, ^3^*J* = 8.5 Hz, 2H), 7.87 (d, ^3^*J* = 8.5 Hz, 2H), 7.67 (s, 1H), 7.44 (s, 2H), 6.14 (s, 1H), 1.42 (s, 9H). ^13^C NMR (DMSO-*d*_6_, 100 MHz) *δ* 160.0, 157.6, 152.3, 147.9, 144.2, 136.0126.5 (2C), 126.3 (2C), 122.3, 112.5, 51.0, 28.6 (3C). Mp > 300 °C. HRMS (ESI) *m*/*z* calculated for C_16_H_19_N_4_O_3_S_2_ [M + H]^+^ 379.0893, found 379.0893.

##### 2-*tert*-Butylamino-6-[4-(morpholin-4-ylsulfonyl)phenyl]thieno[3,2-*d*]pyrimidin-4(3*H*)-one (4m)

Following procedure D with methyl-3-amino-5-[4-(morpholin-4-ylsulfonyl)phenyl]thiophene-2-carboxylate (0.3 g, 0.78 mmol) and *tert*-butylamine (170 μL, 1.57 mmol), the obtained crude was purified *via* flash chromatography (using dichloromethane and methanol). Fractions of interest were triturated in diethyl ether, affording 2-*tert*-butylamino-6-[4-(morpholin-4-ylsulfonyl)phenyl]thieno[3,2-*d*]pyrimidin-4(3*H*)-one as a yellow powder (117 mg, 33% yield). ^1^H NMR (DMSO-*d*_6_, 400 MHz) *δ* 10.57 (s, 1H), 8.09 (d, ^3^*J* = 8.5 Hz, 2H), 7.80 (d, ^3^*J* = 8.5 Hz, 2H), 7.74 (s, 1H), 6.14 (s, 1H), 3.68–3.61 (m, 4H), 2.96–2.87 (m, 4H), 1.43 (s, 9H). ^13^C NMR (DMSO-*d*_6_, 100 MHz) *δ* 160.0, 157.6, 152.3, 147.4, 137.4, 134.5, 128.6 (2C), 126.7 (2C), 122.8, 112.9, 62.3 (2C), 51.1, 45.9 (2C), 28.6 (3C). Mp > 300 °C. HRMS (ESI) *m*/*z* calculated for C_20_H_25_N_4_O_4_S_2_ [M + H]^+^ 449.1312, found 449.1305.

##### 2-*tert*-Butylamino-6-[2-(morpholin-4-yl)pyrimidin-5-yl]thieno[3,2-*d*]pyrimidin-4(3*H*)-one (4n)

Following procedure D with methyl-3-amino-5-(2-(morpholin-4-yl)pyrimidin-5-yl)thiophene-2-carboxylate (0.2 g, 0.62 mmol) and *tert*-butylamine (131 μL, 1.25 mmol), the obtained crude was purified *via* flash chromatography (using dichloromethane and methanol), affording 2-*tert*-butylamino-6-[2-(morpholin-4-yl)pyrimidin-5-yl]thieno[3,2-*d*]pyrimidin-4(3*H*)-one as a yellow powder (138 mg, 57% yield). ^1^H NMR (DMSO-*d*_6_, 400 MHz) *δ* 10.43 (s, 1H), 8.80 (s, 2H), 6.09 (s, 1H), 3.81–3.74 (m, 4H), 3.70–3.64 (m, 4H), 1.41 (s, 9H). ^13^C NMR (DMSO-*d*_6_, 100 MHz) *δ* 160.7, 160.2, 157.4, 155.2 (2C), 152.3, 144.7, 119.1, 116.5, 110.4, 65.9 (2C), 51.0, 44.0 (2C), 28.6 (3C). Mp > 300 °C. HRMS (ESI) *m*/*z* calculated for C_18_H_23_N_6_O_2_S [M + H]^+^ 387.1598, found 387.1597.

##### 2-*tert*-Butylamino-6-[2-(4-methylpiperazin-1-yl)pyrimidin-5-yl]thieno[3,2-*d*]pyrimidin-4(3*H*)-one (4o)

Following procedure D with methyl-3-amino-5-(2-(4-methylpiperazin-1-yl)pyrimidin-5-yl)thiophene-2-carboxylate (0.195 g, 0.58 mmol) and *tert*-butylamine (122 μL, 1.17 mmol), the obtained crude was purified *via* flash chromatography (using dichloromethane and methanol), affording 2-*tert*-butylamino-6-[2-(4-methylpiperazin-1-yl)pyrimidin-5-yl]thieno[3,2-*d*]pyrimidin-4(3*H*)-one as a beige powder (45 mg, 19% yield). ^1^H NMR (DMSO-*d*_6_, 400 MHz) *δ* 10.43 (s, 1H), 8.77 (s, 2H), 7.43 (s, 1H), 6.09 (s, 1H), 3.84–3.75 (m, 4H), 2.41–2.32 (m, 4H), 2.21 (s, 3H), 1.41 (s, 9H). ^13^C NMR (DMSO-*d*_6_, 100 MHz) *δ* 160.6, 160.3, 157.4, 155.2 (2C), 152.3, 144.8, 119.0, 116.1, 110.3, 54.3 (2C), 51.0, 45.8, 43.4 (2C), 28.6 (3C). Mp = 289–291. HRMS (ESI) *m*/*z* calculated for C_19_H_26_N_7_OS [M + H]^+^ 400.1914, found 400.1914.

#### 4.1.8. Reduction of nitro derivatives

##### 6-(4-aminophenyl)-2-*tert*-butylaminothieno[3,2-*d*]pyrimidin-4(3*H*)-one (4p)

To a solution of 2-(tert-butylamino)-6-(4-nitrophenyl)thieno[3,2-*d*]pyrimidin-4(3*H*)-one (0.227 g, 0.66 mmol) in acetic acid (6.9 mL), iron powder (0.368 g, 6.59 mmol) was added and the mixture was stirred at 80 °C for 15 min. Excess solvent was removed *in vacuo* and the crude product was purified *via* column chromatography on silica gel (starting from 100% dichloromethane to 95 : 5 dichloromethane/methanol). Fractions of interest were purified *via* flash chromatography (using dichloromethane/methanol), affording 6-(4-aminophenyl)-2-*tert*-butylaminothieno[3,2-*d*]pyrimidin-4(3*H*)-one as a beige solid (66 mg, 32% yield). ^1^H NMR (DMSO-*d*_6_, 400 MHz) *δ* 10.31 (s, 1H), 7.45 (d, ^3^*J* = 8.4 Hz, 2H), 7.16 (s, 1H), 6.60 (d, ^3^*J* = 8.4 Hz, 2H), 6.01 (s, 1H), 5.56 (s, 2H), 1.41 (s, 9H). ^13^C NMR (DMSO-*d*_6_, 100 MHz) *δ* 160.5, 157.4, 152.1, 151.9, 150.2, 126.9 (2C), 120.3, 116.7, 113.8 (2C), 109.0, 50.9, 28.6 (3C). Mp = 259–261 °C. HRMS (ESI) *m*/*z* calculated for C_16_H_19_N_4_OS [M + H]^+^ 315.1274, found 315.1274.

##### 6-(4-aminophenyl)-2-(propan-2-ylamino)thieno[3,2-*d*]pyrimidin-4(3*H*)-one (4q)

To a solution of 6-(4-nitrophenyl)-2-(propan-2-ylamino)thieno[3,2-*d*]pyrimidin-4(3*H*)-one (0.156 g, 0.47 mmol) in acetic acid (4.7 mL), iron powder (0.264 g, 4.72 mmol) was added and the mixture was stirred at 60 °C for 20 min. The reaction mixture was filtered through celite, the filtrate was isolated, and the excess solvent was removed *in vacuo*. The obtained crude was purified *via* column chromatography on silica gel (starting from 100% dichloromethane to 90 : 10 dichloromethane/isopropanol). Fractions of interest were recrystallized in isopropanol, affording 6-(4-aminophenyl)-2-(propan-2-ylamino)thieno[3,2-*d*]pyrimidin-4(3*H*)-one as light brown powder (30 mg, 21% yield). ^1^H NMR (DMSO-*d*_6_, 400 MHz) *δ* 10.47 (s, 1H), 7.44 (d, ^3^*J* = 8.6 Hz, 2H), 7.18 (s, 1H), 6.60 (d, ^3^*J* = 8.6 Hz, 2H), 6.07 (d, ^3^*J* = 7.3 Hz, 1H), 5.56 (s, 2H), 4.08–3.94 (m, 1H), 1.17 (d, ^3^*J* = 6.55 Hz, 6H). ^13^C NMR (DMSO-*d*_6_, 100 MHz) *δ* 160.8, 157.5, 152.6, 152.0, 150.2, 126.9 (2C), 120.3, 116.4, 113.8 (2C), 109.0, 42.0, 22.4 (2C). Mp = 296–298 °C. HRMS (ESI) *m*/*z* calculated for C_15_H_17_N_4_OS [M + H]^+^ 301.1118, found 301.1117.

##### *N*-[4-(2-*tert*-butylamino-4-oxo-3,4-dihydrothieno[3,2-*d*]pyrimidin-6-yl)phenyl]acetamide (4r)

Isolated as a side product from the purification of 6-(4-aminophenyl)-2-(*tert*-butylamino)thieno[3,2-*d*]pyrimidin-4(3*H*)-one when the reaction was stirred at 120 °C for 30 min (brown powder). ^1^H NMR (DMSO-*d*_6_, 400 MHz) *δ* 10.43 (bs, 1H), 10.12 (s, 1H), 7.73 (d, ^3^*J* = 11.6 Hz, 2H), 7.66 (d, ^3^*J* = 12 Hz, 2H), 7.41 (s, 1H), 6.07 (s, 1H), 2.07 (s, 3H), 1.42 (s, 9H). ^13^C NMR (DMSO-*d*_6_, 100 MHz) *δ* 168.5, 160.3, 157.5, 152.2, 150.0, 140.3, 127.5, 126.4 (2C), 119.5, 119.2 (2C), 110.8, 51.0, 28.6 (3C), 24.1. Mp > 300 °C HRMS (ESI) *m*/*z* calculated for C_18_H_21_N_4_O_2_S [M + H]^+^ 357.1380, found 357.1377.

#### 4.1.9. 2-*tert*-Butylamino-6-[4-(methylsulfonimidoyl)phenyl]thieno[3,2-*d*]pyrimidin-4(3*H*)-one (4s)

To a suspension of 2-*tert*-butylamino-6-[4-(methylsulfanyl)phenyl]thieno[3,2-*d*]pyrimidin-4(3*H*)-one (0.15 g, 0.43 mmol) and ammonium carbamate (51 mg, 0.65 mmol) in methanol (868 μL), (diacetoxyiodo)benzene (0.293 g, 0.91 mmol) was added and the reaction mixture was stirred for 30 min at room temperature. The excess solvent was removed *in vacuo* and the obtained crude was purified *via* column chromatography on neutral alumina gel (starting from 100% dichloromethane to 95 : 5 dichloromethane/methanol), affording 2-*tert*-butylamino-6-[4-(methylsulfonimidoyl)phenyl]thieno[3,2-*d*]pyrimidin-4(3*H*)-one as a yellow powder (80 mg, 49% yield). ^1^H NMR (DMSO-*d*_6_, 400 MHz) *δ* 10.55 (bs, 1H), 8.05–7.94 (m, 4H), 7.69 (s, 1H), 6.13 (s, 1H), 4.30 (s, 1H), 3.10 (s, 3H), 1.43 (s, 9H). ^13^C NMR (DMSO-*d*_6_, 100 MHz) *δ* 160.1, 157.6, 152.3, 147.9, 144.3, 136.6, 128.2 (2C), 126.3 (2C), 122.4, 112.7, 51.0, 45.6, 28.6 (3C). Mp = 251–253 °C. HRMS (ESI) *m*/*z* calculated for C_17_H_21_N_4_O_2_S_2_ [M + H]^+^ 377.1100, found 377.1094.

#### 4.1.10. *c* log *D*_7.4_ calculations

*c* log *D*_7.4_ were calculated for all compounds using MarvinSketch software (developed by ChemAxon). The following calculations options were used: consensus (log *P* method), 0.1 mol dm^−1^^3^ Cl^−^ concentration, and 0.1 mol dm^−1^^3^ Na^+^ K^+^ concentration.

### 4.2. Biology

#### 4.2.1. Antiplasmodial evaluation

In this study, the K_1_ culture-adapted *P. falciparum* strain resistant to chloroquine, pyrimethamine, and proguanil was used in an *in vitro* culture. It was maintained in continuous culture, as described previously by Trager and Jensen.^18^ Cultures were maintained in fresh A^+^ human erythrocytes at 2.5% hematocrit in complete medium (RPMI 1640 with 25 mM HEPES, 25 mM NaHCO_3_, 10% of A^+^ human serum) at 37 °C under reduced O_2_ atmosphere (gas mixture 10% O_2_, 5% CO_2_, and 85% N_2_). Parasitemia was maintained daily between 1 and 3%. The *P. falciparum* drug susceptibility test was carried out by comparing quantities of DNA in treated and control cultures of parasite in human erythrocytes according to a SYBR Green I fluorescence-based method^19^ using a 96-well fluorescence plate reader. Compounds previously dissolved in DMSO (final concentration less than 0.5% v/v) were incubated in a total assay volume of 200 μL (RPMI, 2% hematocrit and 0.4% parasitemia) for 72 h in a humidified atmosphere (10% O_2_ and 5% CO_2_) at 37 °C in 96-well flat bottom plates. Duplicate assays were performed for each sample. After incubation, plates were frozen at 20 °C for 24 h. Then, the frozen plates were thawed for 1 h at 37 °C. Fifteen μL of each sample were transferred to 96-well flat bottom non-sterile black plates (Greiner Bio-one, Kremsmünster, Austria), already containing 15 μL of the SYBR Green I lysis buffer (2X SYBR Green I, 20 mM Tris base pH 7.5, 20 mM EDTA, 0.008% w/v saponin, 0.08% w/v Triton X-100). Negative control treated by solvents (DMSO or H_2_O) and positive controls (chloroquine and doxycycline) were added to each set of experiments. Plates were incubated for 15 min at 37 °C and then read on a TECAN Infinite F-200 spectrophotometer with excitation and emission wavelengths at 485 and 535 nm, respectively. The concentrations of compounds required to induce a 50% decrease in parasite growth (EC_50_ K_1_) were calculated from three independent experiments.

#### 4.2.2. Cytotoxic evaluation

The HepG2 cell line was maintained at 37 °C, 5% CO_2_, at 90% humidity in MEM supplemented with 10% fetal bovine serum, 1% l-glutamine (200 mM), and penicillin (100 U per mL)/streptomycin (100 μg mL^−1^) (complete RPMI medium). The cytotoxicity of the tested molecules on the HepG2 (hepatocarcinoma cell line purchased from ATCC, ref HB-8065) cell line was assessed according to the method of Mosmann^20^ with slight modifications. Briefly, 5 × 10^3^ cells in 100 μL of complete medium were inoculated into each well of 96-well plates and incubated at 37 °C in humidified 5% CO_2_. After 24 h incubation, 100 μL of medium with various product concentrations dissolved in DMSO (final concentration less than 0.5% v/v) were added and the plates were incubated for 72 h at 37 °C. Triplicate assays were performed for each sample. Each plate was then microscopically examined for possible precipitate formation before the medium was aspirated from the wells. Next, 100 μL of MTT (3-(4,5-dimethyl-2-thiazolyl)-2,5-diphenyl-2H-tetrazolium bromide) solution (0.5 mg mL^−1^ in medium without FBS) was added to each well. Cells were incubated for 2 h at 37 °C. After this time, the MTT solution was removed and DMSO (100 μL) was added to dissolve the resulting blue formazan crystals. Plates were shaken vigorously (700 rpm) for 10 min. The absorbance was measured at 570 nm with 630 nm as a reference wavelength using a BIO-TEK ELx808 Absorbance Microplate Reader (LabX, Midland, ON, Canada). DMSO was used as a blank and doxorubicin (purchased from Sigma Aldrich) as a positive control. Cell viability was calculated as a percentage of control (cells incubated without the compound). The 50% cytotoxic concentration (CC_50_) was determined from the dose–response curve using TableCurve software 2D v.5.0. CC_50_ values to represent the mean value calculated from three independent experiments.

#### 4.2.3. Microsomal stability

The tested product and propranolol, used as a reference, were incubated in duplicate (reaction mixture volume of 0.5 mL) with female mouse microsomes (CD-1, 20 mg mL; BD Gentest) at 37 °C in a 50 mM phosphate buffer, pH 7.4, in the presence of MgCl_2_ (5 mM), NADP (1 mM), glucose-6-phosphate dehydrogenase (G6PD) (0.4 U per mL), and glucose-6-phosphate (5 mM). For the estimation of the intrinsic clearance, 50 μL aliquots were collected at 0, 5, 10, 20, 30, and 40 min and the reaction was stopped with 4 volumes of acetonitrile (ACN) containing the internal standard. After centrifugation at 10 000×*g* for 10 min at 4 °C, the supernatants were conserved at 4 °C for immediate analysis or placed at −80 °C in case of postponement of the analysis. Controls (time zero and final time point) in triplicate were prepared by the incubation of the internal standard with microsomes denatured by acetonitrile. The LC-MS system used for this study was a Waters Acquity I-Class/Xevo TQD equipped with a Waters Acquity BEH C_18_ column, 50 by 2.1 mm, 1.7 μm. The mobile phases were 10 mM ammonium acetate (mobile phase A) and acetonitrile with 0.1% formic acid (mobile phase B). The injection volume was 1 μL, and the flow rate was 600 μL min^−1^. Chromatographic analysis, with a total duration of 4 min, was made with the following gradient: 0 < *t* < 0.2 min, 2% mobile phase B; 0.2 < *t* < 2 min, linear increase to 98% mobile phase B; 2 < *t* < 2.5 min, 98% mobile phase B; 2.5 < *t* < 2.6 min, linear decrease to 2% mobile phase B; 2.6 < *t* < 4 min, 2% mobile phase B. The quantification of each compound was obtained by converting the average of the ratios of the analyte/internal standard surfaces to the percentage of consumed product. The ratio of the control at t0 corresponded to 0% of the product consumed. The calculation of the half-life (*t*_1/2_) of each compound in the presence of microsomes was carried out according to the equation *t*_1/2_ = (ln_2_)/*k*, where *k* is the first-order degradation constant (the slope of the logarithm of the compound concentration *versus* incubation time). The intrinsic clearance *in vitro* (Cl_int_, expressed in mlmin^−1^mg) was calculated according to the equation Cl_int_ = (dose/AUC_∞_)/[microsomes], where dose is the initial concentration of the product in the sample, AUC_∞_ is the area under the concentration–time curve extrapolated to infinity, and [microsomes] is the microsome concentration expressed in mg μL^−1^.

## Author contributions

R. M.: conceptualization, investigation, visualization, writing – original draft, writing – review and editing; P. L.: investigation, writing – review and editing; S. H.: formal analysis, investigation, validation; C. D.: investigation, writing – review and editing; F. S.: investigation; D. A.: investigation; N. M.: supervision, writing – review and editing; N. A.: resources, supervision, validation; V. L.: resources, supervision, funding acquisition; P. V.: supervision, writing – review and editing; D. M.: funding acquisition; P. V.: resources, supervision, writing – review and editing; N. P.: conceptualization, funding acquisition, project administration, resources, supervision, validation, writing – review and editing.

## Conflicts of interest

There are no conflicts to declare.

## Supplementary Material

RA-012-D2RA01687G-s001
