# Supplementary material for: Pd-catalyzed C–C and C–N cross-coupling reactions in 2-aminothieno[3,2-d]pyrimidin-4(3H)-one series for antiplasmodial pharmacomodulation
Source: RSC Adv. 2022 Jul 8;12(31):20004–21. doi: 10.1039/d2ra01687g (PMC9264115; doi:10.1039/d2ra01687g)
Supplement: RA-012-D2RA01687G-s001 [file RA-012-D2RA01687G-s001.pdf]

Electronic Supplementary Material (ESI) for RSC Advances.
This journal is © The Royal Society of Chemistry 2022

Supplementary information for

Pd-catalyzed C-C and C-N cross-coupling reactions in 2-aminothieno[3,2-*d*]pyrimidin-4(3*H*)-one series for antiplasmodial pharmacomodulation

Romain Mustière,[a] Prisca Lagardère, [b] Sébastien Hutter,[c] Céline Deraeve,[d] Florian Schwalen,[d] Dyhia Amrane,[a] Nicolas Masurier,[b] Nadine Azas,[c] Vincent Lisowski,[b] Pierre Verhaeghe,[d,e] Dominique Mazier,[f] Patrice Vanelle [a,g] and Nicolas Primas [a,g,*]

a) Aix Marseille Université, CNRS, ICR UMR 7273, Equipe Pharmaco-Chimie Radicalaire, Faculté de Pharmacie, Marseille, France.
b) Institut des Biomolécules Max Mousseron, UMR 5247, CNRS, Université de Montpellier, ENSCM, UFR des Sciences Pharmaceutiques et Biologiques, Montpellier, France.
c) Aix Marseille Université, IRD, AP-HM, SSA, VITROME, Marseille, France.
d) LCC-CNRS, Université de Toulouse, CNRS UPR 8241, UPS, Toulouse, France.
e) CHU de Nîmes, service de pharmacie, Nimes, France.
f) Centre d'Immunologie et des Maladies Infectieuses (CIMI), INSERM, CNRS, Sorbonne Université, Paris, France.
g) Service Central de la Qualité et de l'Information Pharmaceutiques, AP-HM, Hôpital Conception, Marseille, France.

Table of contents

Suggested mechanism of the cyclization of thiophene aminoesters into thienopyrimidinones…...……2

^{1}H and ^{13}C NMR spectra for all biologically tested compounds………………………………………………………………………………………………3

Suggested mechanism of the cyclization of thiophene aminoesters into thienopyrimidinones (Table 7 of the article)

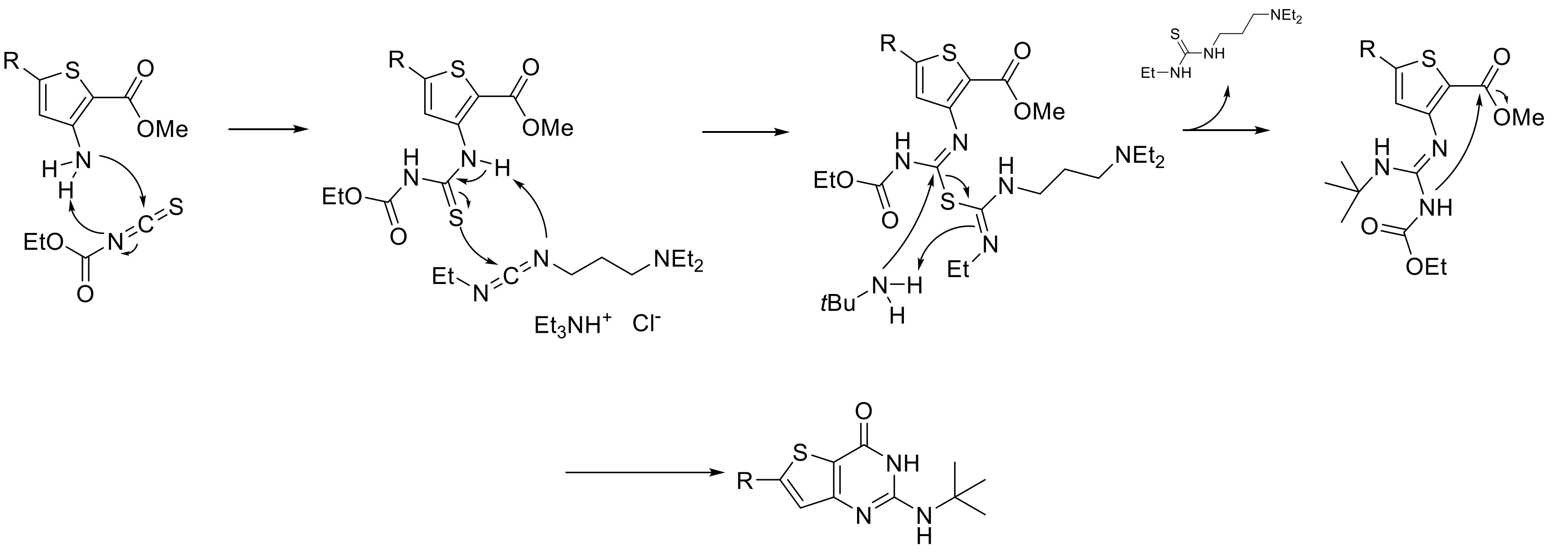

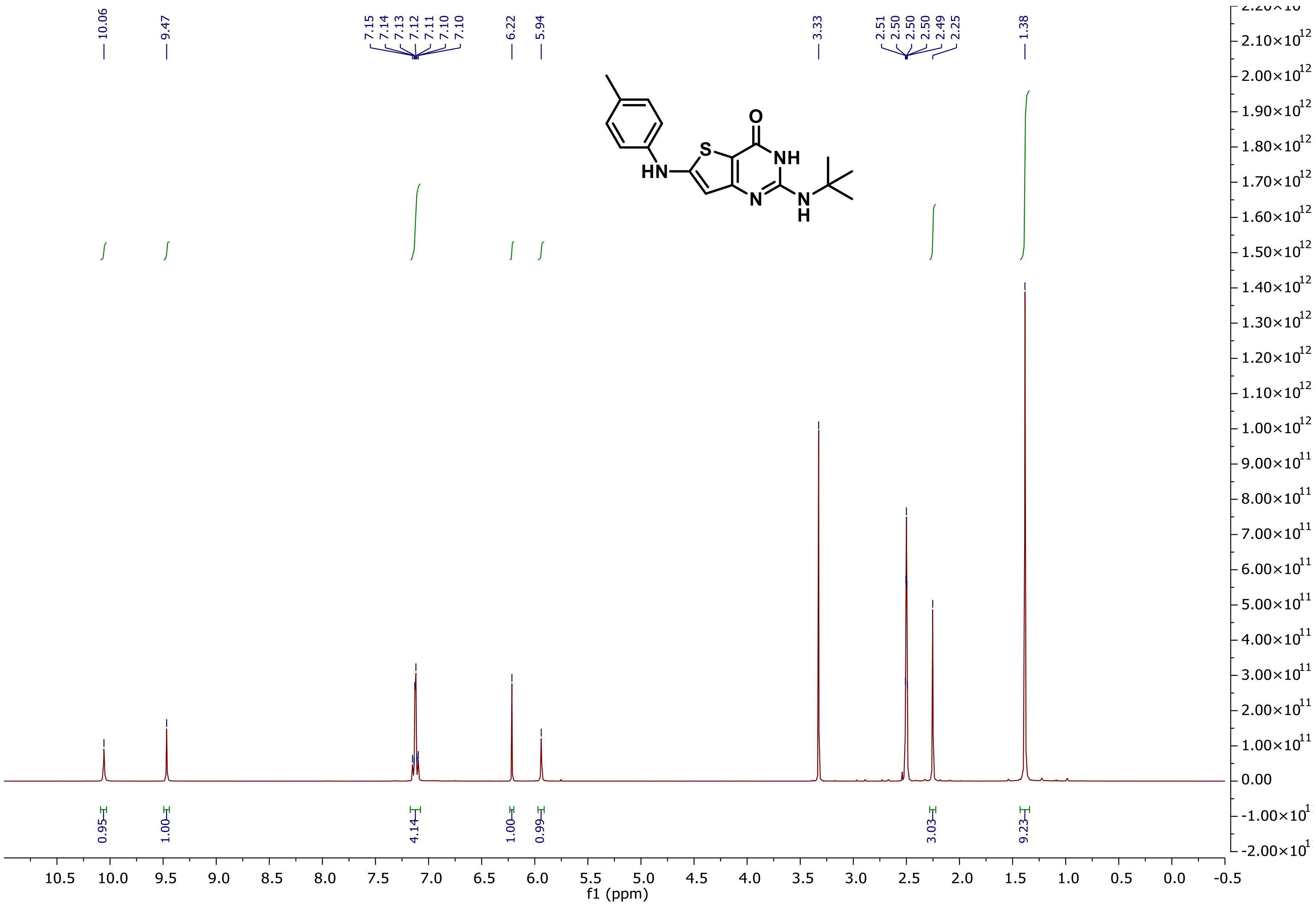
10.06
9.47
7.15
7.14
7.13
7.12
7.11
7.10
7.10
6.22
5.94
3.33
2.51
2.50
2.50
2.50
2.49
2.25
1.38
O
NH
S
HN
N
N
H
0.95
1.00
4.14
1.00
0.99
3.03
9.23
10.5 10.0 9.5 9.0 8.5 8.0 7.5 7.0 6.5 6.0 5.5 5.0 4.5 4.0 3.5 3.0 2.5 2.0 1.5 1.0 0.5 0.0 -0.5
f1 (ppm)

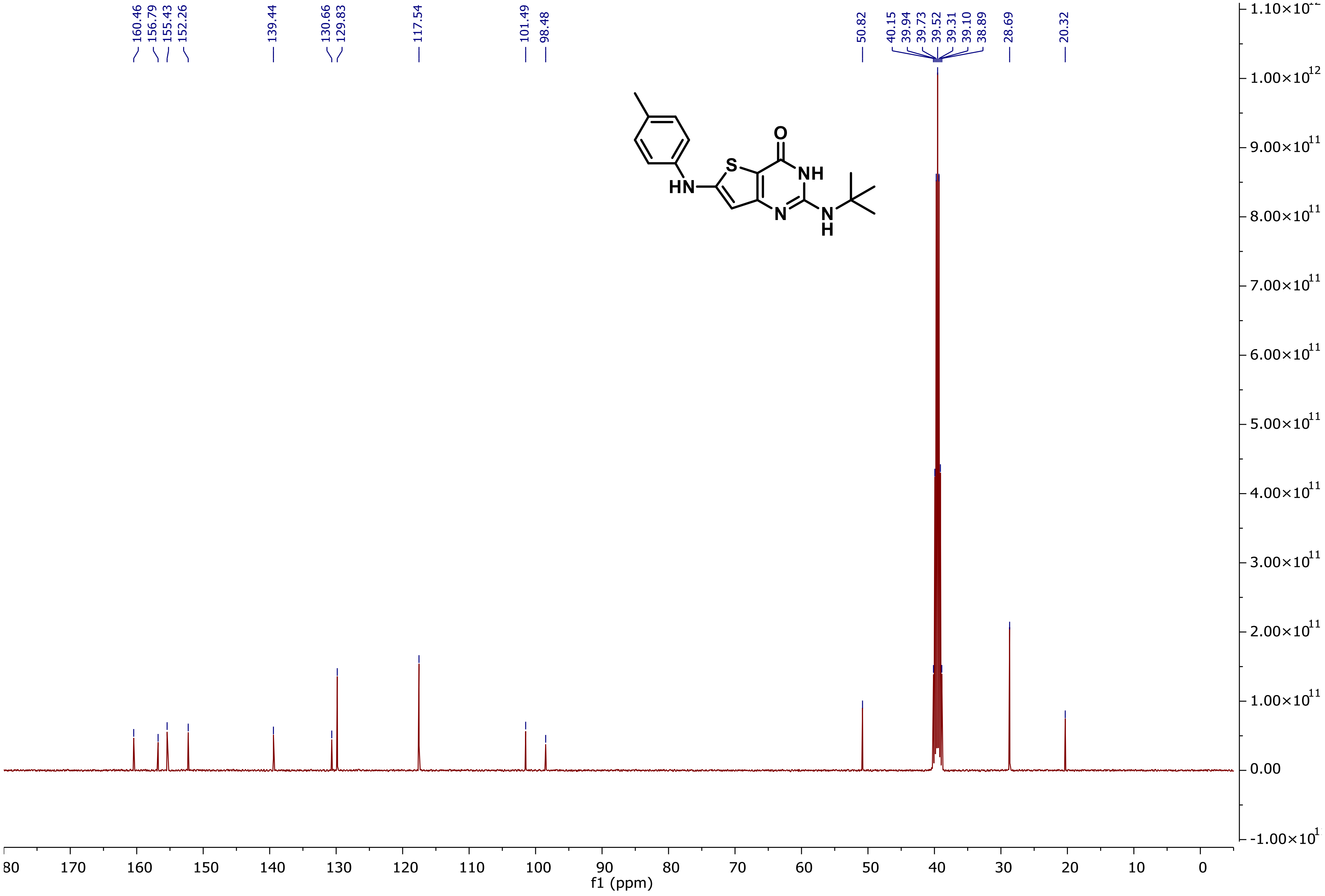
160.46
156.79
155.43
152.26
139.44
130.66
129.83
117.54
101.49
98.48
50.82
40.15
39.94
39.73
39.52
39.31
39.10
38.89
28.69
20.32
O
NH
S
HN
N
N
H
f1 (ppm)

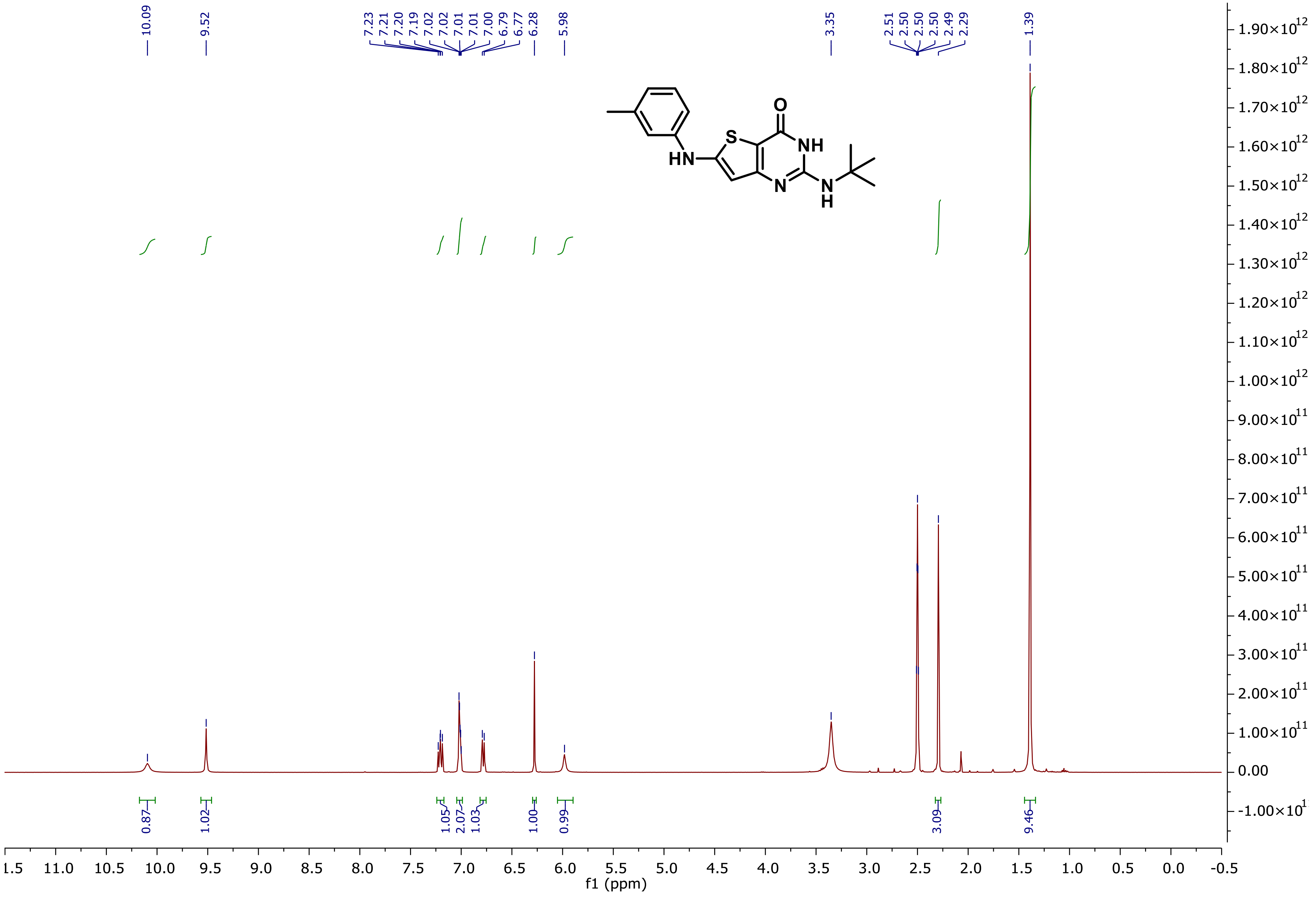
10.09
9.52
7.23
7.21
7.20
7.19
7.02
7.02
7.01
7.01
7.00
6.79
6.77
6.28
5.98
3.35
2.51
2.50
2.50
2.50
2.49
2.29
1.39
O
NH
S
HN
N
N
H
0.87
1.02
1.05
2.07
1.03
1.00
0.99
3.09
9.46
f1 (ppm)

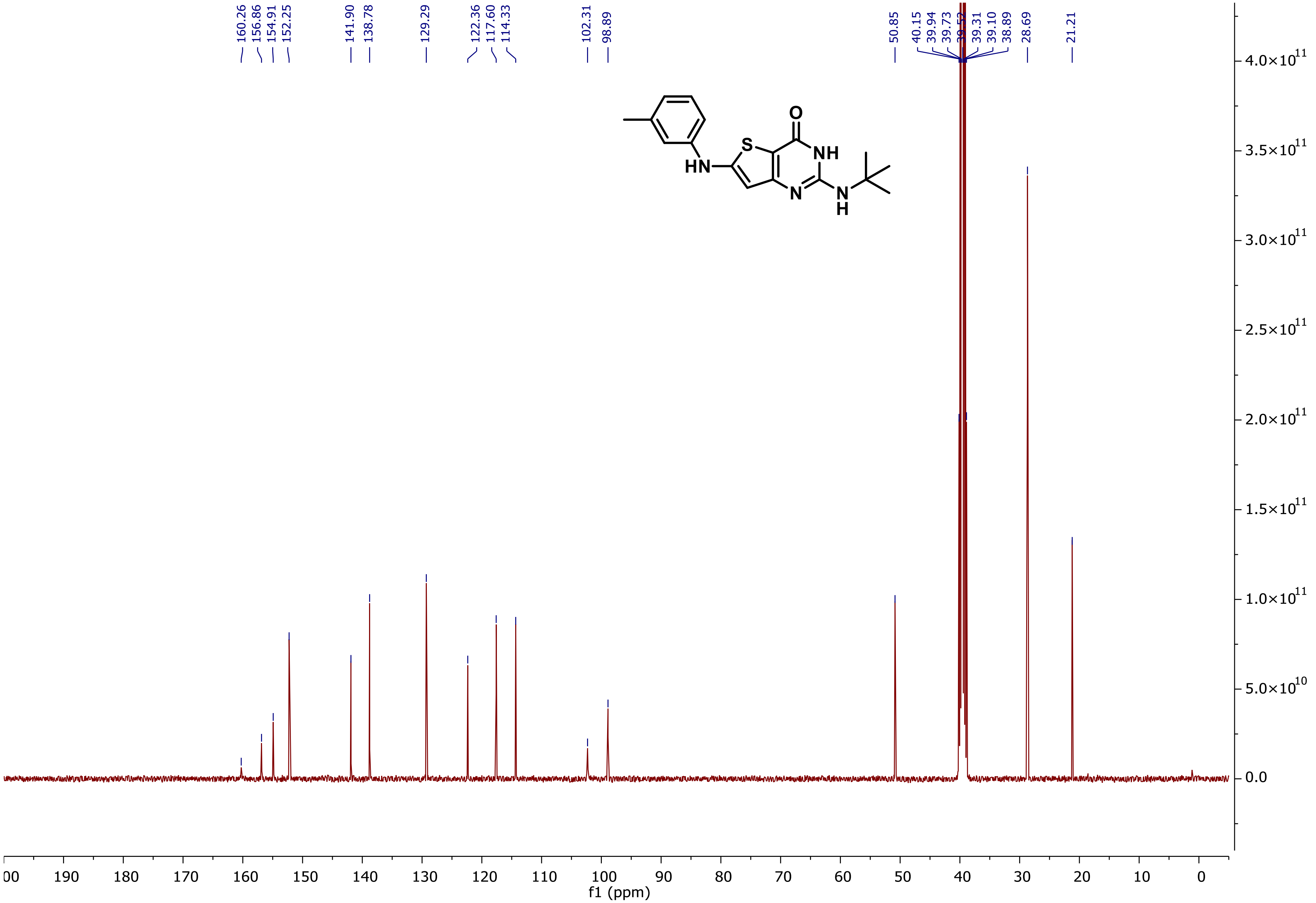
160.26
156.86
154.91
152.25
141.90
138.78
129.29
122.36
117.60
114.33
102.31
98.89
50.85
40.15
39.94
39.73
39.52
39.31
39.10
38.89
28.69
21.21
O
NH
S
HN
N
N
H
4.0×10^11
3.5×10^11
3.0×10^11
2.5×10^11
2.0×10^11
1.5×10^11
1.0×10^11
5.0×10^10
0.0
00
190
180
170
160
150
140
130
120
110
100
90
80
70
60
50
40
30
20
10
0
f1 (ppm)

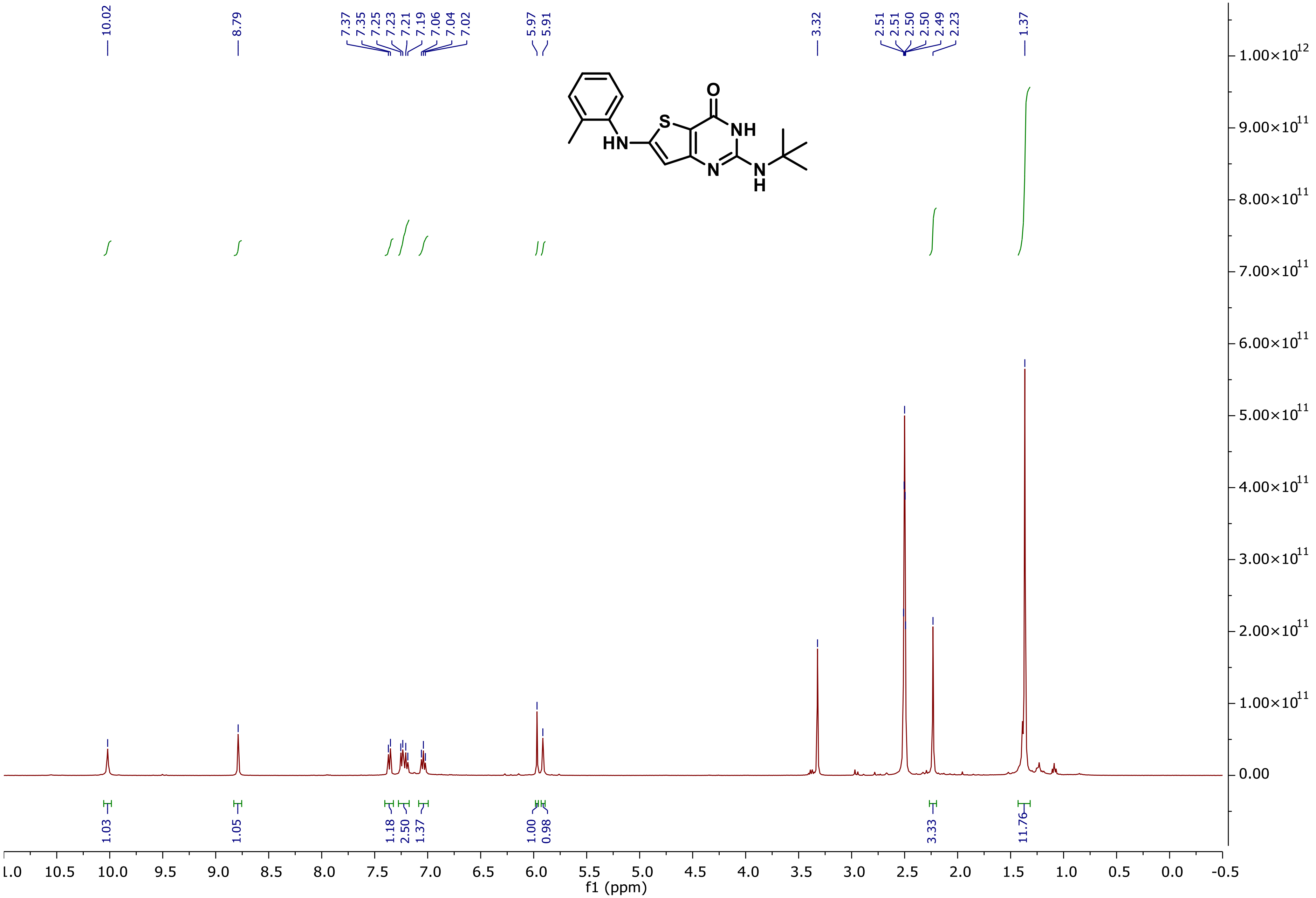
10.02
8.79
7.37
7.35
7.25
7.23
7.21
7.19
7.06
7.04
7.02
5.97
5.91
3.32
2.51
2.51
2.50
2.50
2.49
2.23
1.37
1.03
1.05
1.18
2.50
1.37
1.00
0.98
3.33
11.76
f1 (ppm)

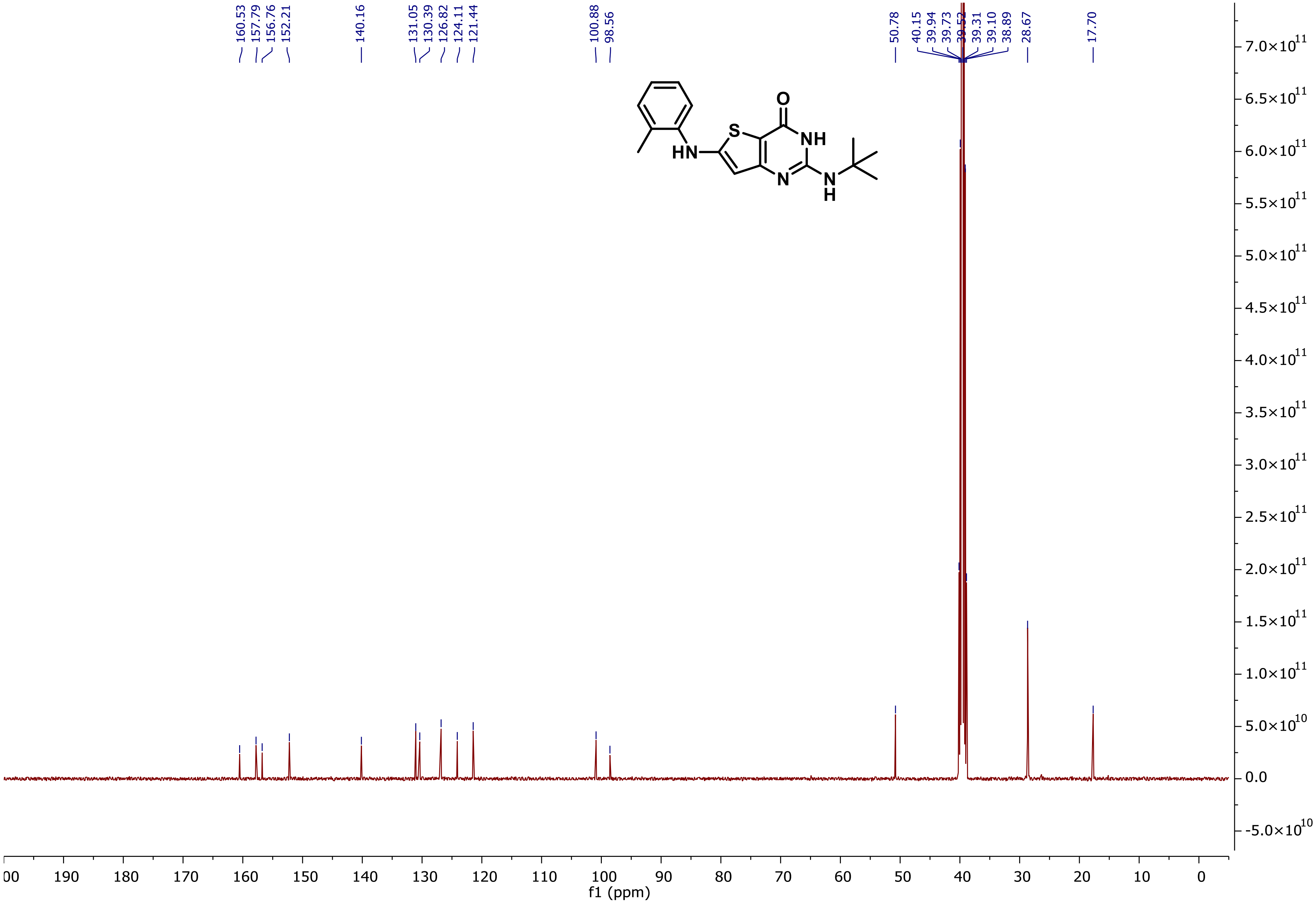
160.53
157.79
156.76
152.21
140.16
131.05
130.39
126.82
124.11
121.44
100.88
98.56
50.78
40.15
39.94
39.73
39.52
39.31
39.10
38.89
28.67
17.70
f1 (ppm)

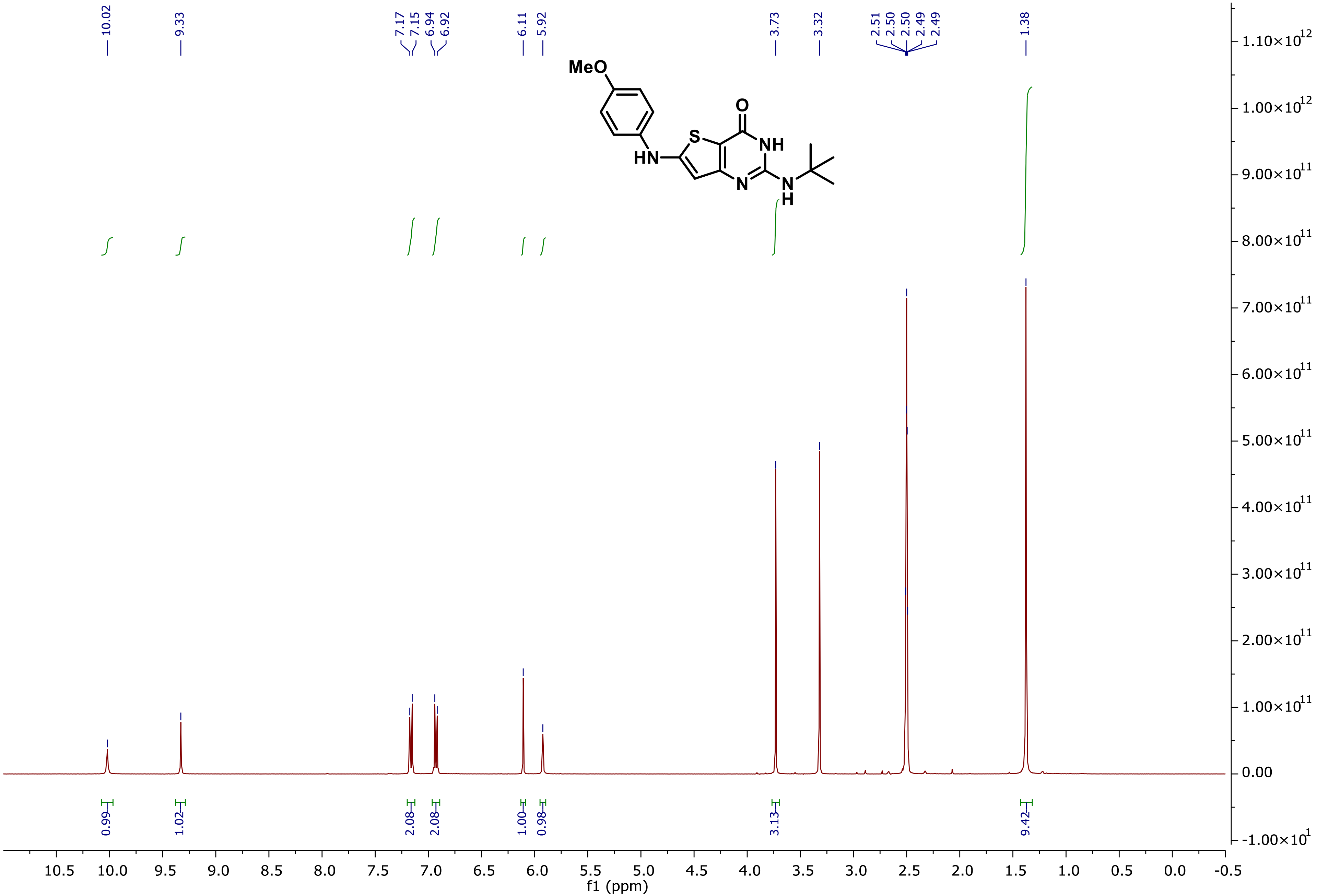

10.02
9.33
7.17
7.15
6.94
6.92
6.11
5.92
3.73
3.32
2.51
2.50
2.50
2.49
2.49
1.38
MeO
O
S
NH
HN
N
N
H
0.99
1.02
2.08
2.08
1.00
0.98
3.13
9.42
10.5
10.0
9.5
9.0
8.5
8.0
7.5
7.0
6.5
6.0
5.5
5.0
4.5
4.0
3.5
3.0
2.5
2.0
1.5
1.0
0.5
0.0
-0.5
f1 (ppm)
1.10×10^12
1.00×10^12
9.00×10^11
8.00×10^11
7.00×10^11
6.00×10^11
5.00×10^11
4.00×10^11
3.00×10^11
2.00×10^11
1.00×10^11
0.00
-1.00×10^1

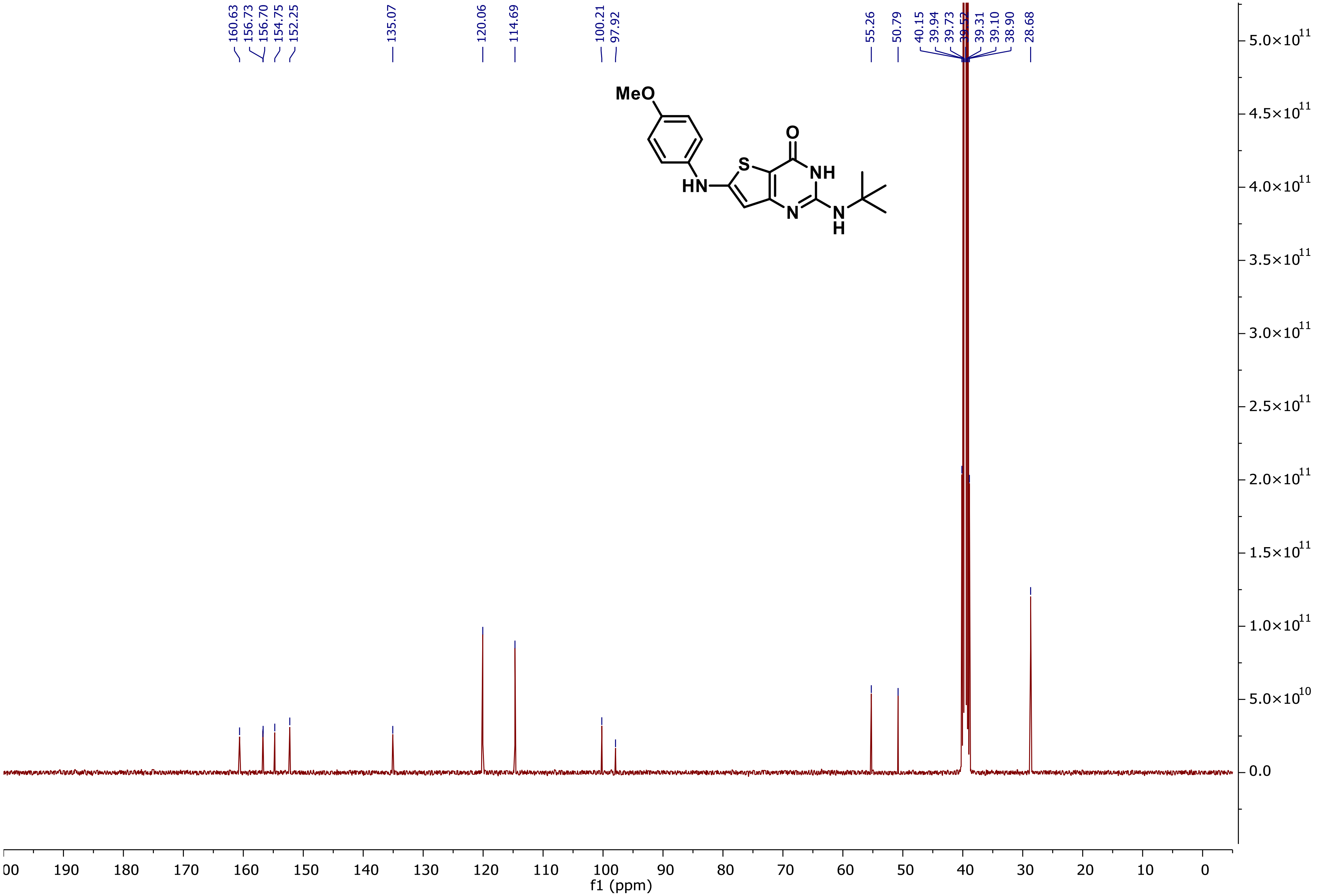
160.63
156.73
156.70
154.75
152.25
135.07
120.06
114.69
100.21
97.92
55.26
50.79
40.15
39.94
39.73
39.31
39.10
38.90
28.68
MeO
S
O
NH
HN
N
N
H
f1 (ppm)

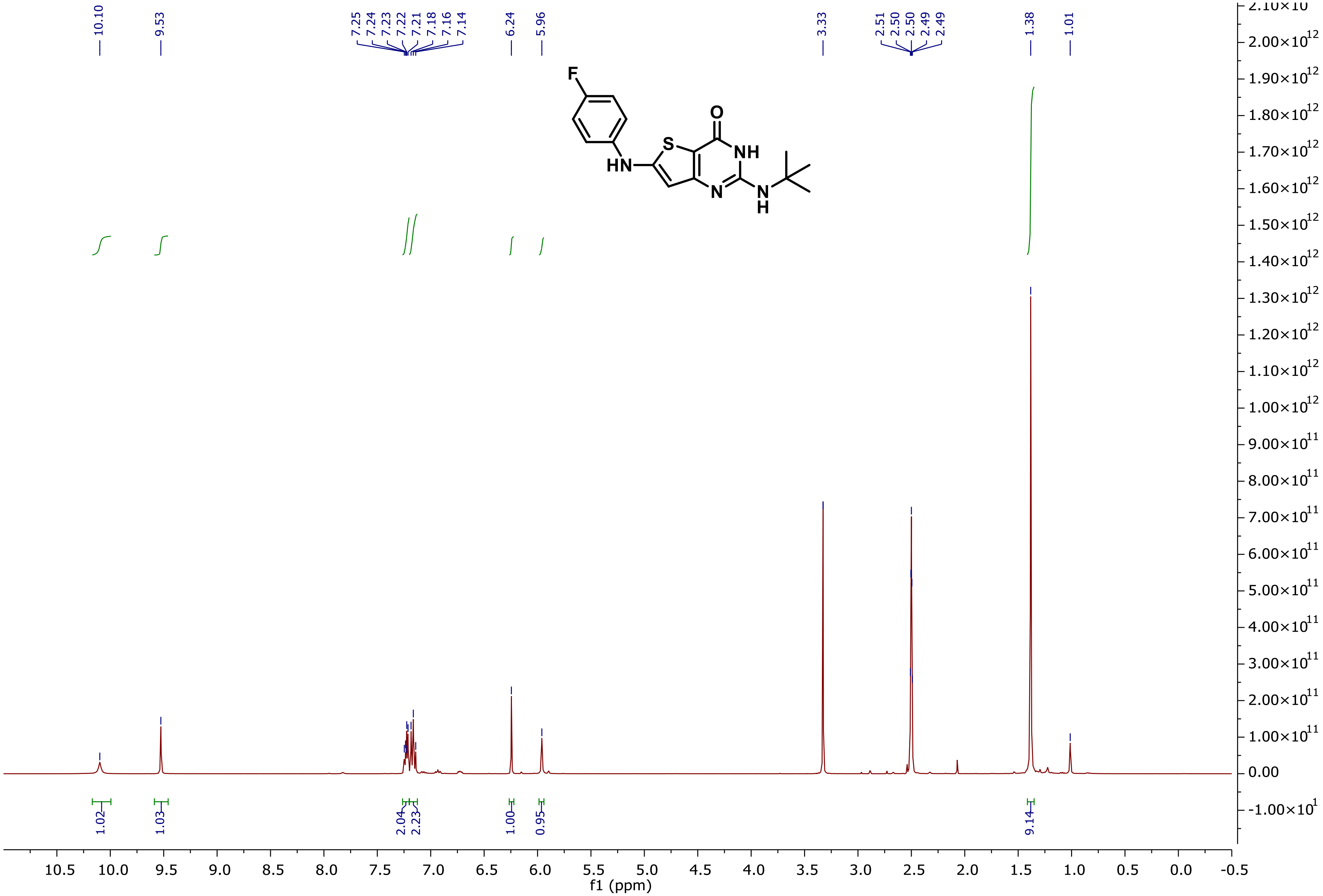

10.10
9.53
7.25
7.24
7.23
7.22
7.21
7.18
7.16
7.14
6.24
5.96
3.33
2.51
2.50
2.50
2.49
2.49
1.38
1.01
F
O
S
NH
HN
N
N
H
1.02
1.03
2.04
2.23
1.00
0.95
9.14
10.5
10.0
9.5
9.0
8.5
8.0
7.5
7.0
6.5
6.0
5.5
5.0
4.5
4.0
3.5
3.0
2.5
2.0
1.5
1.0
0.5
0.0
-0.5
f1 (ppm)

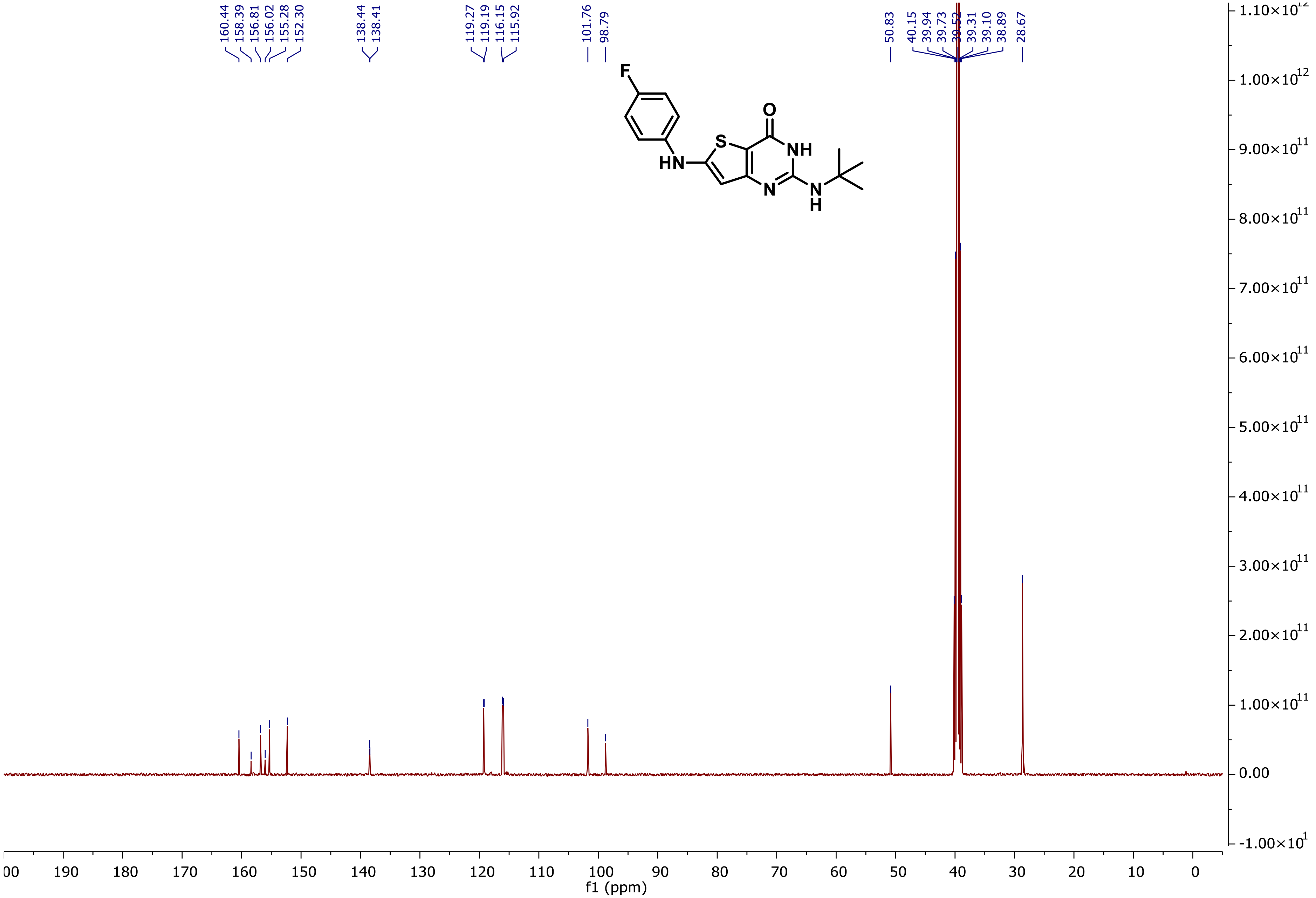

160.44
158.39
156.81
156.02
155.28
152.30
138.44
138.41
119.27
119.19
116.15
115.92
101.76
98.79
50.83
40.15
39.94
39.73
39.52
39.31
39.10
38.89
28.67
F
HN
S
O
NH
N
N
H
f1 (ppm)

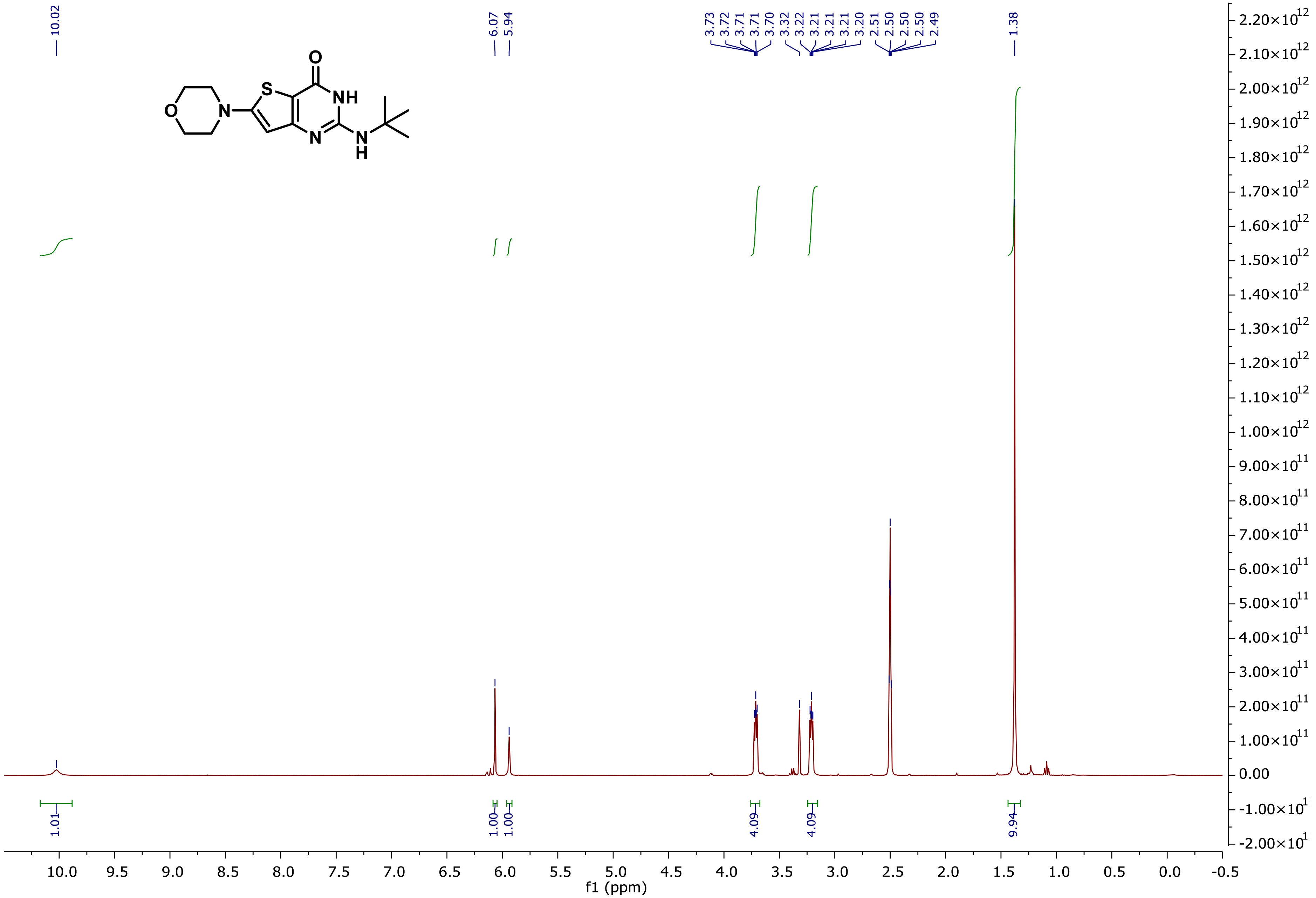

10.02
6.07
5.94
3.73
3.72
3.71
3.71
3.70
3.32
3.22
3.21
3.21
3.21
3.20
2.51
2.50
2.50
2.50
2.49
1.38
O
S
NH
O
N
N
N
H
1.01
1.00
1.00
4.09
4.09
9.94
f1 (ppm)

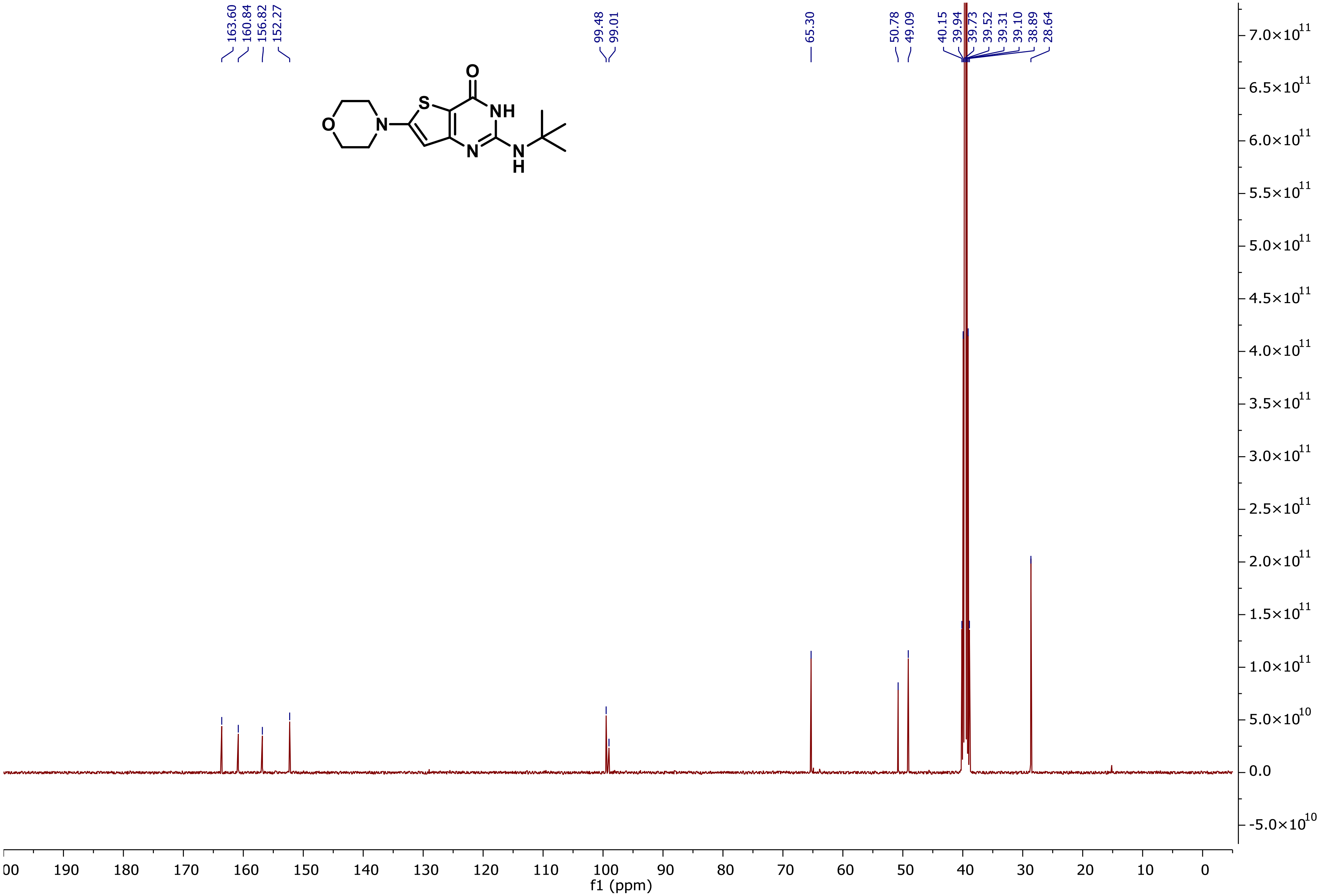
163.60
160.84
156.82
152.27
99.48
99.01
65.30
50.78
49.09
40.15
39.94
39.73
39.52
39.31
39.10
38.89
28.64
O
S
NH
O
N
N
N
H
7.0×10^11
6.5×10^11
6.0×10^11
5.5×10^11
5.0×10^11
4.5×10^11
4.0×10^11
3.5×10^11
3.0×10^11
2.5×10^11
2.0×10^11
1.5×10^11
1.0×10^11
5.0×10^10
0.0
-5.0×10^10
00
190
180
170
160
150
140
130
120
110
100
90
80
70
60
50
40
30
20
10
0
f1 (ppm)

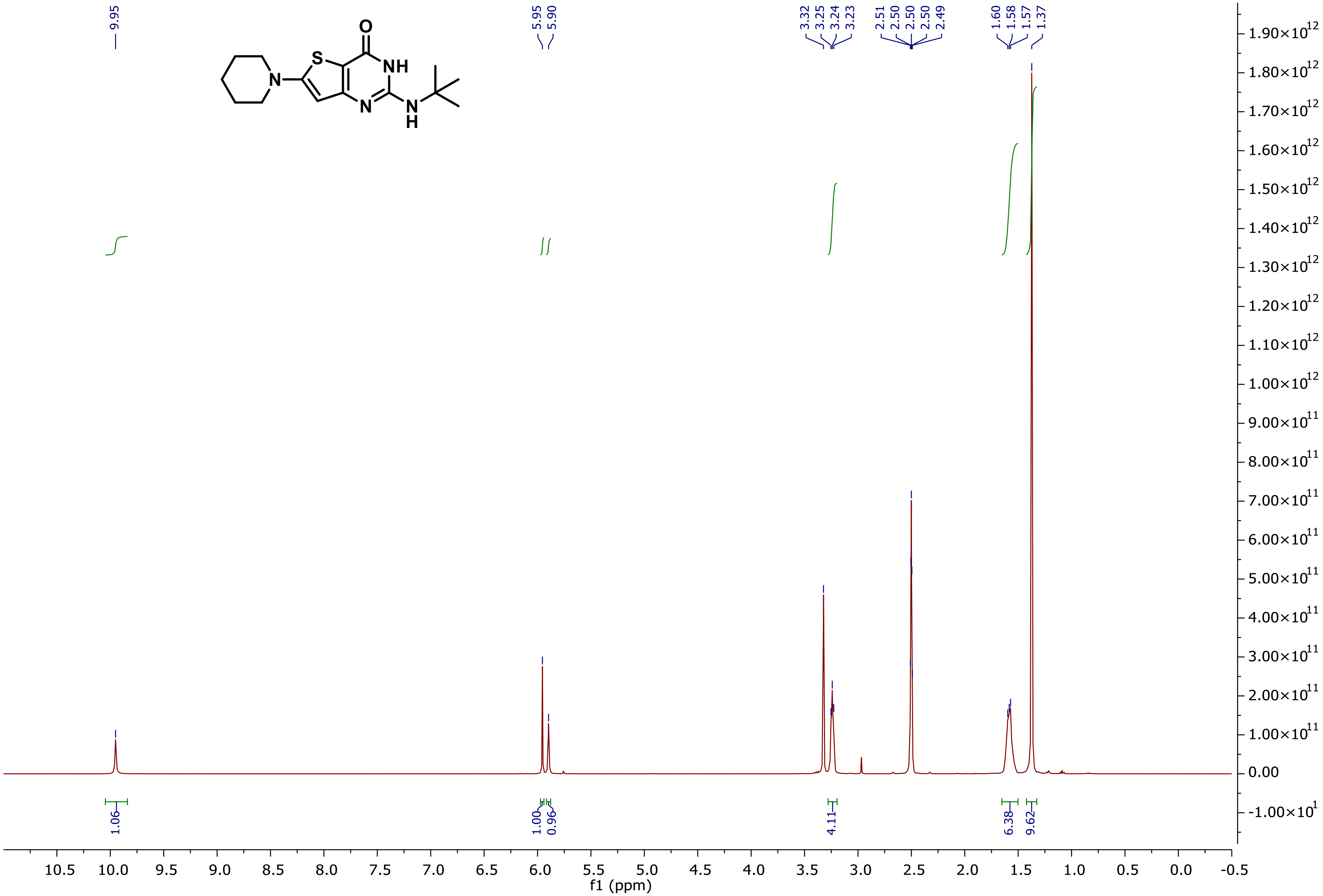

9.95
5.95
5.90
3.32
3.25
3.24
3.23
2.51
2.50
2.50
2.50
2.49
1.60
1.58
1.57
1.37
O
NH
S
N
N
N
H
1.06
1.00
0.96
4.11
6.38
9.62
f1 (ppm)

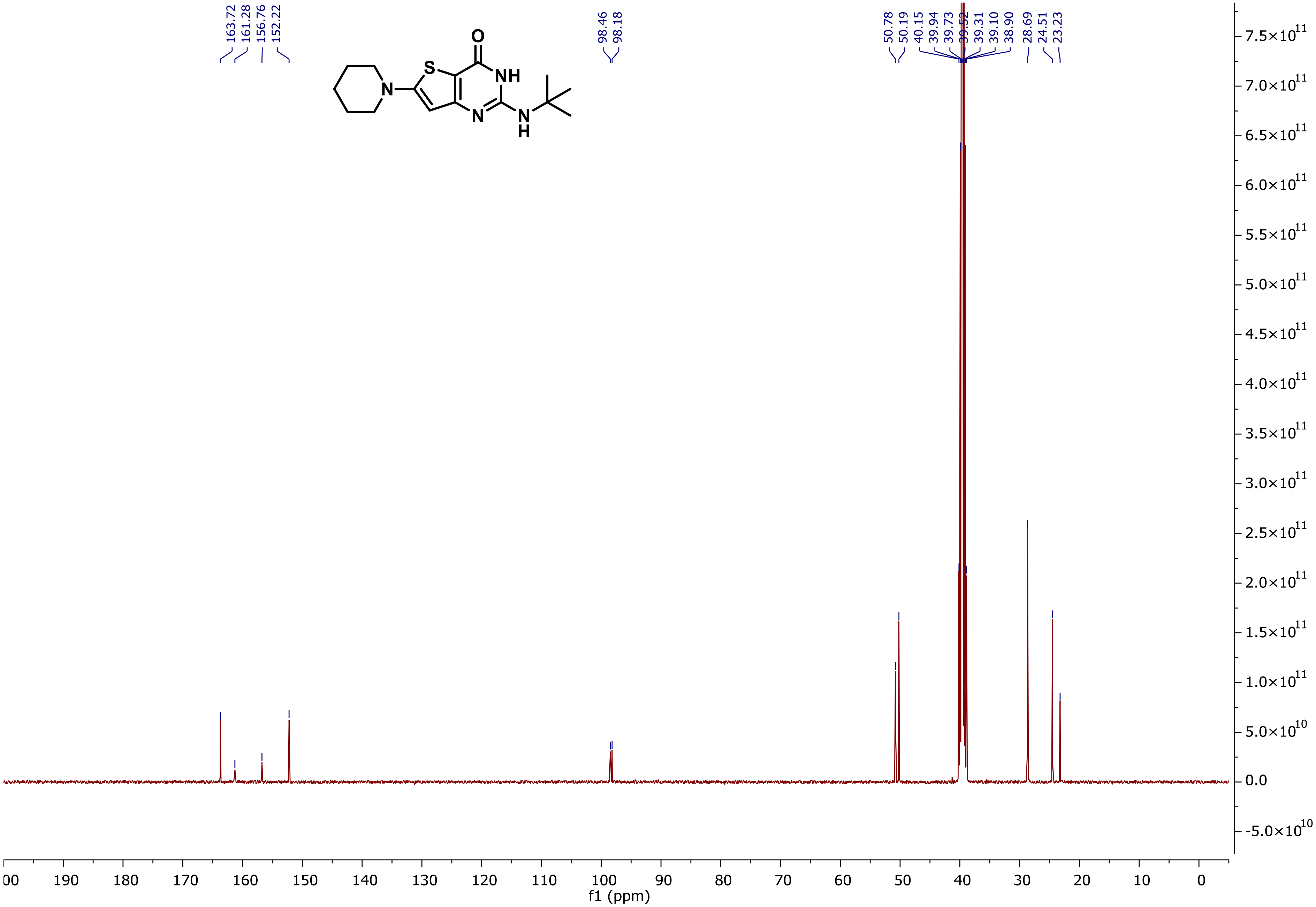
163.72
161.28
156.76
152.22
98.46
98.18
50.78
50.19
40.15
39.94
39.73
39.52
39.31
39.10
38.90
28.69
24.51
23.23
O
NH
S
N
N
N
H
f1 (ppm)

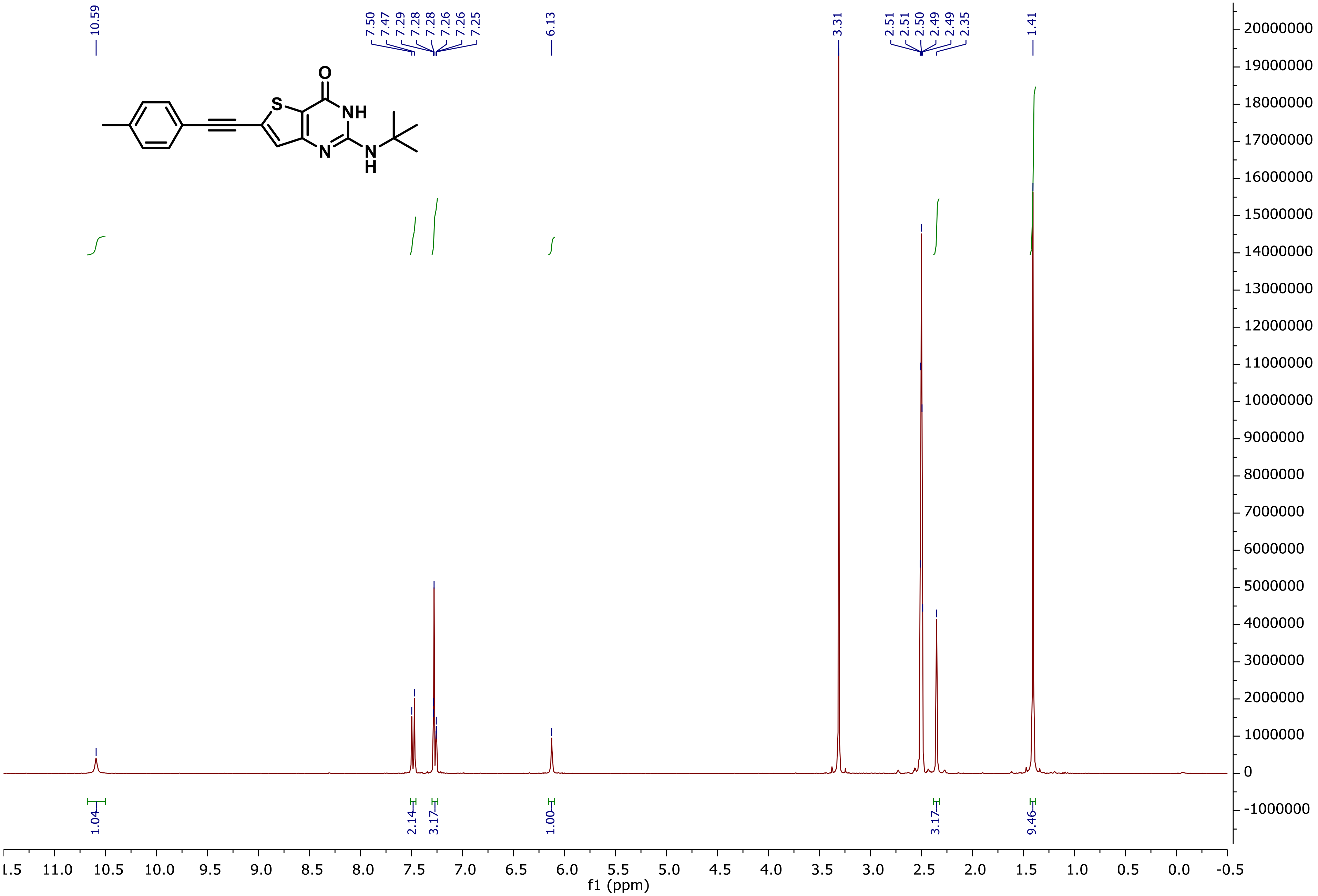
10.59
7.50
7.47
7.29
7.28
7.28
7.26
7.26
7.25
6.13
3.31
2.51
2.51
2.50
2.49
2.49
2.35
1.41
O
S
NH
N
N
H
1.04
2.14
3.17
1.00
3.17
9.46
f1 (ppm)

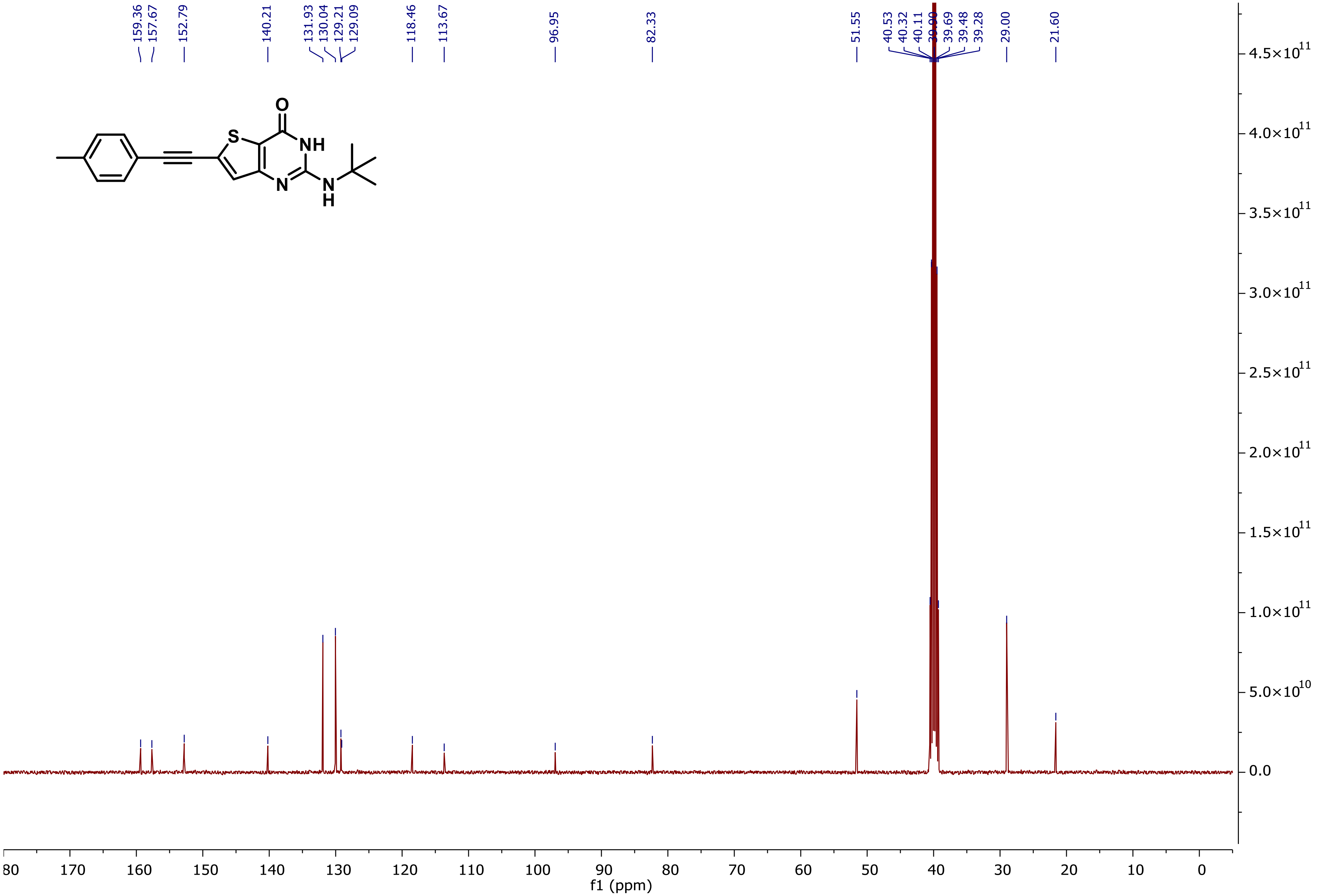

159.36
157.67
152.79
140.21
131.93
130.04
129.21
129.09
118.46
113.67
96.95
82.33
51.55
40.53
40.32
40.11
39.69
39.48
39.28
29.00
21.60
O
S
NH
N
N
H
4.5×10^11
4.0×10^11
3.5×10^11
3.0×10^11
2.5×10^11
2.0×10^11
1.5×10^11
1.0×10^11
5.0×10^10
0.0
80
170
160
150
140
130
120
110
100
90
80
70
60
50
40
30
20
10
0
f1 (ppm)

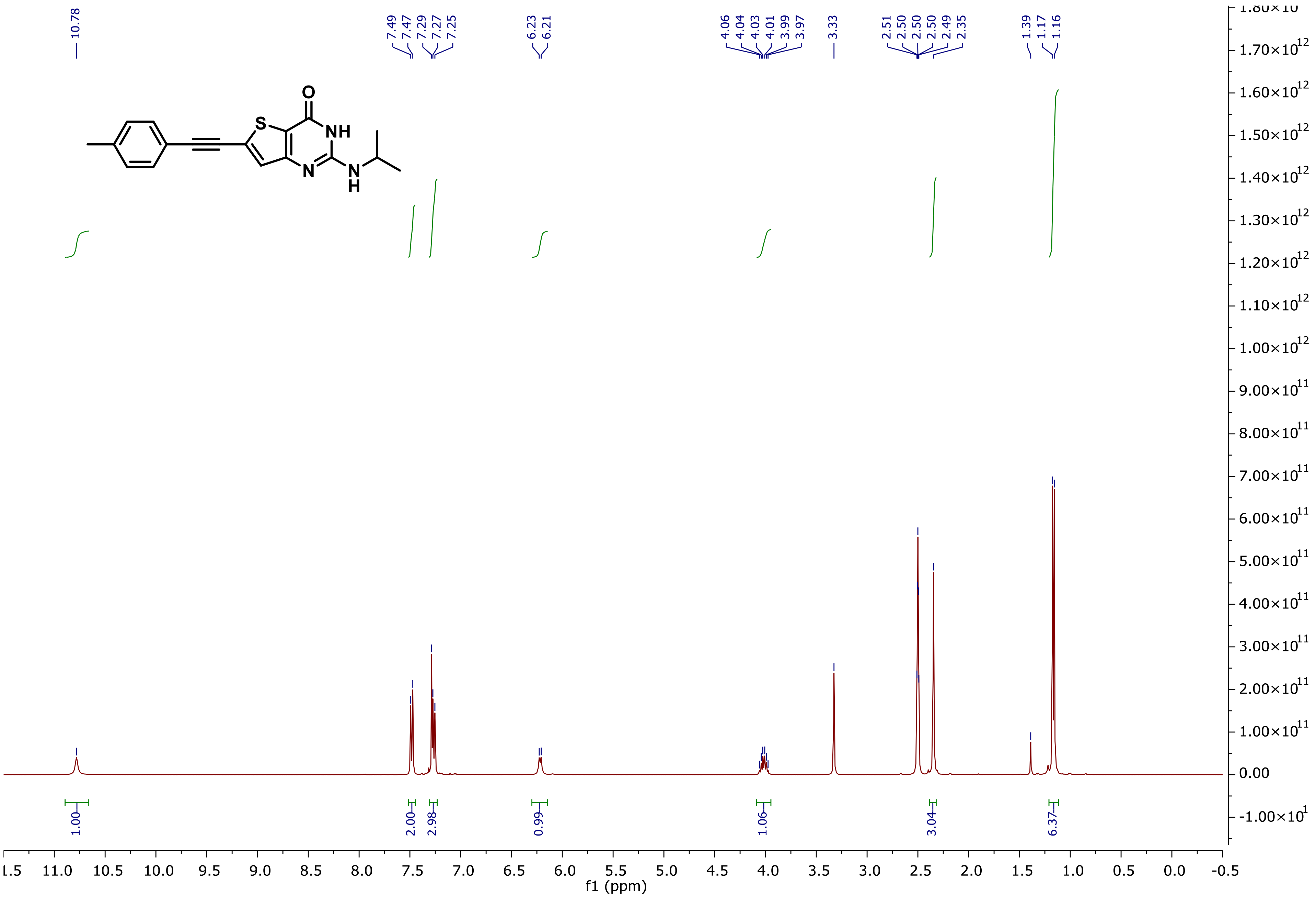
10.78
7.49
7.47
7.29
7.27
7.25
6.23
6.21
4.06
4.04
4.03
4.01
3.99
3.97
3.33
2.51
2.50
2.50
2.50
2.49
2.35
1.39
1.17
1.16
1.00
2.00
2.98
0.99
1.06
3.04
6.37
f1 (ppm)
S
O
NH
N
N
H

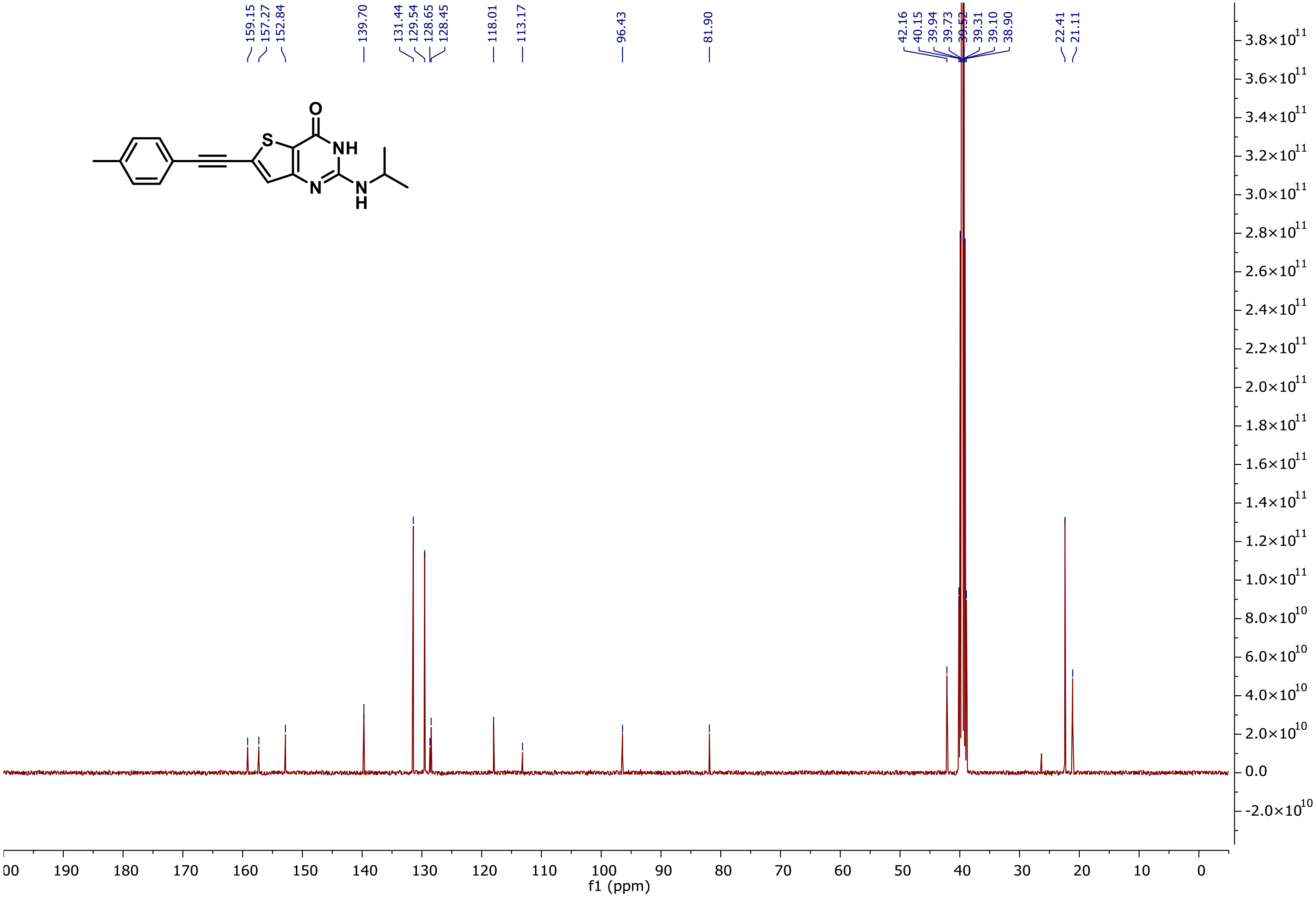
159.15
157.27
152.84
139.70
131.44
129.54
128.65
128.45
118.01
113.17
96.43
81.90
42.16
40.15
39.94
39.73
39.31
39.10
38.90
22.41
21.11
O
S
NH
N
N
H
f1 (ppm)

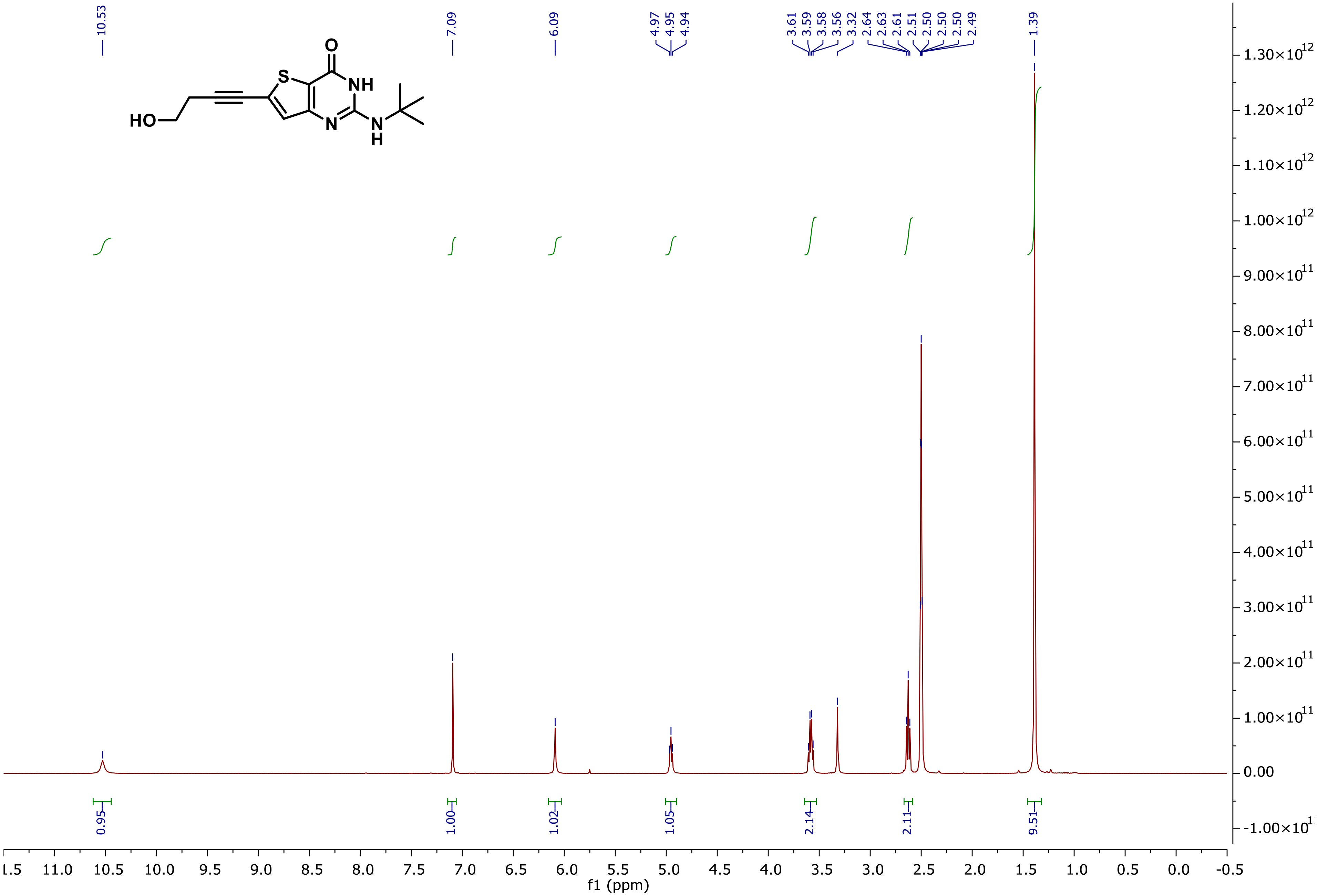

10.53
7.09
6.09
4.97
4.95
4.94
3.61
3.59
3.58
3.56
3.32
2.64
2.63
2.61
2.51
2.50
2.50
2.50
2.49
1.39
0.95
1.00
1.02
1.05
2.14
2.11
9.51
f1 (ppm)

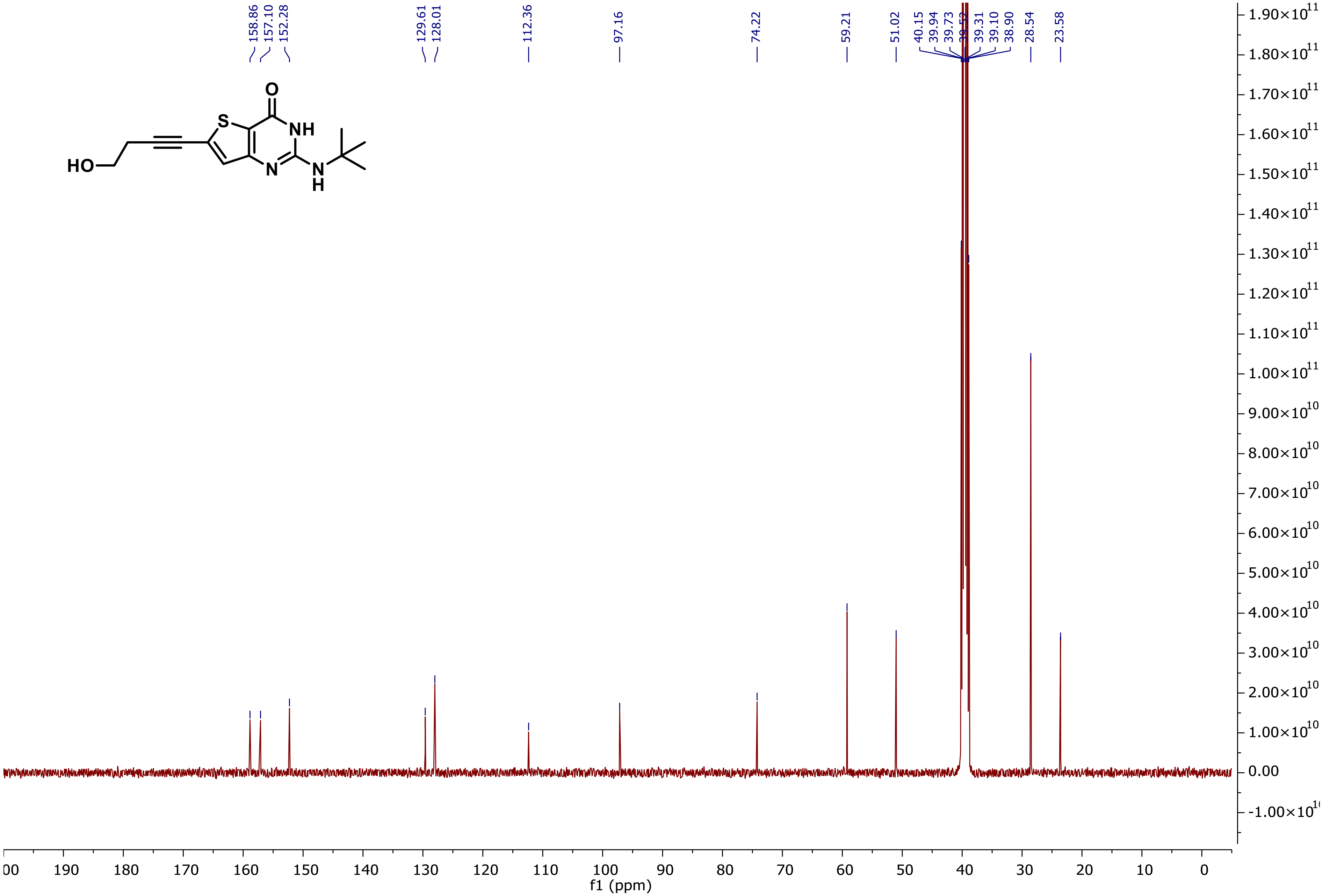

158.86
157.10
152.28
129.61
128.01
112.36
97.16
74.22
59.21
51.02
40.15
39.94
39.73
39.31
39.10
38.90
28.54
23.58
f1 (ppm)

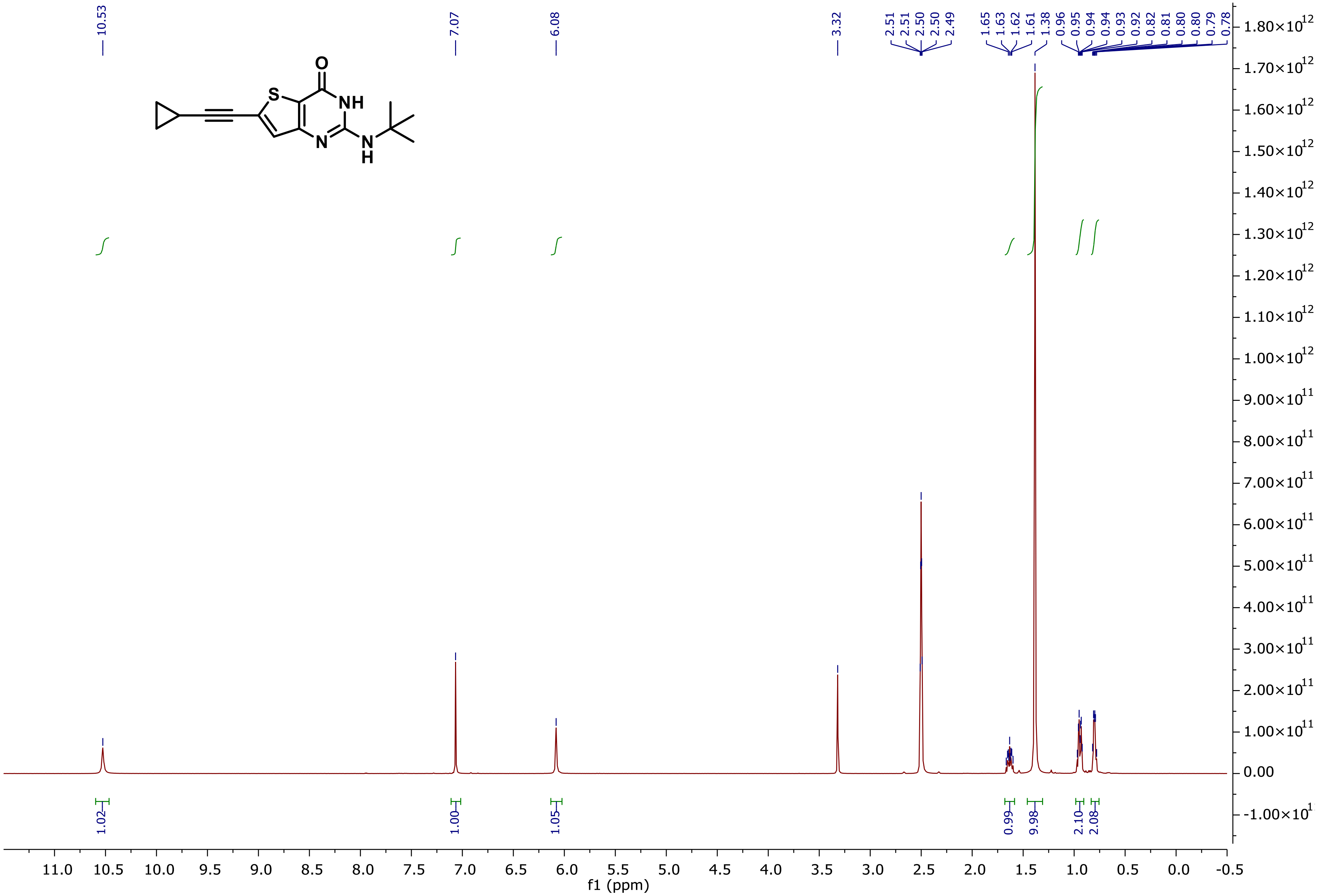

10.53
7.07
6.08
3.32
2.51
2.51
2.50
2.50
2.49
1.65
1.63
1.62
1.61
1.38
0.96
0.95
0.94
0.94
0.93
0.92
0.82
0.81
0.80
0.80
0.79
0.78
O
S
NH
N
N
H
1.02
1.00
1.05
0.99
9.98
2.10
2.08
11.0
10.5
10.0
9.5
9.0
8.5
8.0
7.5
7.0
6.5
6.0
5.5
5.0
4.5
4.0
3.5
3.0
2.5
2.0
1.5
1.0
0.5
0.0
-0.5
f1 (ppm)

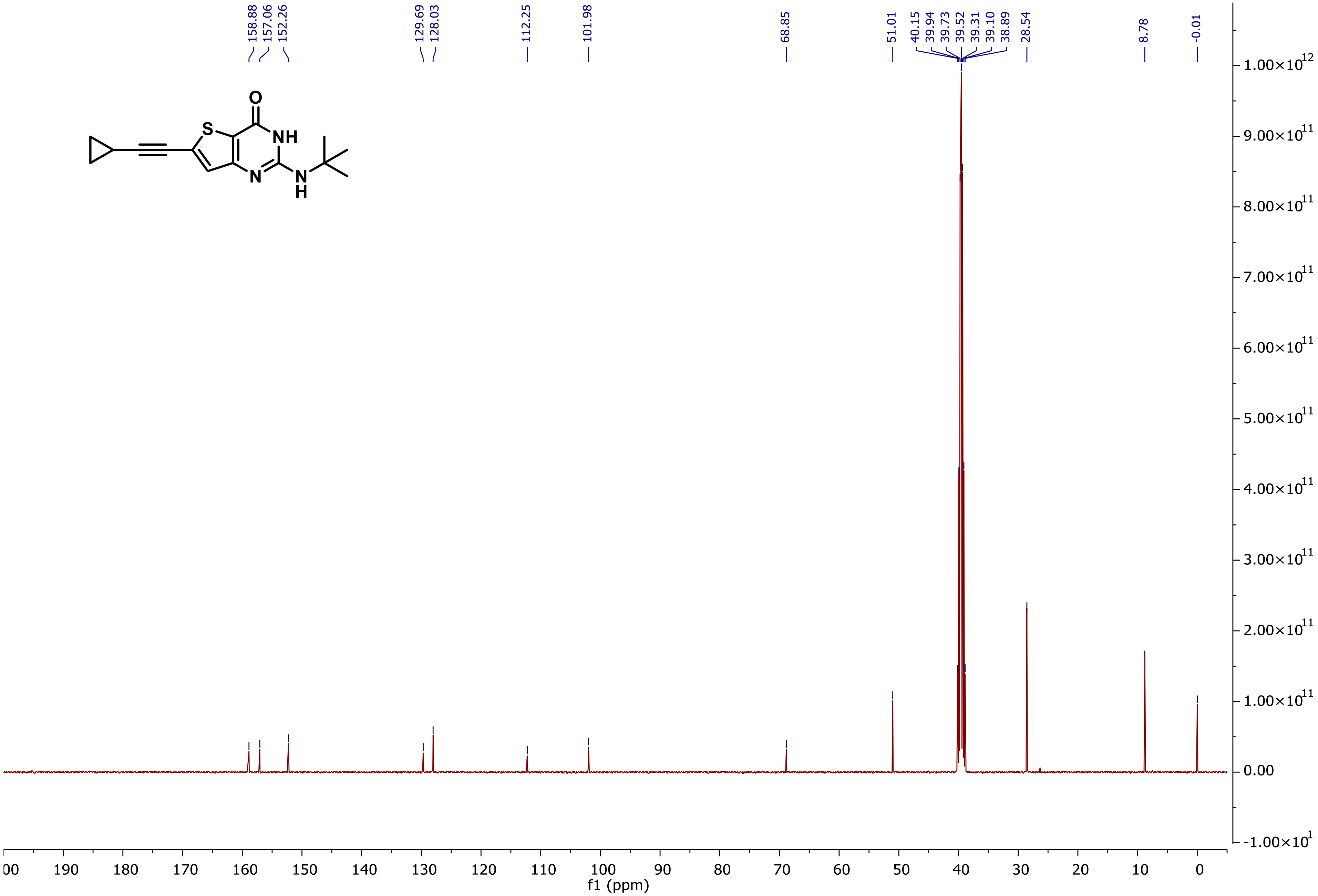

158.88
157.06
152.26
129.69
128.03
112.25
101.98
68.85
51.01
40.15
39.94
39.73
39.52
39.31
39.10
38.89
28.54
8.78
-0.01
O
S
NH
N
N
H
f1 (ppm)

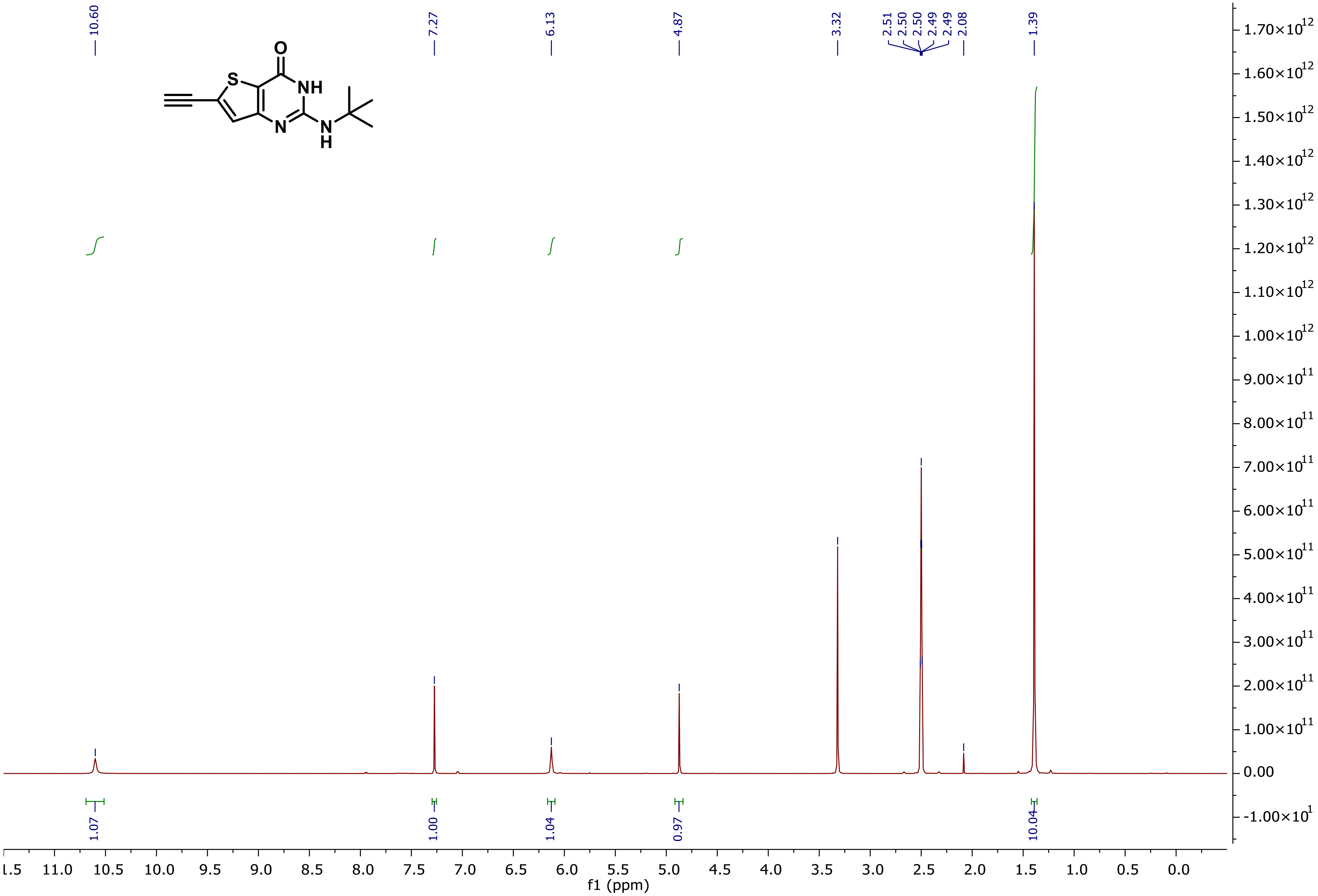
10.60
7.27
6.13
4.87
3.32
2.51
2.50
2.50
2.49
2.49
2.08
1.39
O
S
NH
N
N
H
1.07
1.00
1.04
0.97
10.04
f1 (ppm)

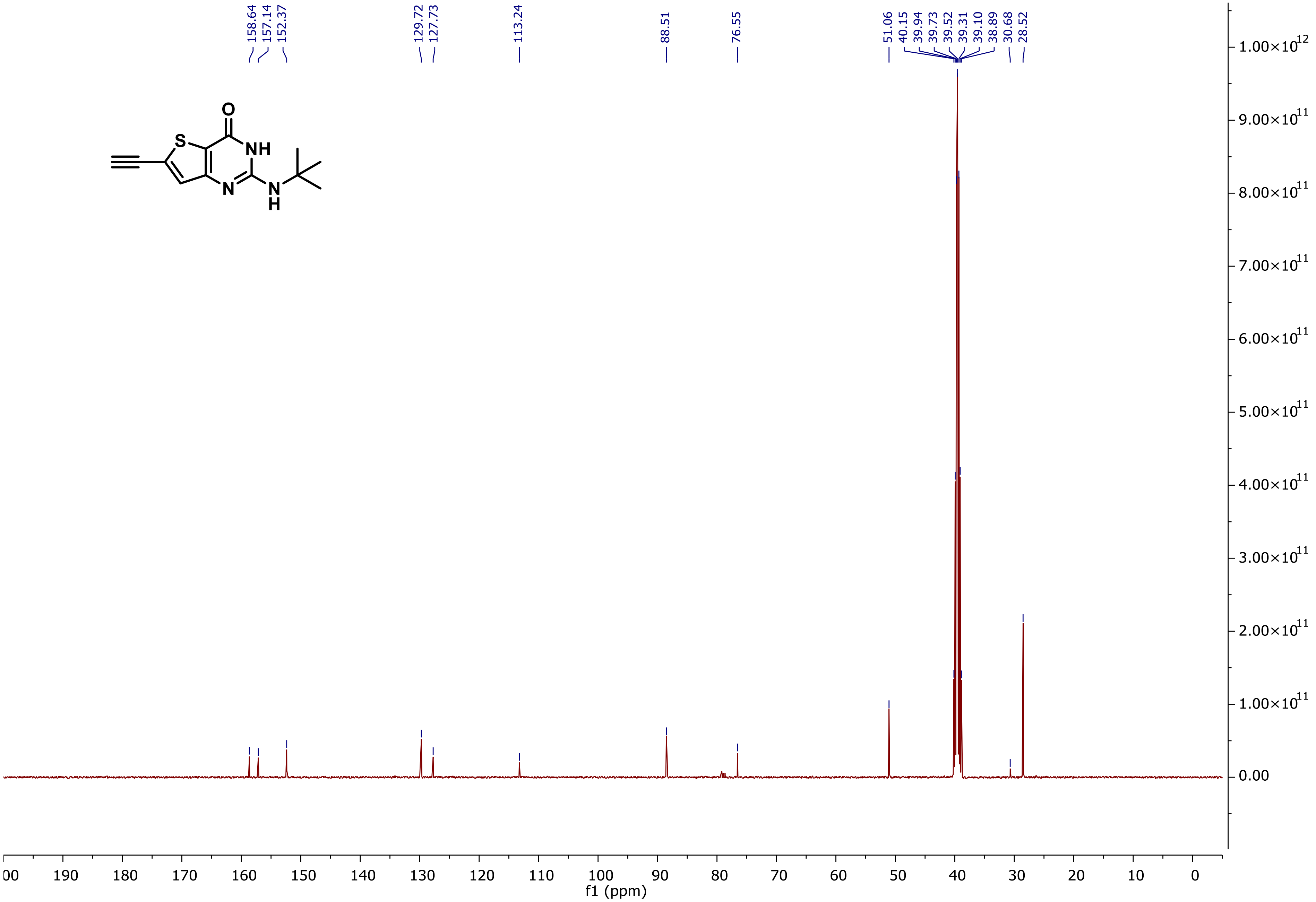
158.64
157.14
152.37
129.72
127.73
113.24
88.51
76.55
51.06
40.15
39.94
39.73
39.52
39.31
39.10
38.89
30.68
28.52
1.00×10^12
9.00×10^11
8.00×10^11
7.00×10^11
6.00×10^11
5.00×10^11
4.00×10^11
3.00×10^11
2.00×10^11
1.00×10^11
0.00
f1 (ppm)
O
S
NH
N
N
H

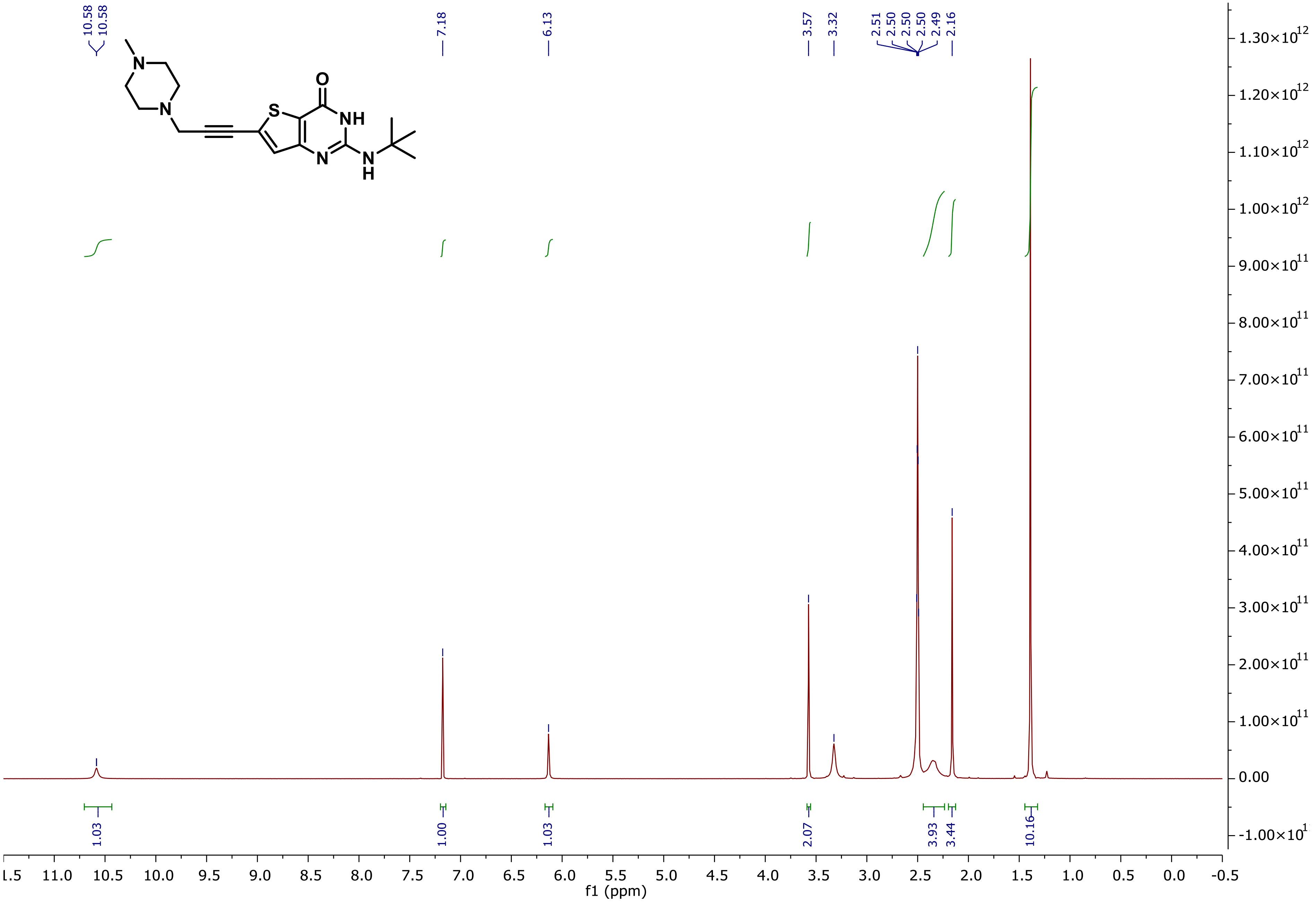
10.58
10.58
7.18
6.13
3.57
3.32
2.51
2.50
2.50
2.50
2.49
2.16
O
S
NH
N
N
N
H
1.03
1.00
1.03
2.07
3.93
3.44
10.16
f1 (ppm)

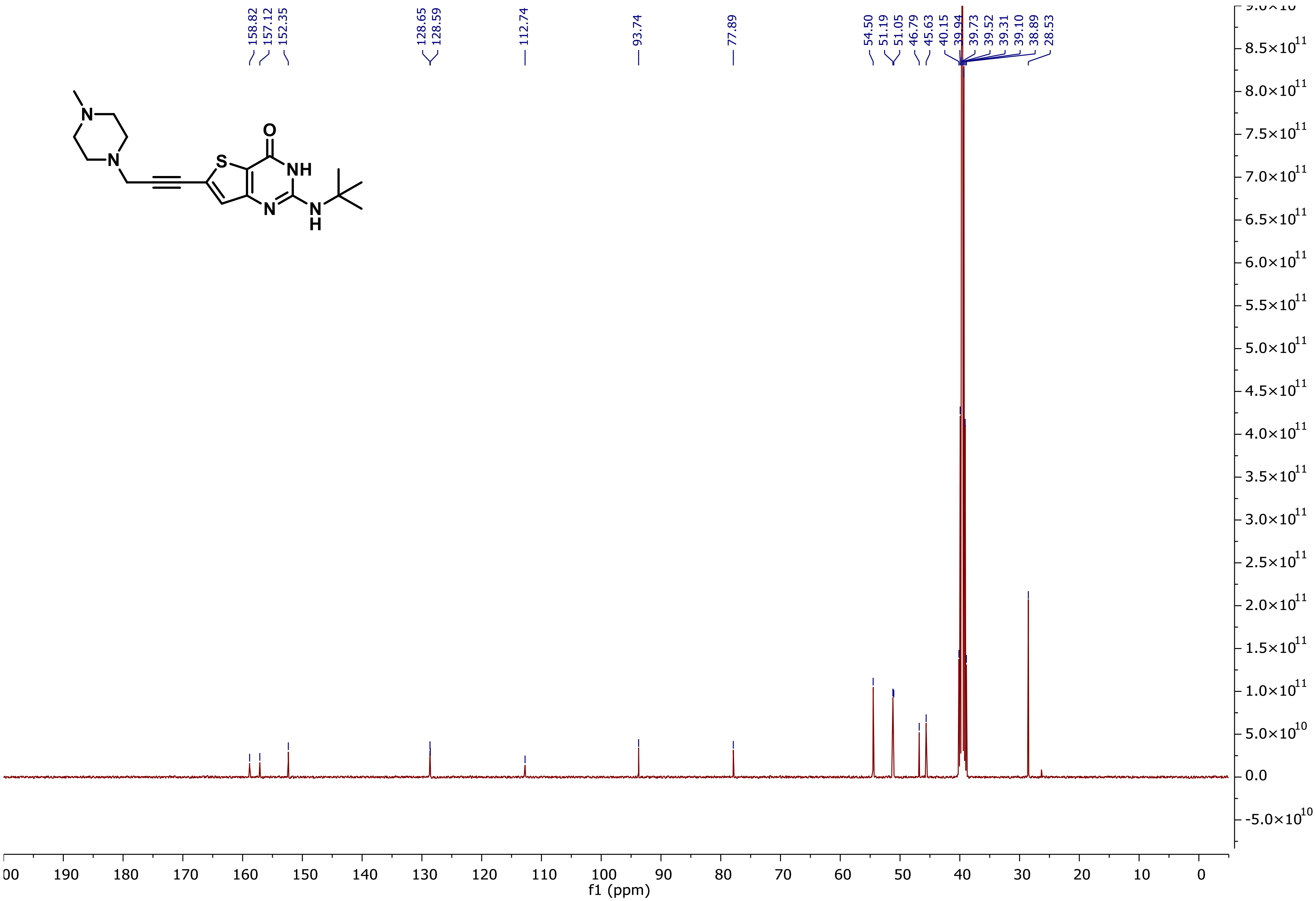
158.82
157.12
152.35
128.65
128.59
112.74
93.74
77.89
54.50
51.19
51.05
46.79
45.63
40.15
39.94
39.73
39.52
39.31
39.10
38.89
28.53
f1 (ppm)

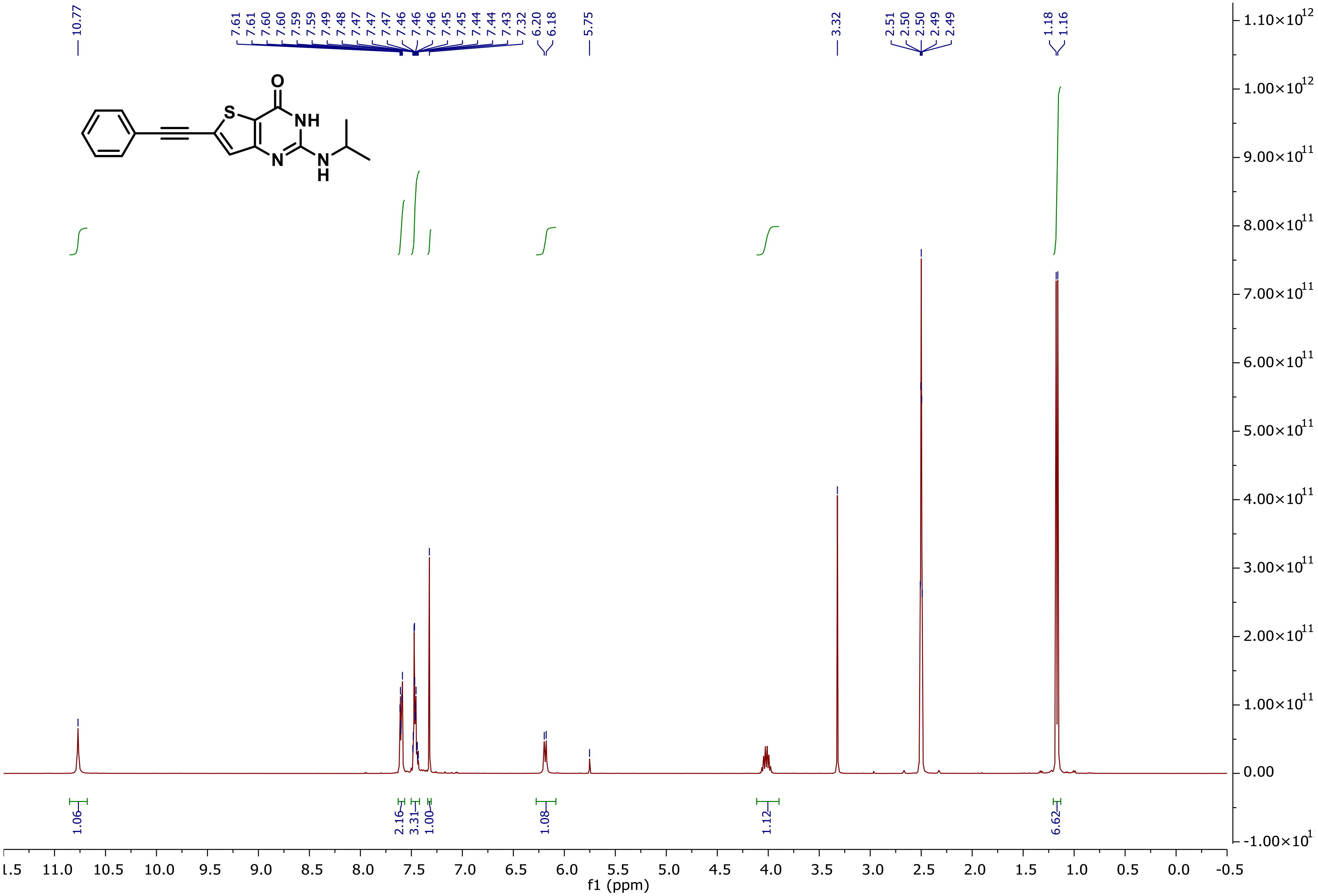

10.77
7.61 7.61 7.60 7.60 7.59 7.59 7.49 7.48 7.47 7.47 7.46 7.46 7.46 7.45 7.45 7.44 7.44 7.43 7.32 6.20 6.18
5.75
3.32
2.51 2.50 2.50 2.49 2.49
1.18 1.16
O
S
NH
N
N
H
1.06
2.16
3.31
1.00
1.08
1.12
6.62
f1 (ppm)

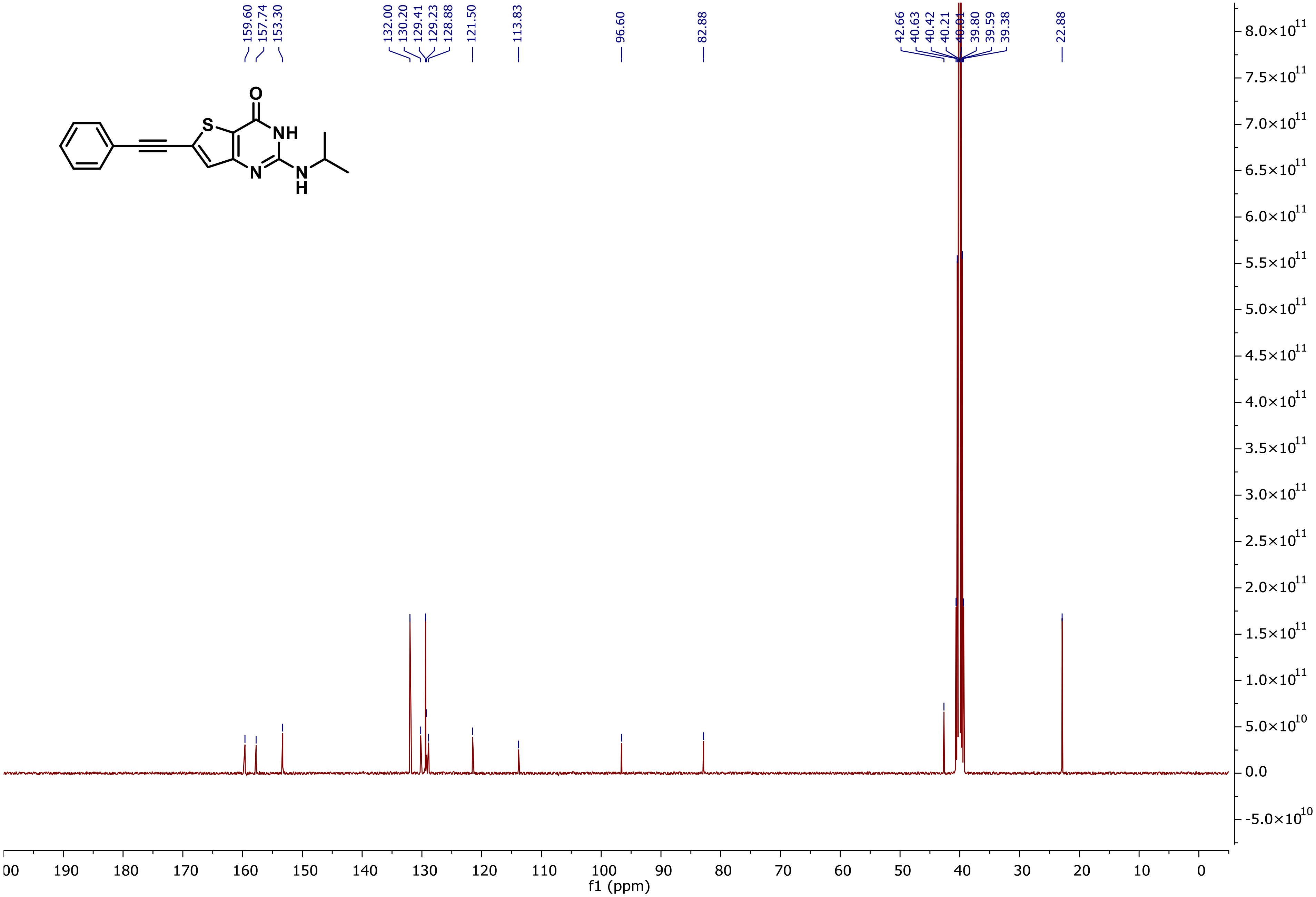

159.60
157.74
153.30
132.00
130.20
129.41
129.23
128.88
121.50
113.83
96.60
82.88
42.66
40.63
40.42
40.21
40.01
39.80
39.59
39.38
22.88
O
S
NH
N
N
H
f1 (ppm)

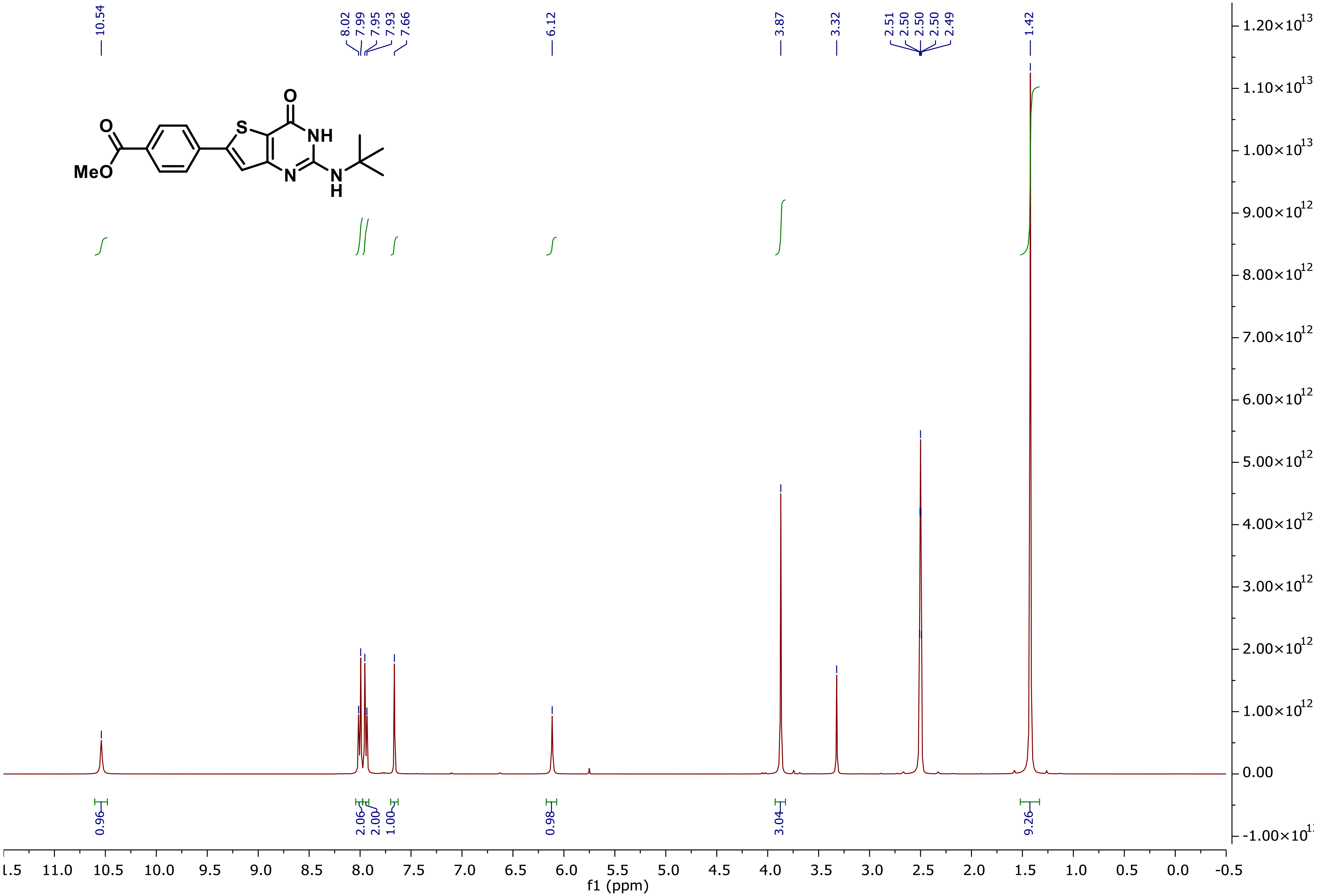

10.54
8.02
7.99
7.95
7.93
7.66
6.12
3.87
3.32
2.51
2.50
2.50
2.50
2.49
1.42
O
S
NH
MeO
N
N
H
0.96
2.06
2.00
1.00
0.98
3.04
9.26
f1 (ppm)

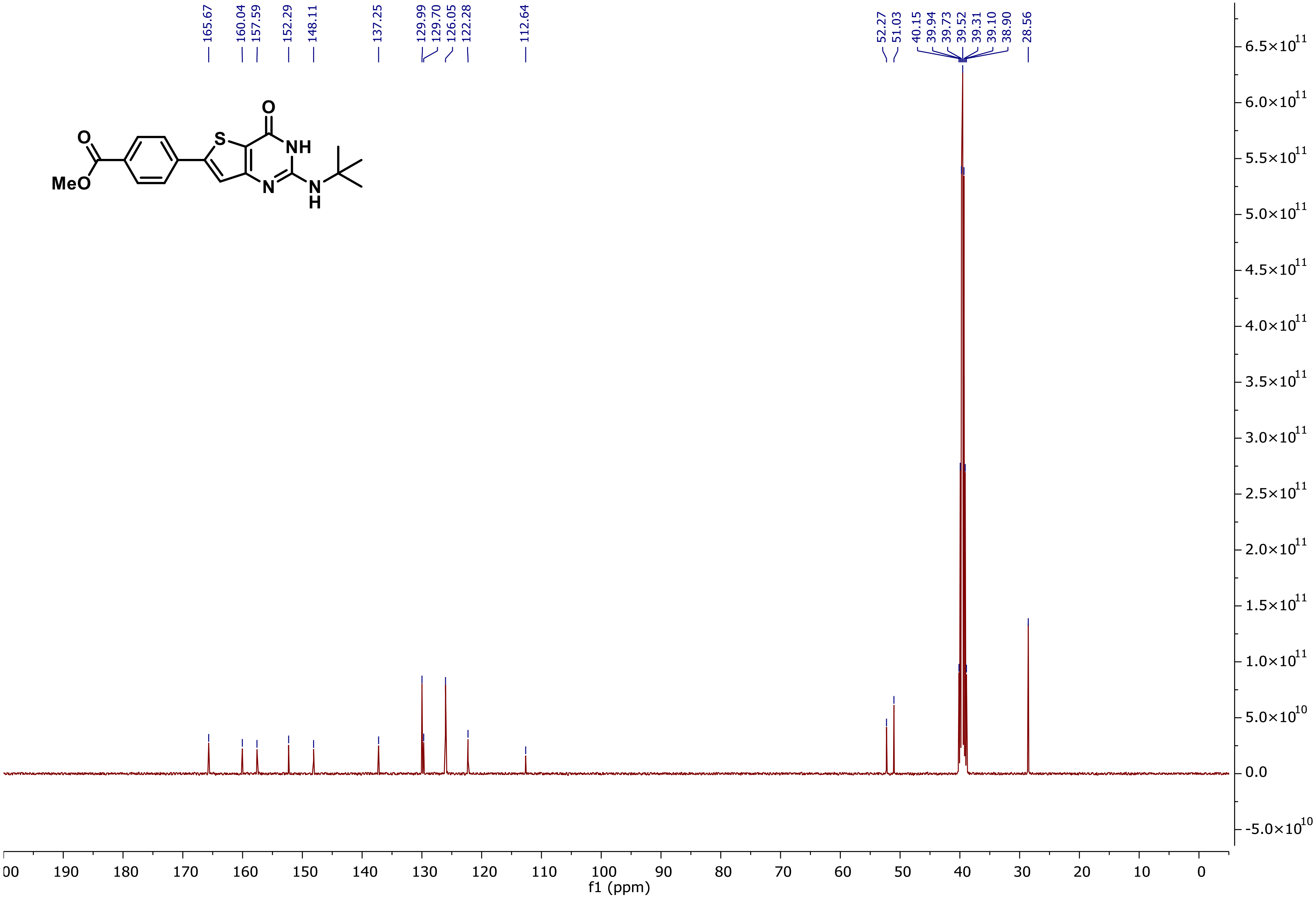
165.67
160.04
157.59
152.29
148.11
137.25
129.99
129.70
126.05
122.28
112.64
52.27
51.03
40.15
39.94
39.73
39.52
39.31
39.10
38.90
28.56
O
S
NH
MeO
N
N
H
f1 (ppm)

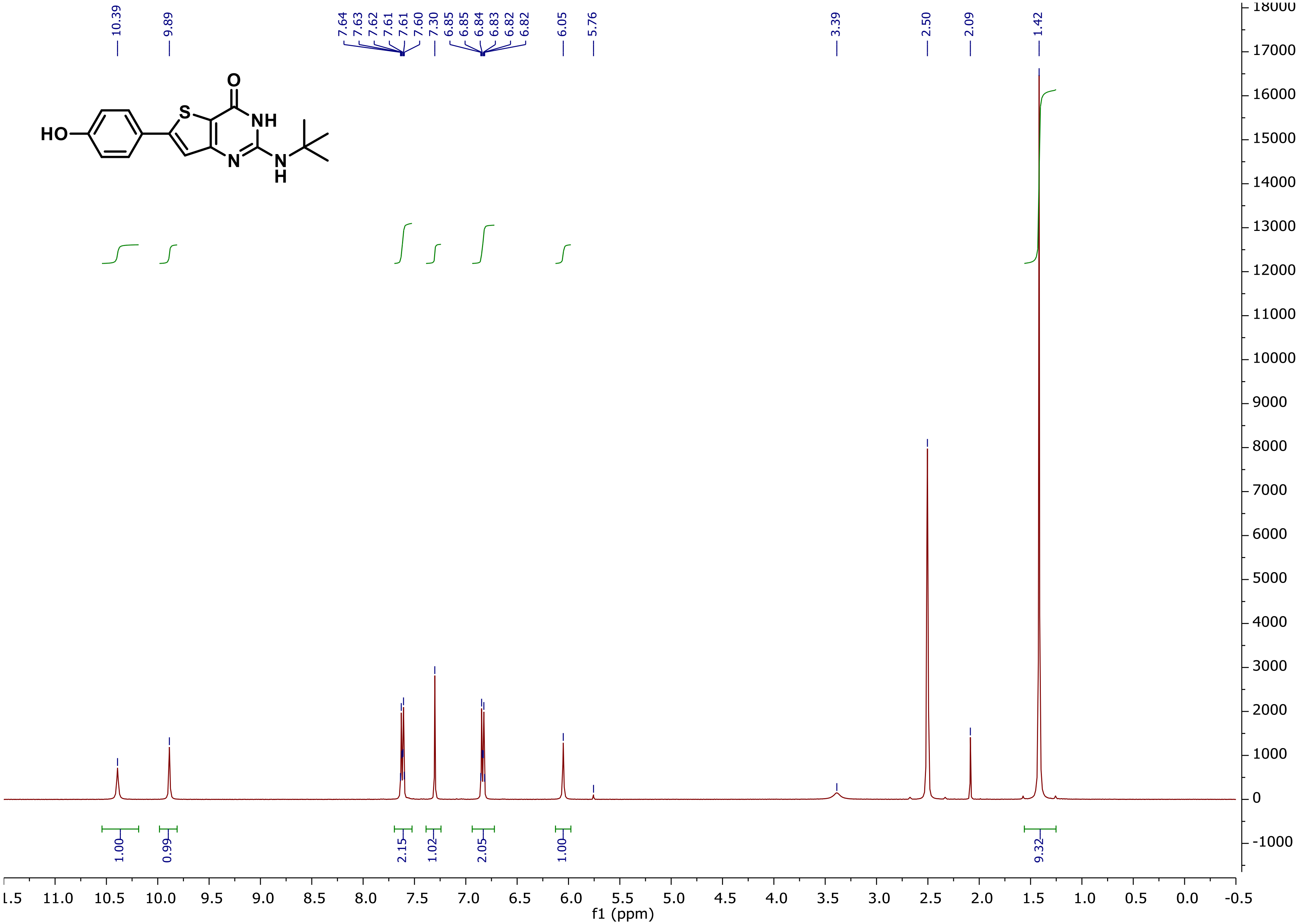

10.39
9.89
7.64
7.63
7.62
7.61
7.61
7.60
7.30
6.85
6.85
6.84
6.83
6.82
6.82
6.05
5.76
3.39
2.50
2.09
1.42
HO
S
O
NH
N
N
H
1.00
0.99
2.15
1.02
2.05
1.00
9.32
f1 (ppm)

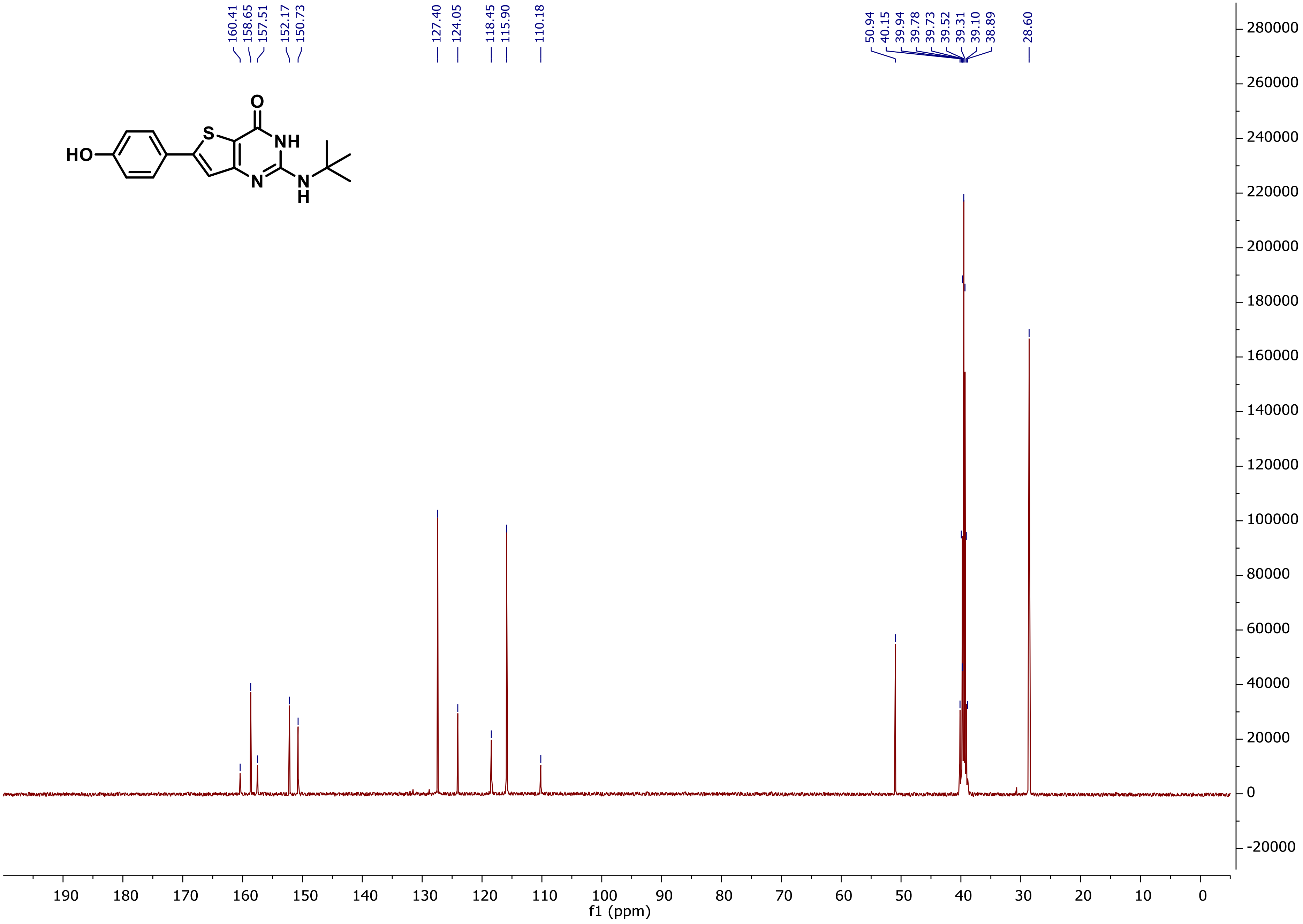

160.41
158.65
157.51
152.17
150.73
127.40
124.05
118.45
115.90
110.18
50.94
40.15
39.94
39.78
39.73
39.52
39.31
39.10
38.89
28.60
HO
S
O
NH
N
N
H
280000
260000
240000
220000
200000
180000
160000
140000
120000
100000
80000
60000
40000
20000
0
-20000
190
180
170
160
150
140
130
120
110
100
90
80
70
60
50
40
30
20
10
0
f1 (ppm)

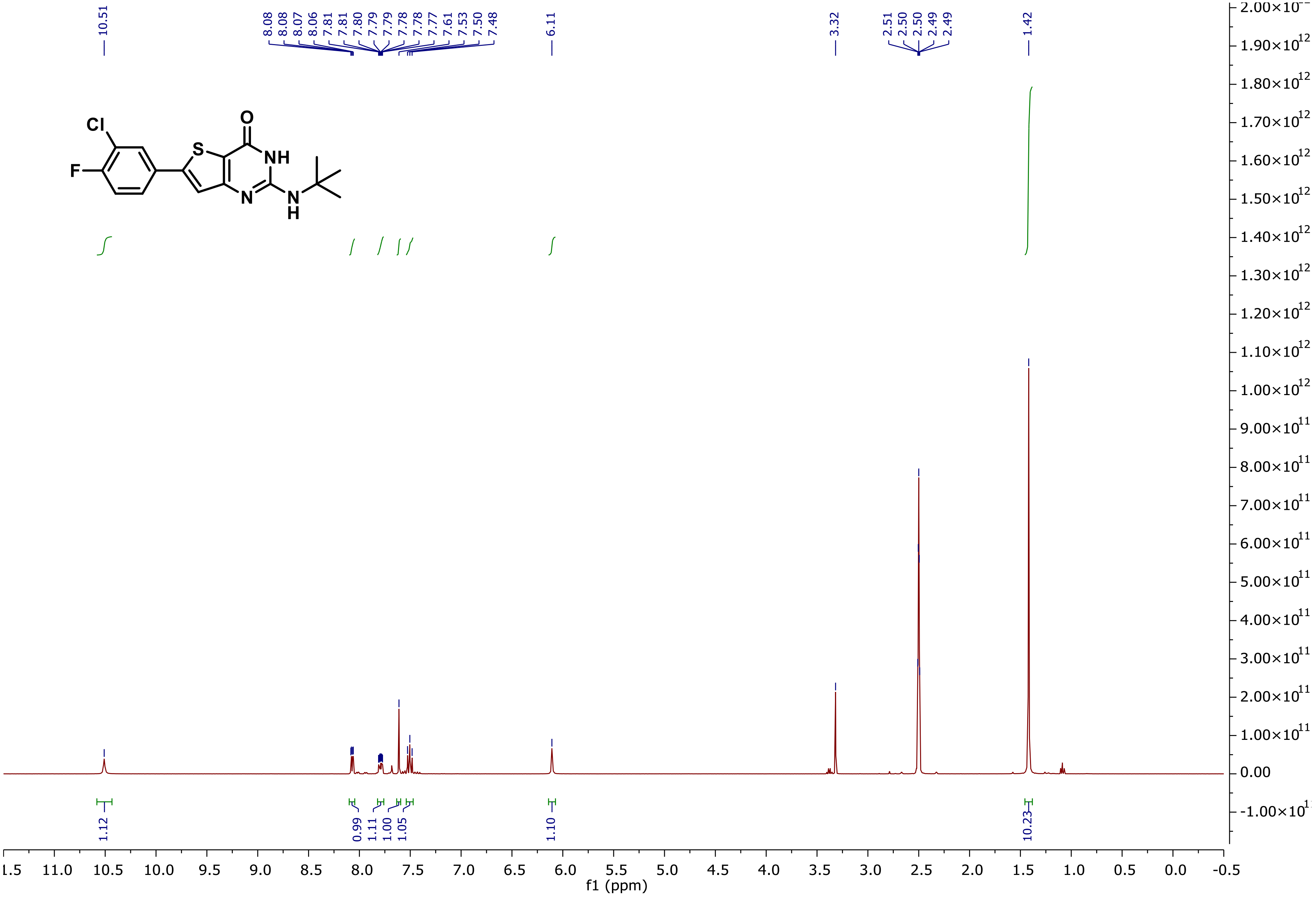
10.51
8.08
8.08
8.07
8.06
7.81
7.81
7.80
7.79
7.79
7.78
7.77
7.61
7.53
7.50
7.48
6.11
3.32
2.51
2.50
2.50
2.49
2.49
1.42
1.12
0.99
1.11
1.00
1.05
1.10
10.23
f1 (ppm)

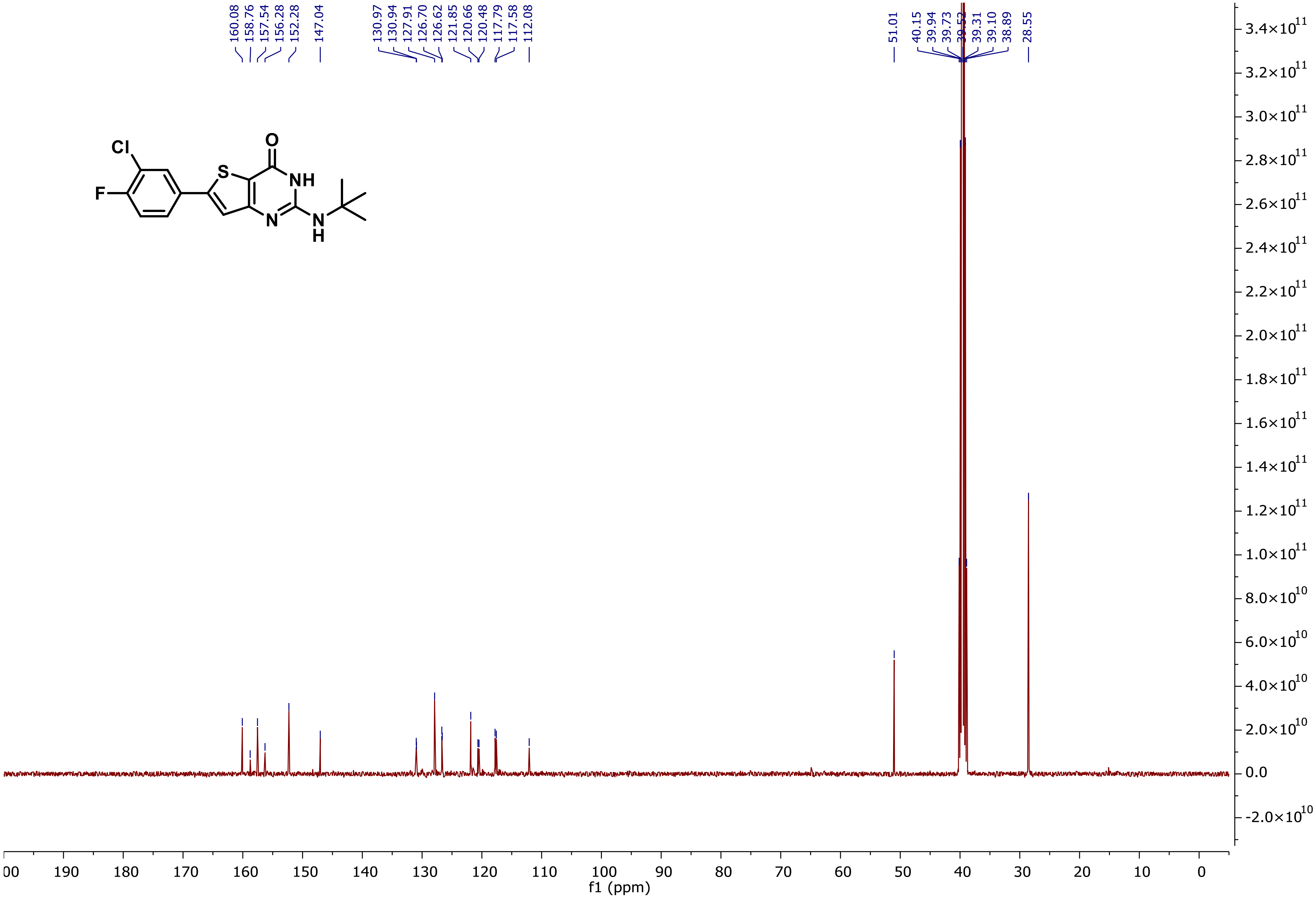

160.08
158.76
157.54
156.28
152.28
147.04
130.97
130.94
127.91
126.70
126.62
121.85
120.66
120.48
117.79
117.58
112.08
51.01
40.15
39.94
39.73
39.52
39.31
39.10
38.89
28.55
Cl
F
S
O
NH
N
N
H
3.4×10^11
3.2×10^11
3.0×10^11
2.8×10^11
2.6×10^11
2.4×10^11
2.2×10^11
2.0×10^11
1.8×10^11
1.6×10^11
1.4×10^11
1.2×10^11
1.0×10^11
8.0×10^10
6.0×10^10
4.0×10^10
2.0×10^10
0.0
-2.0×10^10
00
190
180
170
160
150
140
130
120
110
100
90
80
70
60
50
40
30
20
10
0
f1 (ppm)

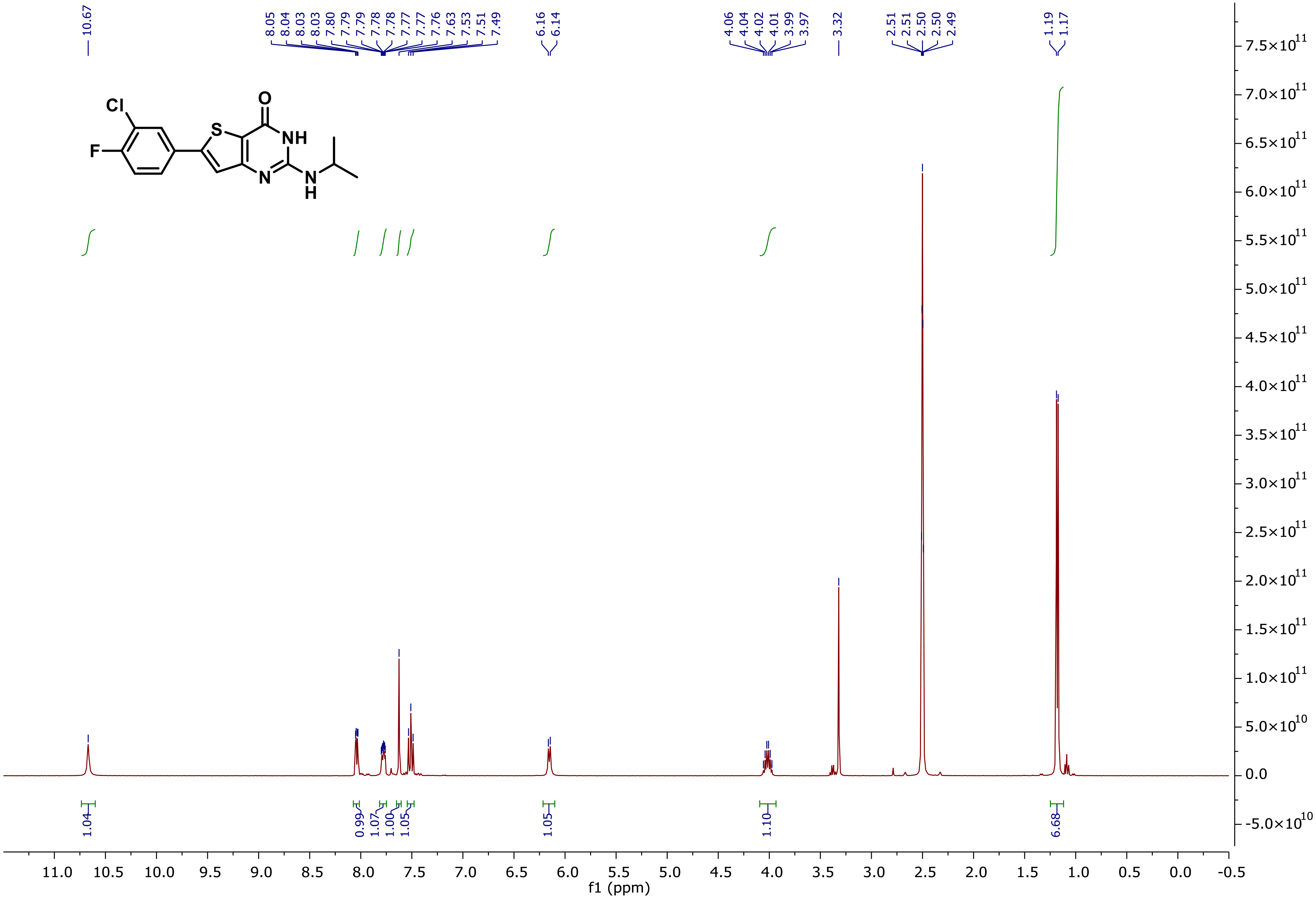
10.67
8.05
8.04
8.03
8.03
7.80
7.79
7.79
7.78
7.78
7.77
7.77
7.76
7.63
7.53
7.51
7.49
6.16
6.14
4.06
4.04
4.02
4.01
3.99
3.97
3.32
2.51
2.51
2.50
2.50
2.49
1.19
1.17
Cl
F
S
O
NH
N
N
H
1.04
0.99
1.07
1.00
1.05
1.05
1.10
6.68
f1 (ppm)

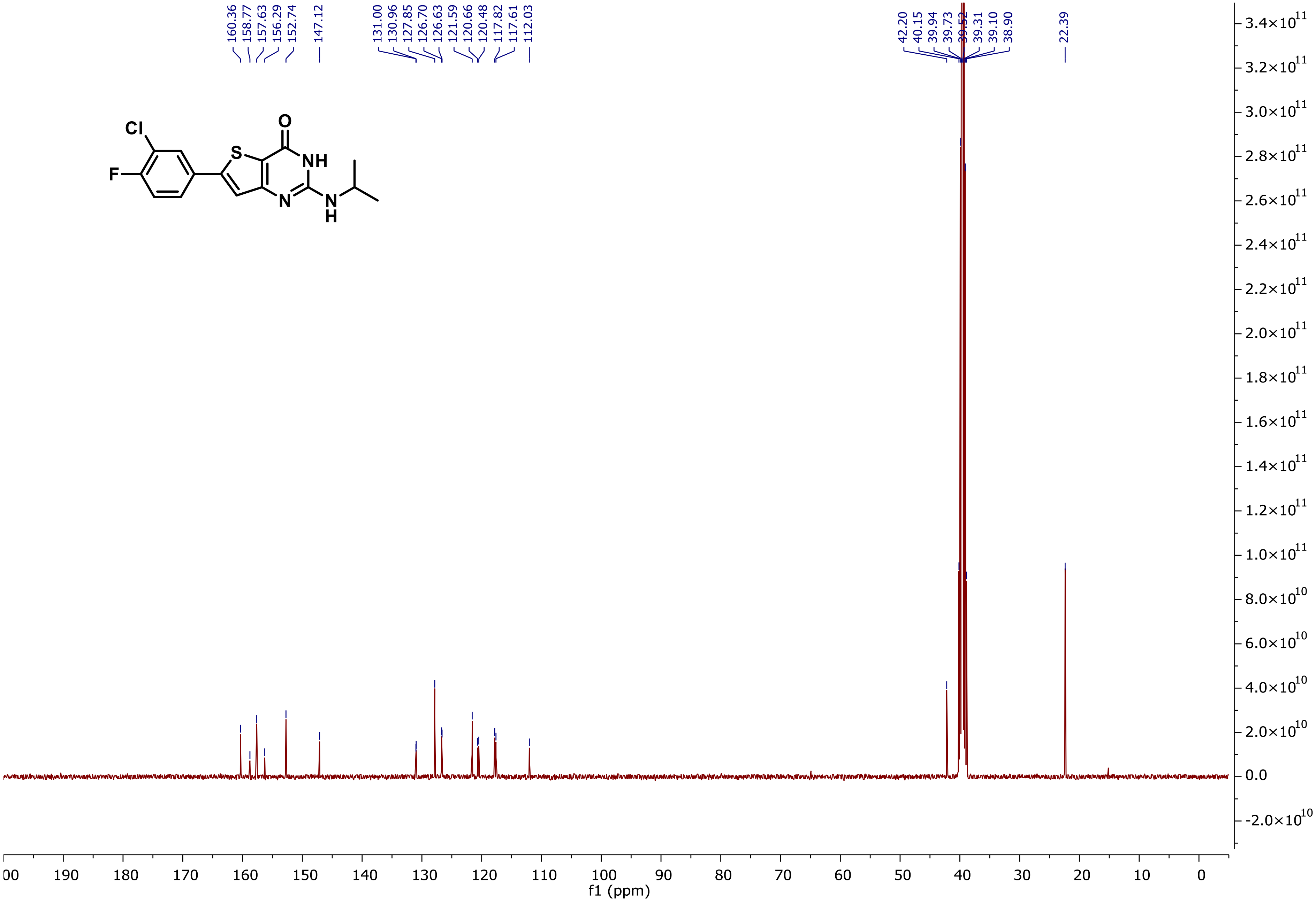
160.36
158.77
157.63
156.29
152.74
147.12
131.00
130.96
127.85
126.70
126.63
121.59
120.66
120.48
117.82
117.61
112.03
42.20
40.15
39.94
39.73
39.52
39.31
39.10
38.90
22.39
Cl
F
S
O
NH
N
N
H
f1 (ppm)

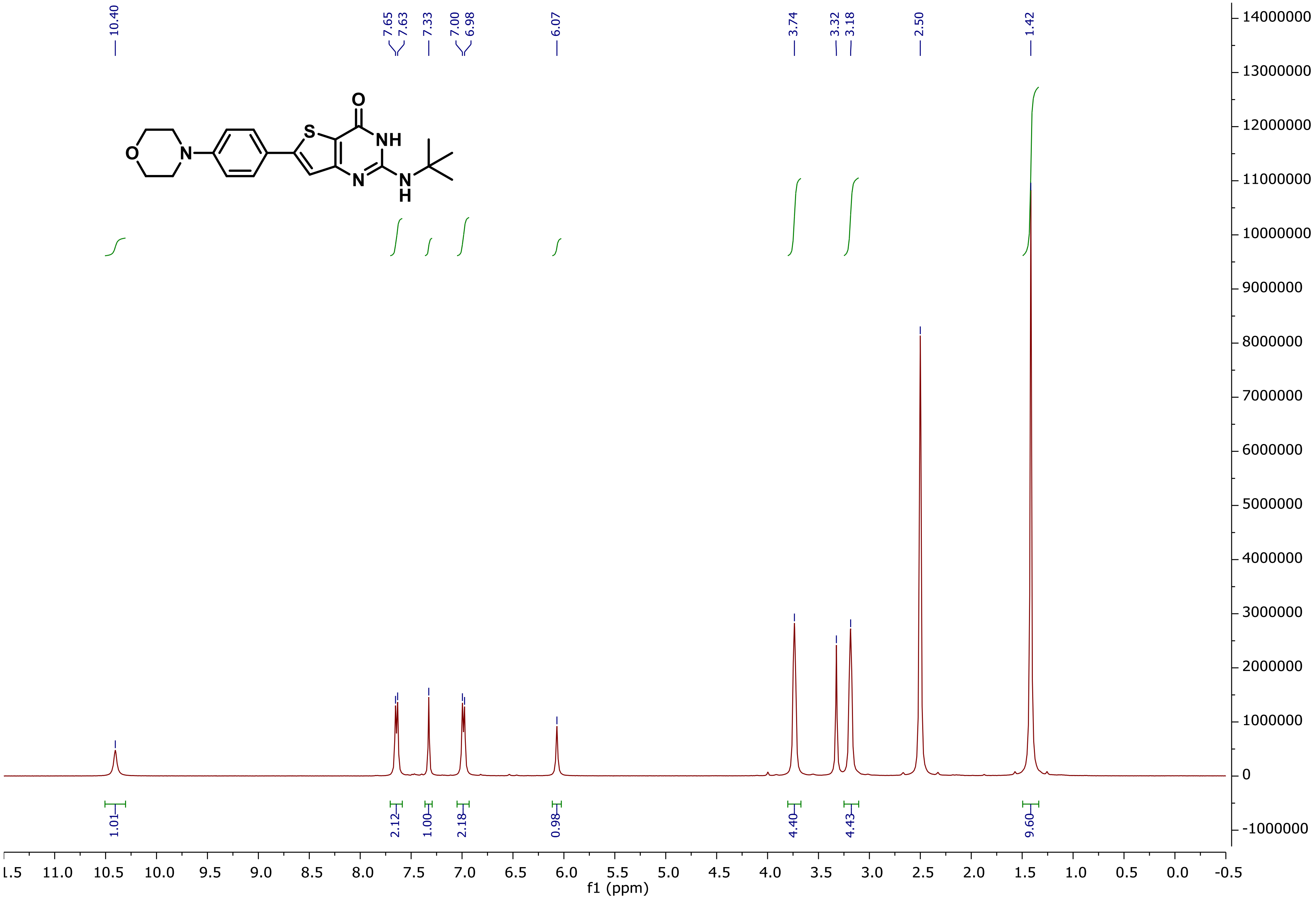
10.40
7.65
7.63
7.33
7.00
6.98
6.07
3.74
3.32
3.18
2.50
1.42
1.01
2.12
1.00
2.18
0.98
4.40
4.43
9.60
f1 (ppm)

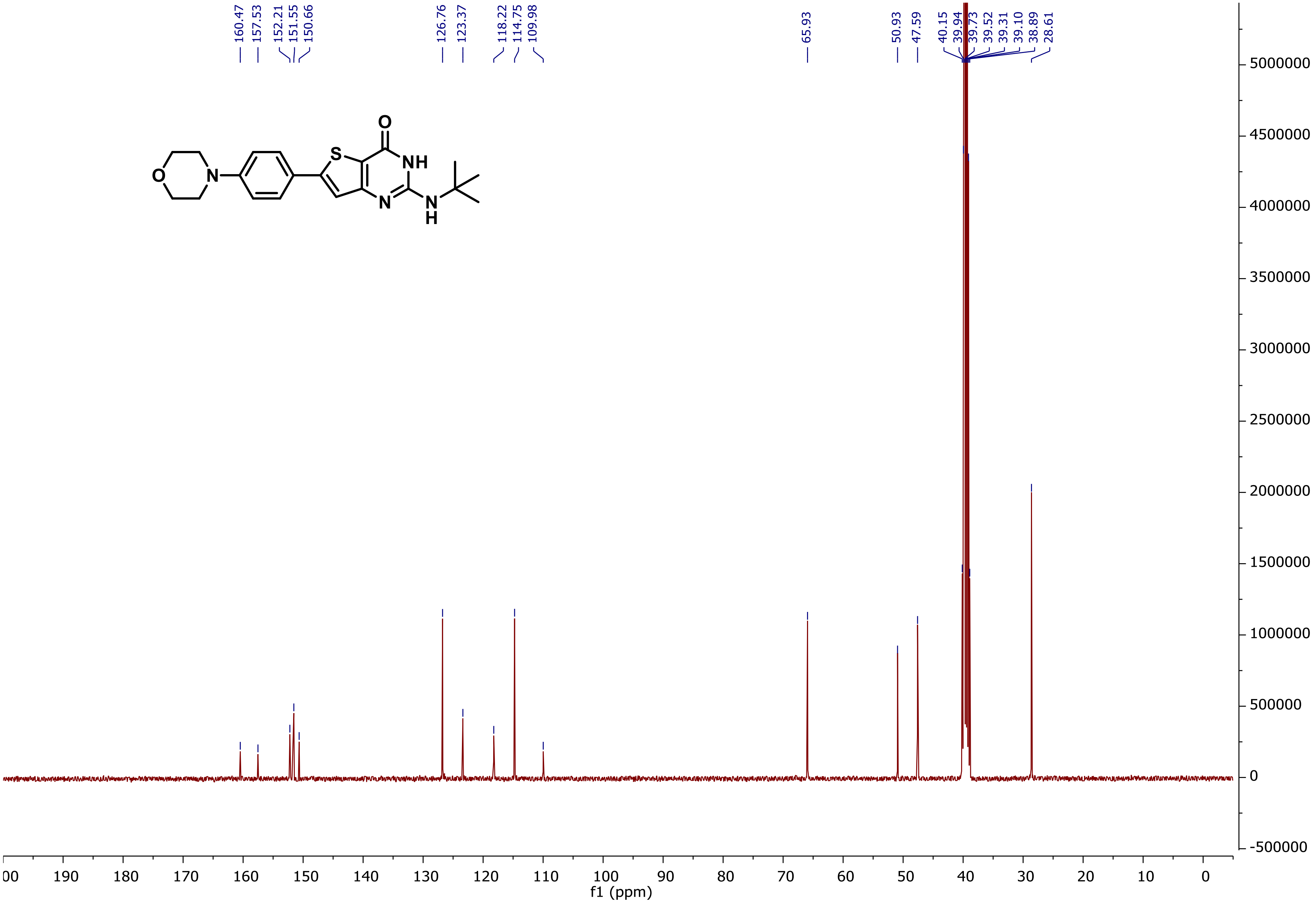

160.47
157.53
152.21
151.55
150.66
126.76
123.37
118.22
114.75
109.98
65.93
50.93
47.59
40.15
39.94
39.73
39.52
39.31
39.10
38.89
28.61
5000000
4500000
4000000
3500000
3000000
2500000
2000000
1500000
1000000
500000
0
-500000
00
190
180
170
160
150
140
130
120
110
100
90
80
70
60
50
40
30
20
10
0
f1 (ppm)

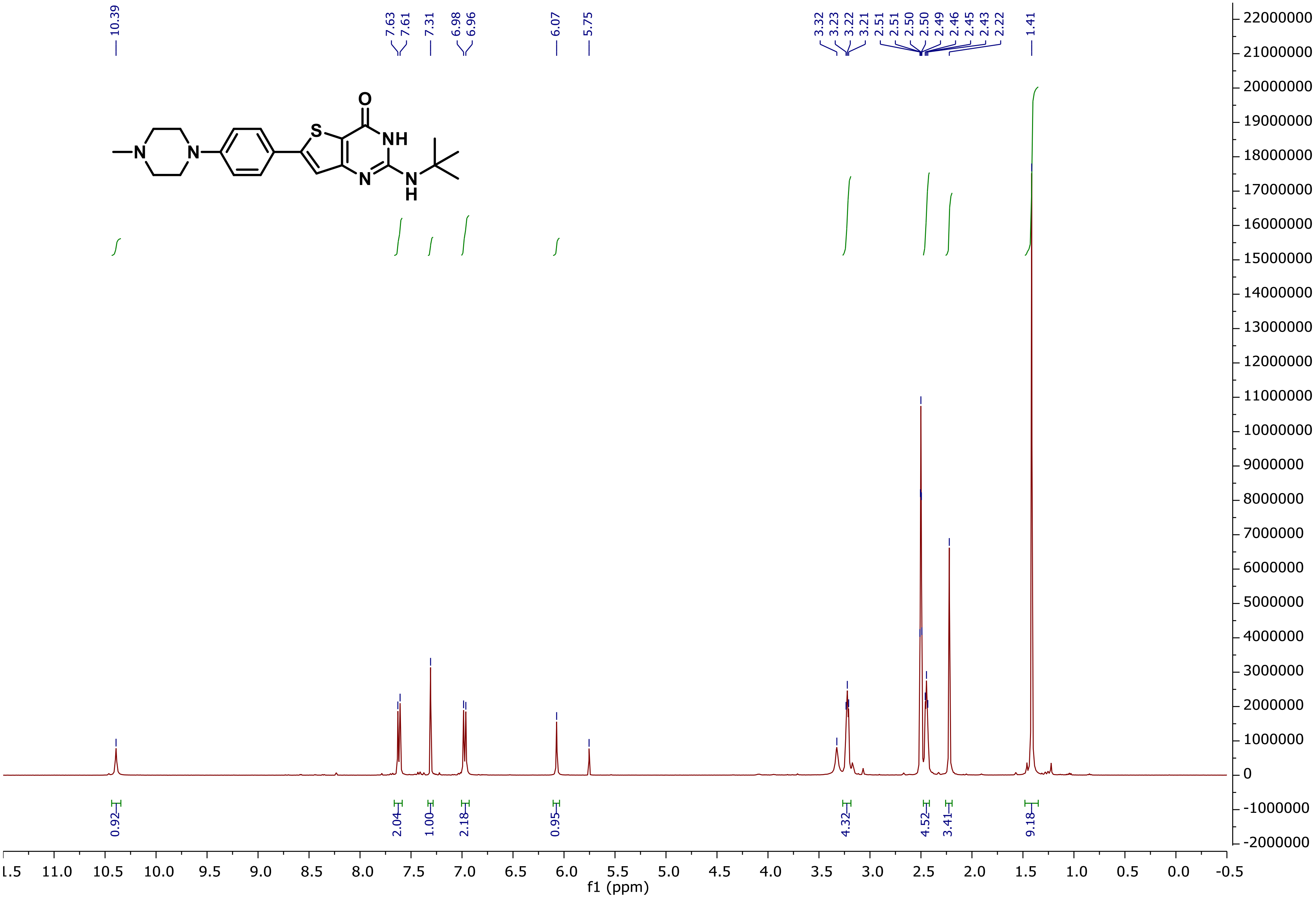
10.39
7.63
7.61
7.31
6.98
6.96
6.07
5.75
3.32
3.23
3.22
3.21
2.51
2.51
2.50
2.50
2.49
2.46
2.45
2.43
2.22
1.41
0.92
2.04
1.00
2.18
0.95
4.32
4.52
3.41
9.18
f1 (ppm)

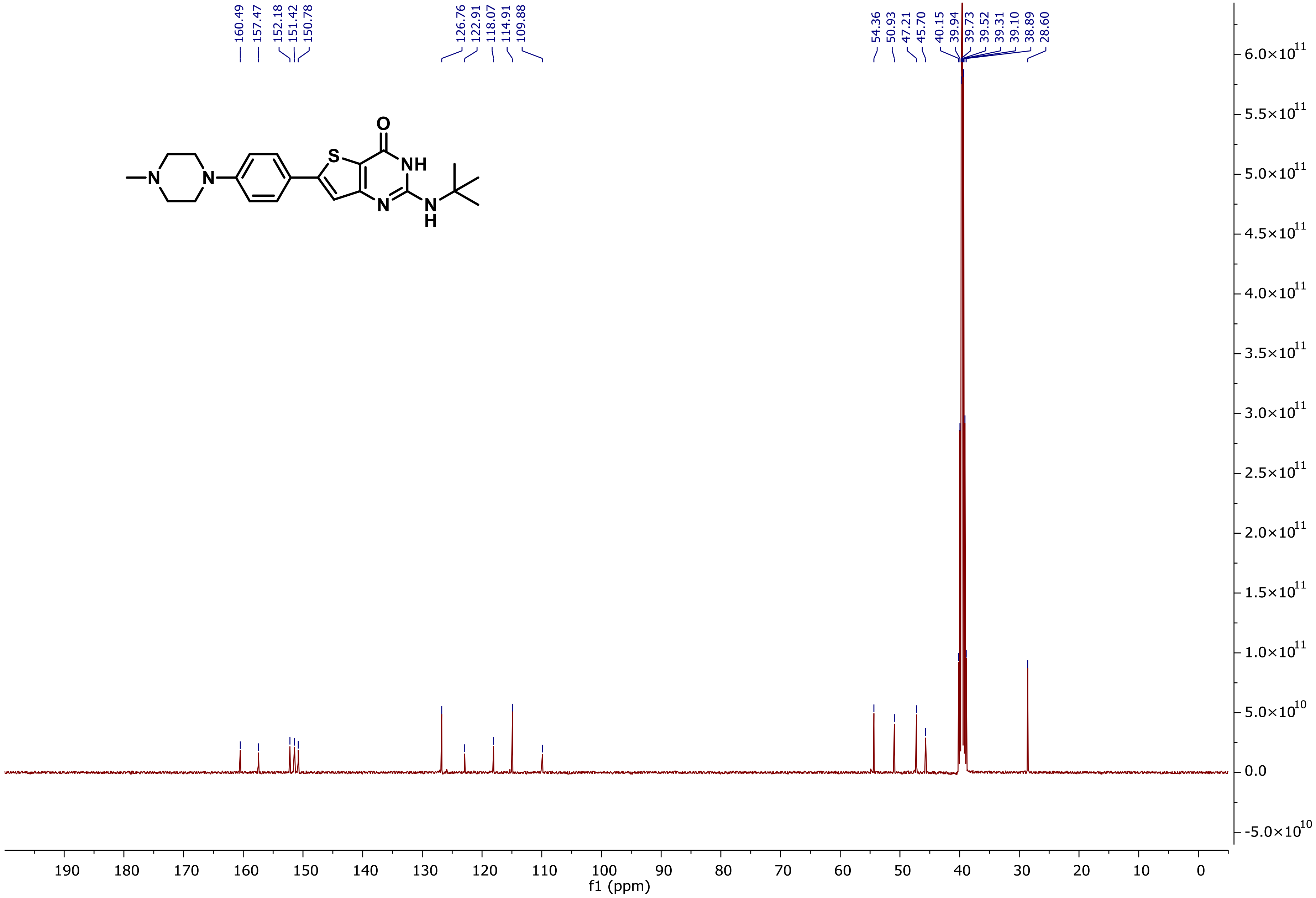
160.49
157.47
152.18
151.42
150.78
126.76
122.91
118.07
114.91
109.88
54.36
50.93
47.21
45.70
40.15
39.94
39.73
39.52
39.31
39.10
38.89
28.60
6.0×10^11
5.5×10^11
5.0×10^11
4.5×10^11
4.0×10^11
3.5×10^11
3.0×10^11
2.5×10^11
2.0×10^11
1.5×10^11
1.0×10^11
5.0×10^10
0.0
-5.0×10^10
190 180 170 160 150 140 130 120 110 100 90 80 70 60 50 40 30 20 10 0
f1 (ppm)

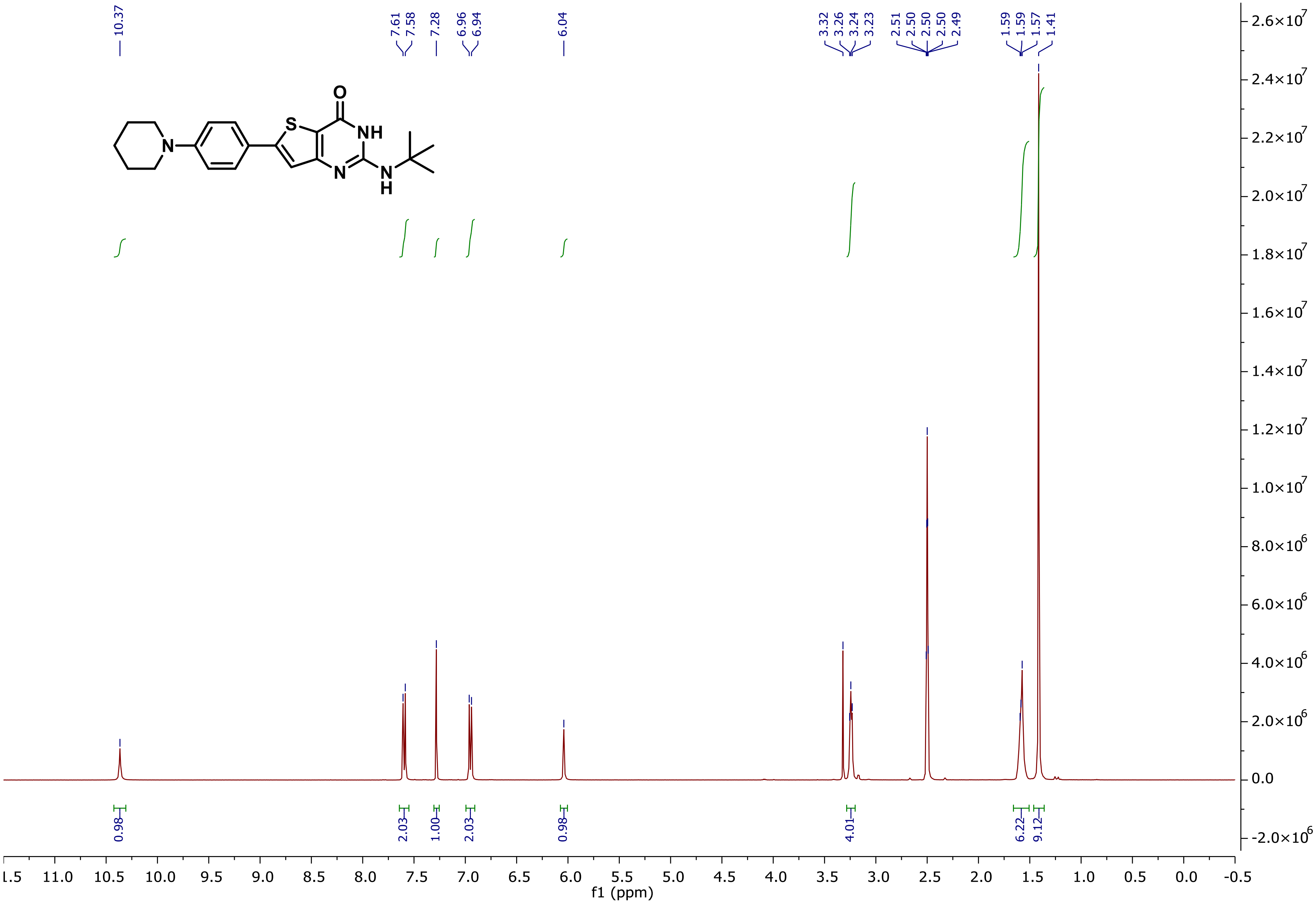

10.37
7.61
7.58
7.28
6.96
6.94
6.04
3.32
3.26
3.24
3.23
2.51
2.50
2.50
2.50
2.49
1.59
1.59
1.57
1.41
O
S
NH
N
N
N
H
0.98
2.03
1.00
2.03
0.98
4.01
6.22
9.12
f1 (ppm)

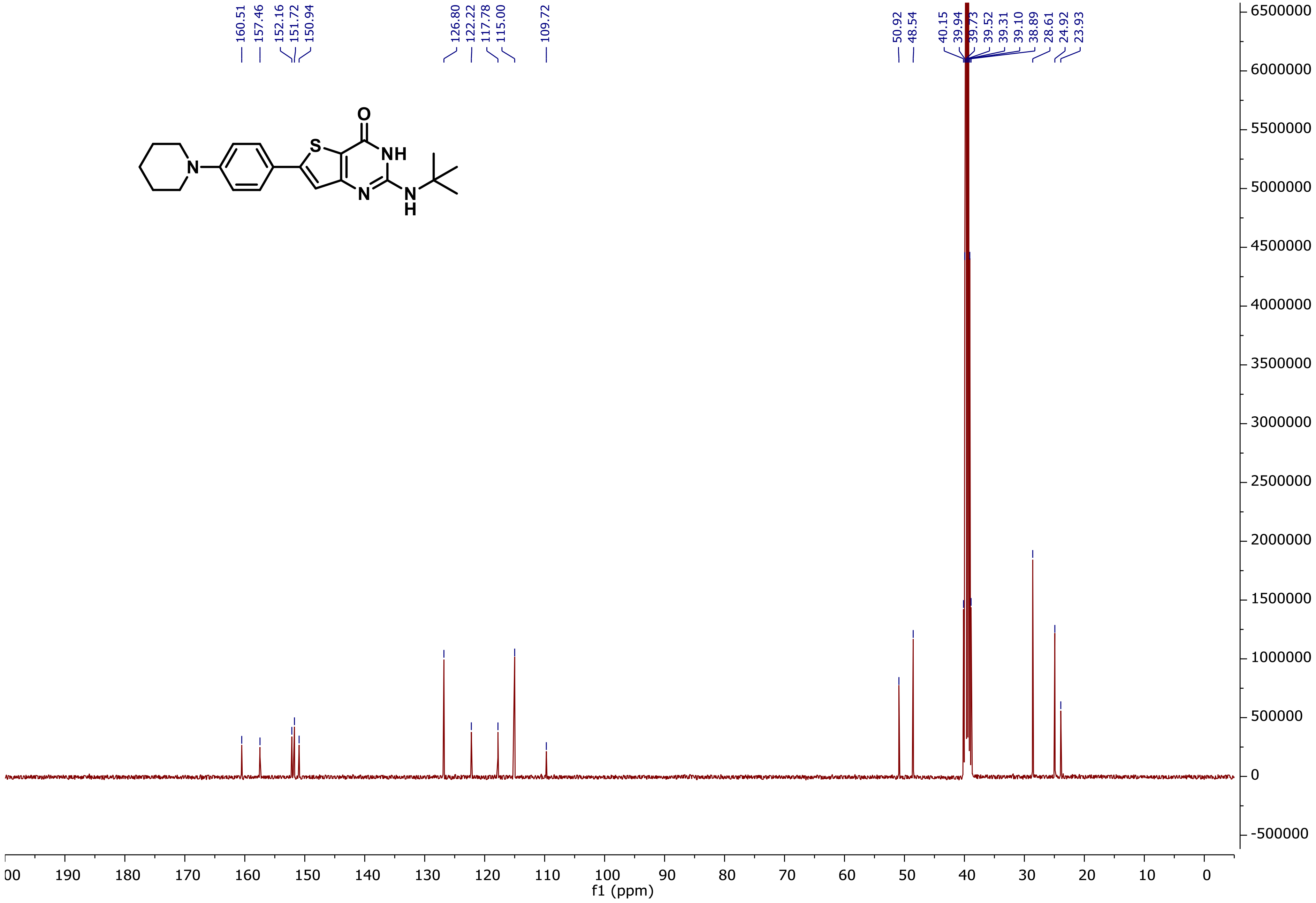

160.51
157.46
152.16
151.72
150.94
126.80
122.22
117.78
115.00
109.72
50.92
48.54
40.15
39.94
39.73
39.52
39.31
39.10
38.89
28.61
24.92
23.93
O
S
NH
N
N
H
f1 (ppm)

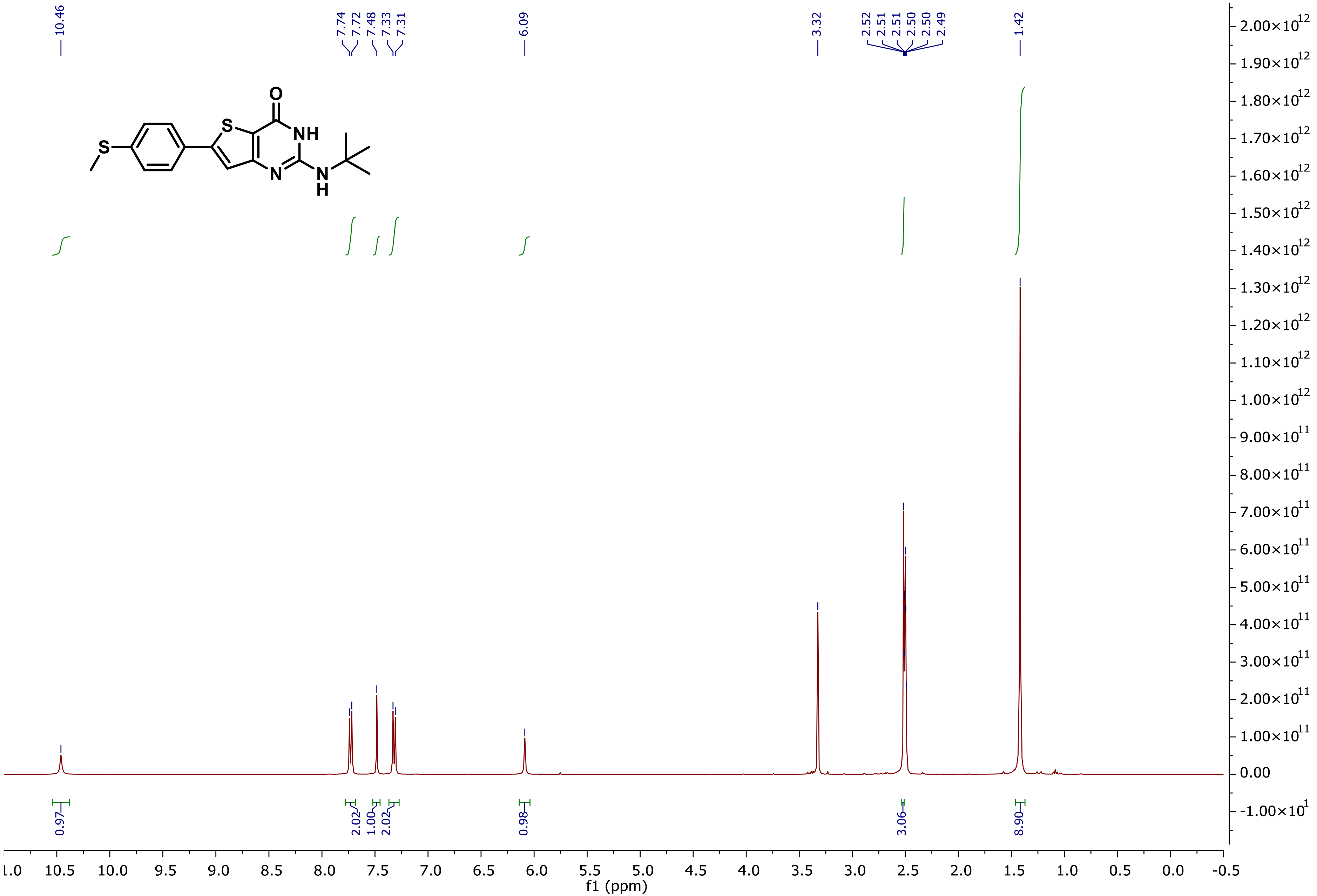

10.46
7.74
7.72
7.48
7.33
7.31
6.09
3.32
2.52
2.51
2.51
2.50
2.50
2.49
1.42
0.97
2.02
1.00
2.02
0.98
3.06
8.90
f1 (ppm)

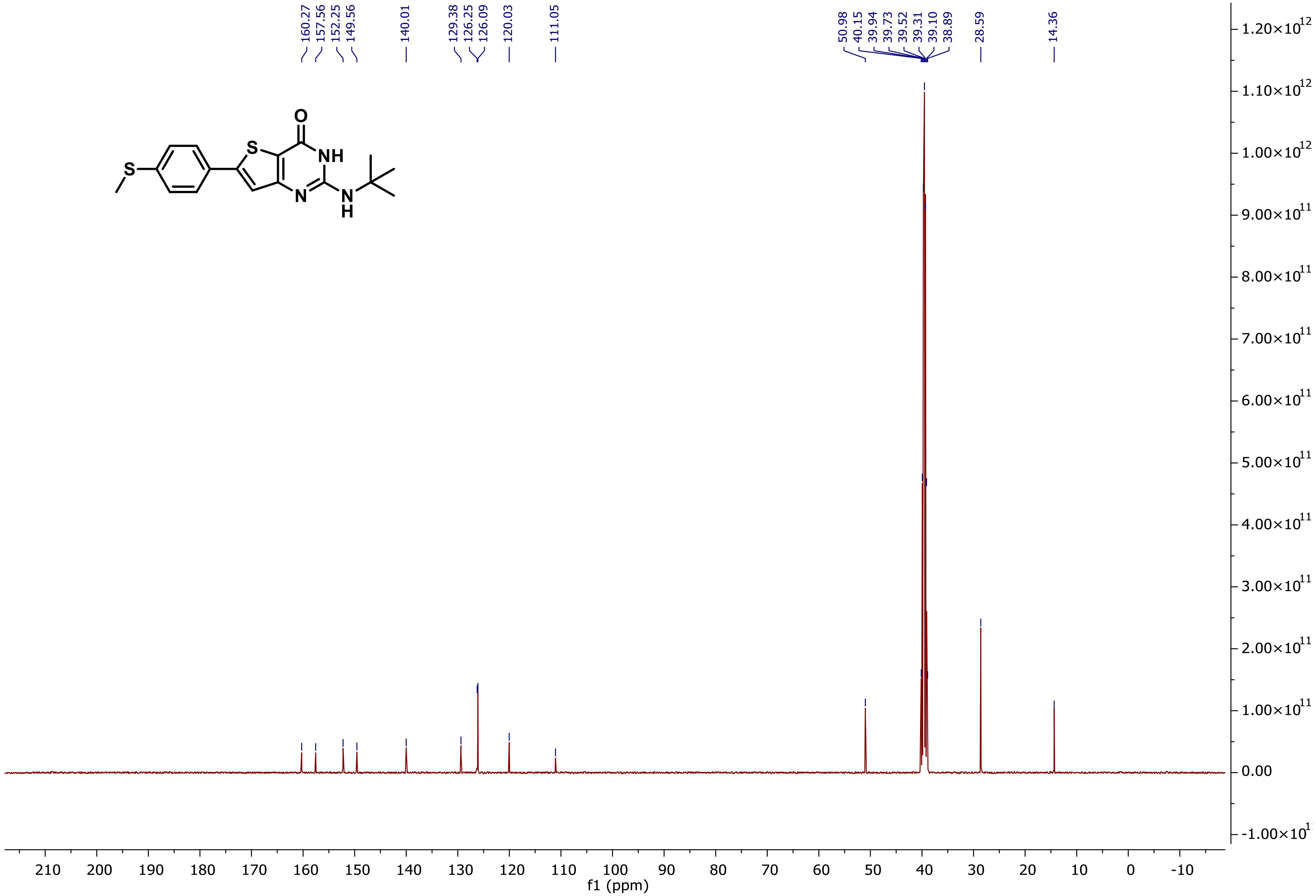
160.27
157.56
152.25
149.56
140.01
129.38
126.25
126.09
120.03
111.05
50.98
40.15
39.94
39.73
39.52
39.31
39.10
38.89
28.59
14.36
O
S
NH
N
H
S
f1 (ppm)
210 200 190 180 170 160 150 140 130 120 110 100 90 80 70 60 50 40 30 20 10 0 -10
1.20×10^12
1.10×10^12
1.00×10^12
9.00×10^11
8.00×10^11
7.00×10^11
6.00×10^11
5.00×10^11
4.00×10^11
3.00×10^11
2.00×10^11
1.00×10^11
0.00
-1.00×10^1

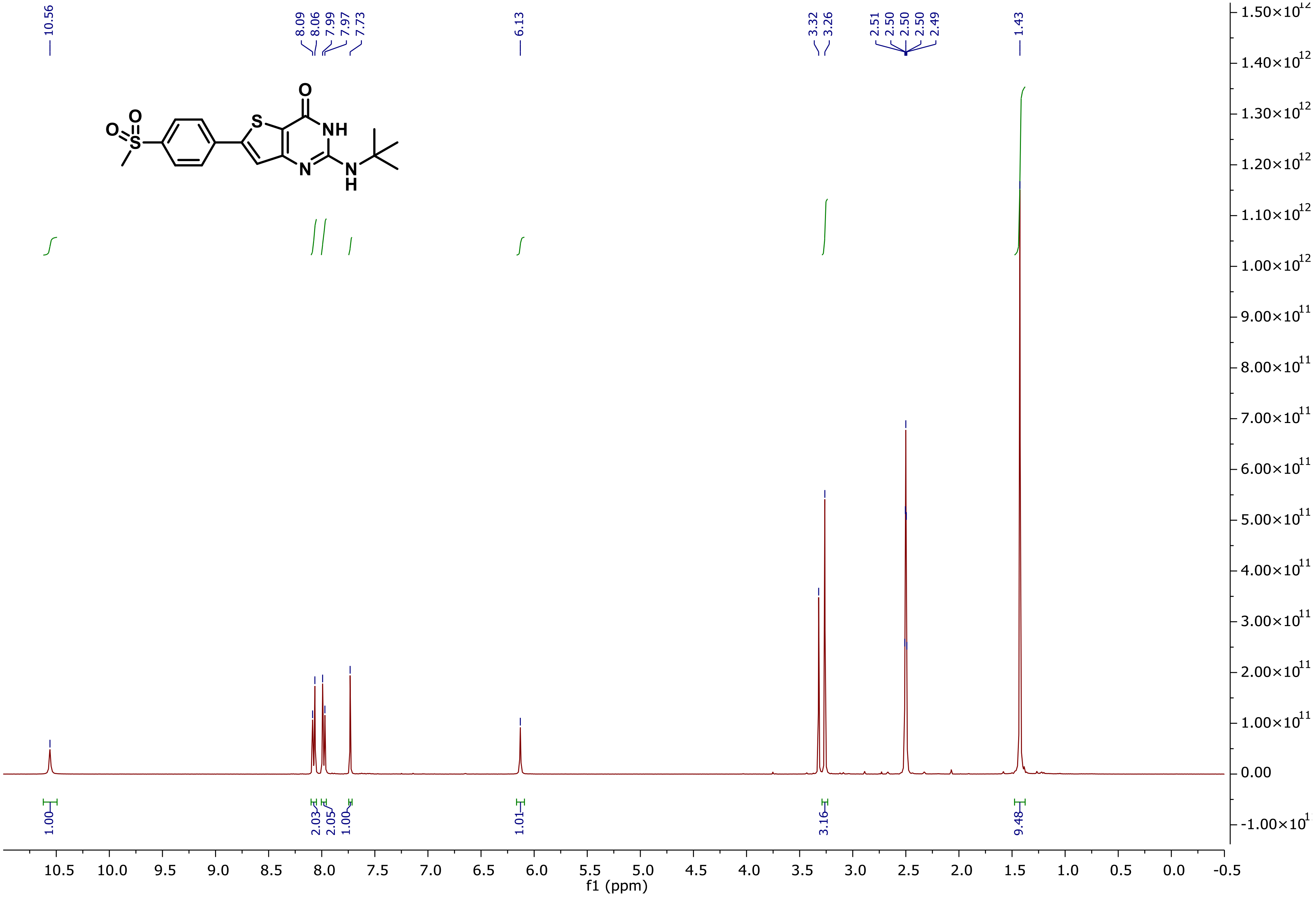
10.56
8.09
8.06
7.99
7.97
7.73
6.13
3.32
3.26
2.51
2.50
2.50
2.50
2.49
1.43
1.00
2.03
2.05
1.00
1.01
3.16
9.48
f1 (ppm)

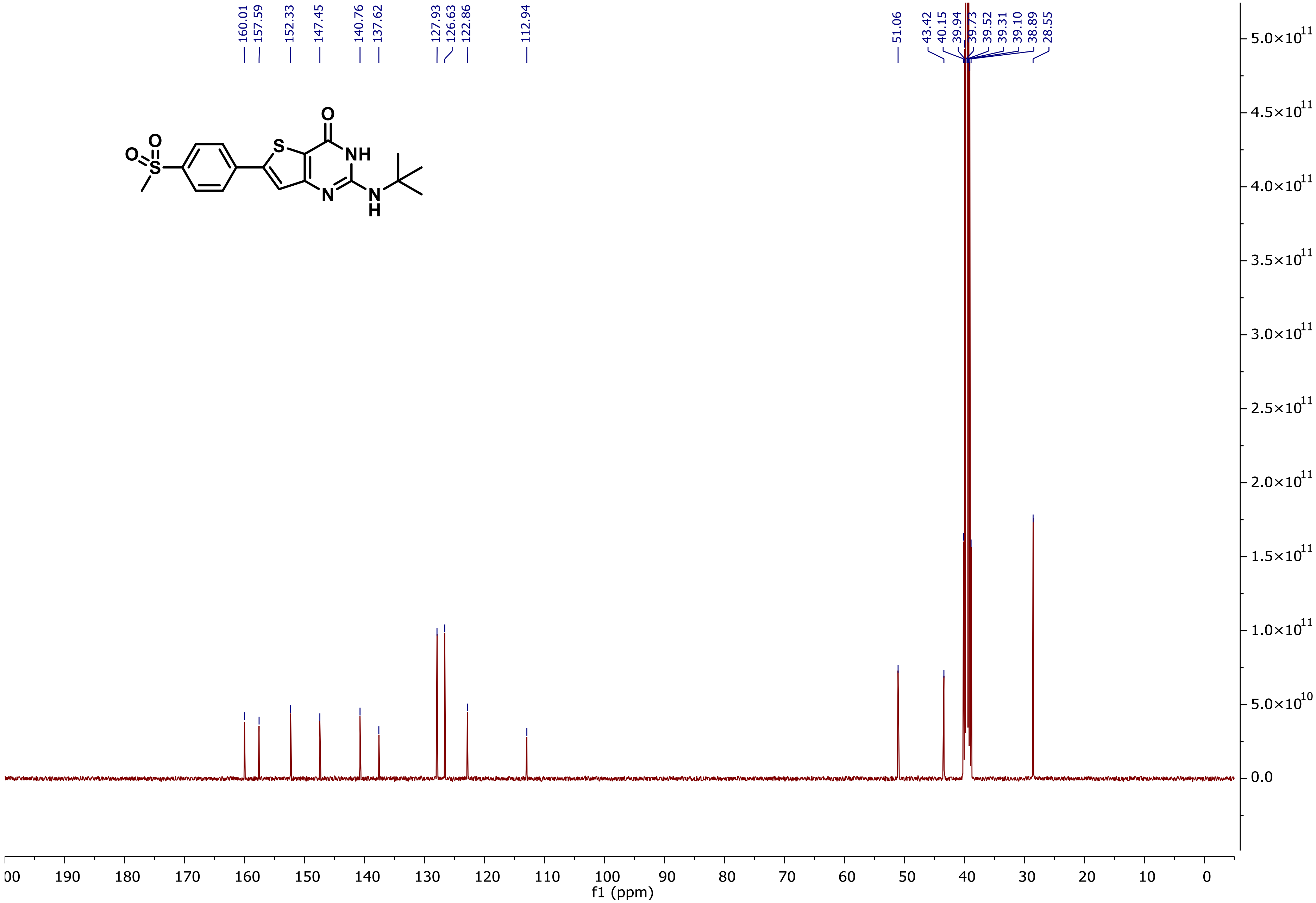
160.01
157.59
152.33
147.45
140.76
137.62
127.93
126.63
122.86
112.94
51.06
43.42
40.15
39.94
39.73
39.52
39.31
39.10
38.89
28.55
O
S
NH
N
H
5.0×10^11
4.5×10^11
4.0×10^11
3.5×10^11
3.0×10^11
2.5×10^11
2.0×10^11
1.5×10^11
1.0×10^11
5.0×10^10
0.0
00
190
180
170
160
150
140
130
120
110
100
90
80
70
60
50
40
30
20
10
0
f1 (ppm)

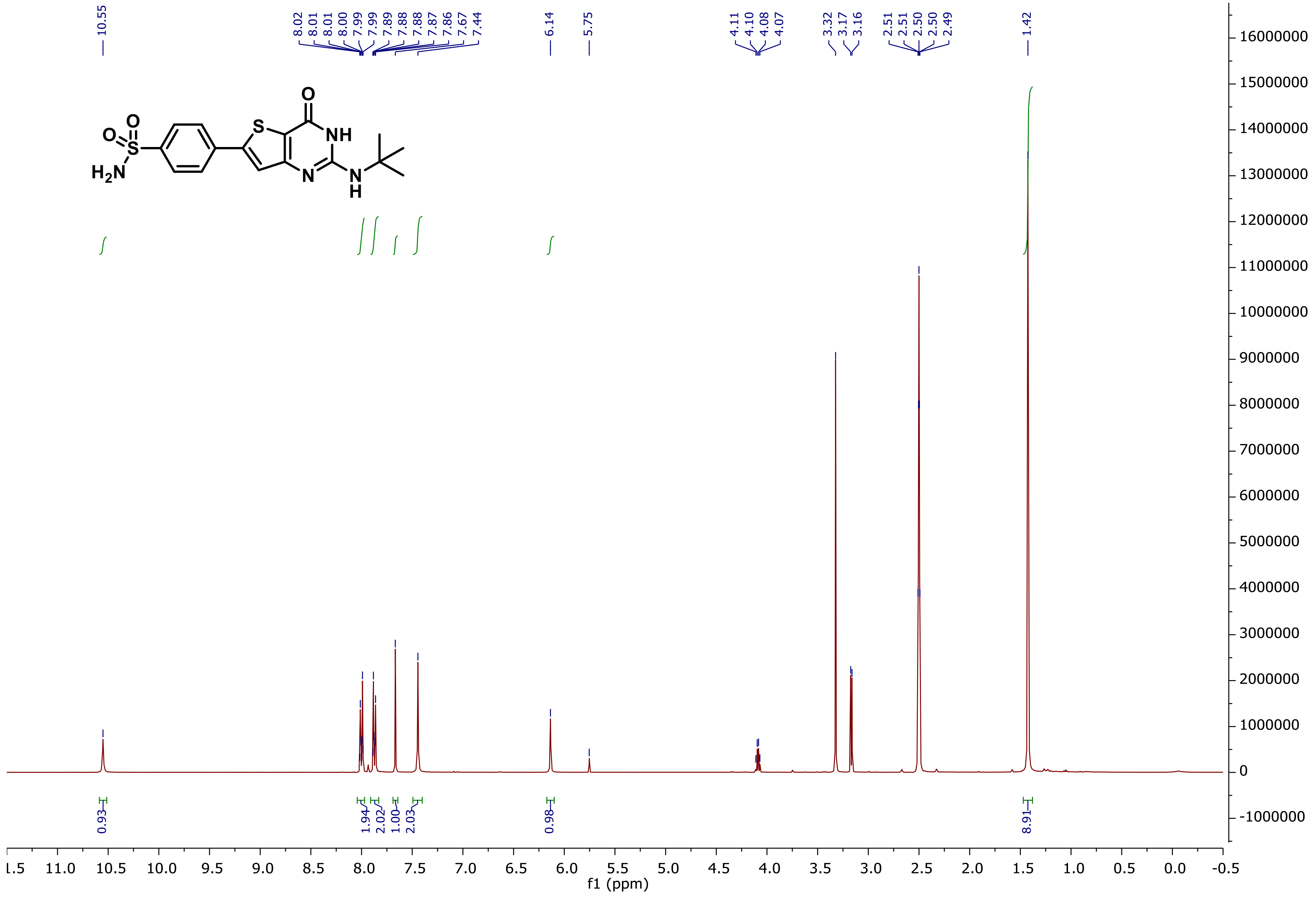
10.55
8.02
8.01
8.01
8.00
7.99
7.99
7.89
7.88
7.88
7.87
7.86
7.67
7.44
6.14
5.75
4.11
4.10
4.08
4.07
3.32
3.17
3.16
2.51
2.51
2.50
2.50
2.49
1.42
O
S
NH
O
O
S
H_2N
N
N
H
0.93
1.94
2.02
1.00
2.03
0.98
8.91
f1 (ppm)

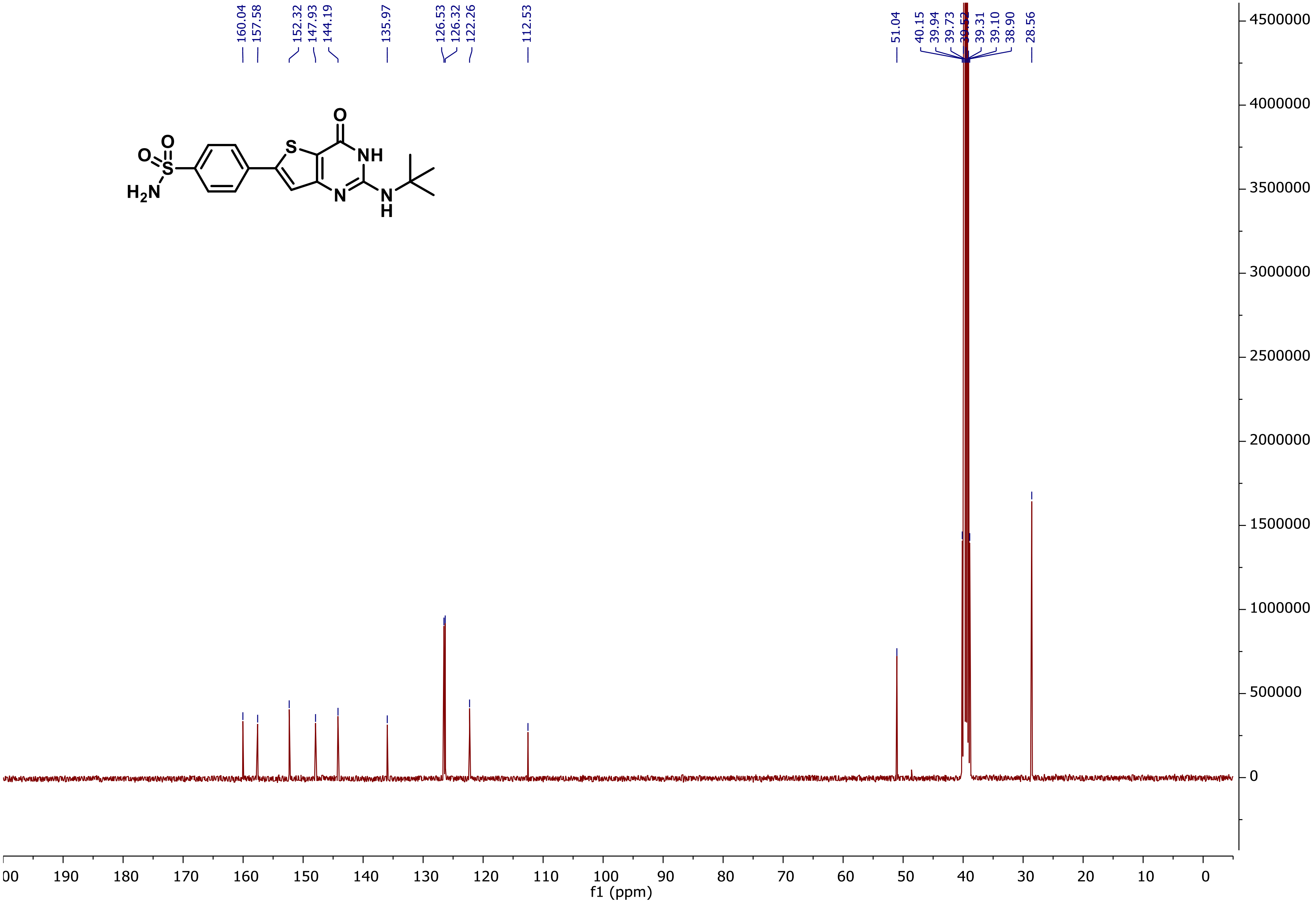

160.04
157.58
152.32
147.93
144.19
135.97
126.53
126.32
122.26
112.53
51.04
40.15
39.94
39.73
39.52
39.31
39.10
38.90
28.56
O
S
NH
O
O
S
H2N
N
N
H
4500000
4000000
3500000
3000000
2500000
2000000
1500000
1000000
500000
0
190
180
170
160
150
140
130
120
110
100
90
80
70
60
50
40
30
20
10
0
f1 (ppm)

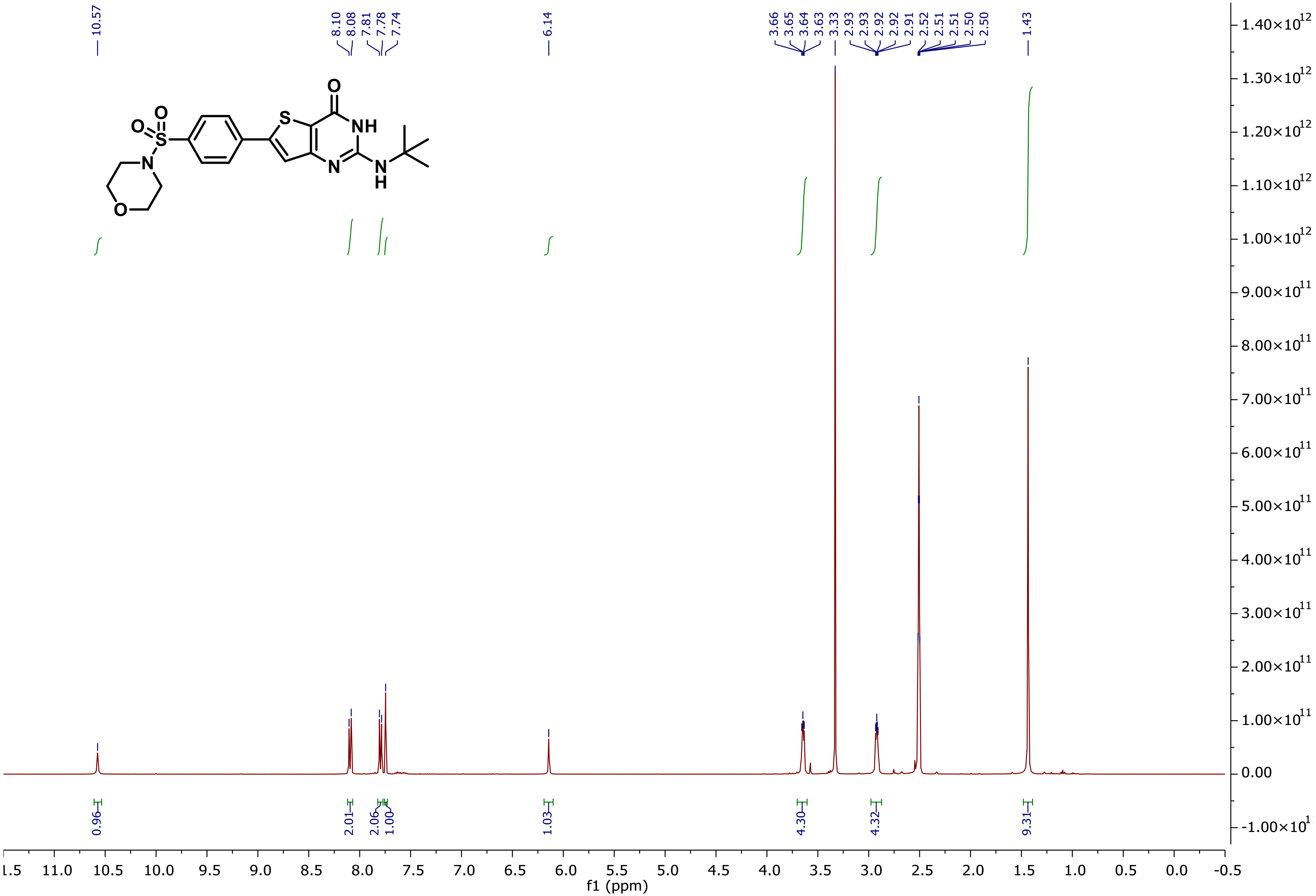

10.57
8.10
8.08
7.81
7.78
7.74
6.14
3.66
3.65
3.64
3.63
3.33
2.93
2.93
2.92
2.92
2.91
2.52
2.51
2.51
2.50
2.50
1.43
0.96
2.01
2.06
1.00
1.03
4.30
4.32
9.31
f1 (ppm)

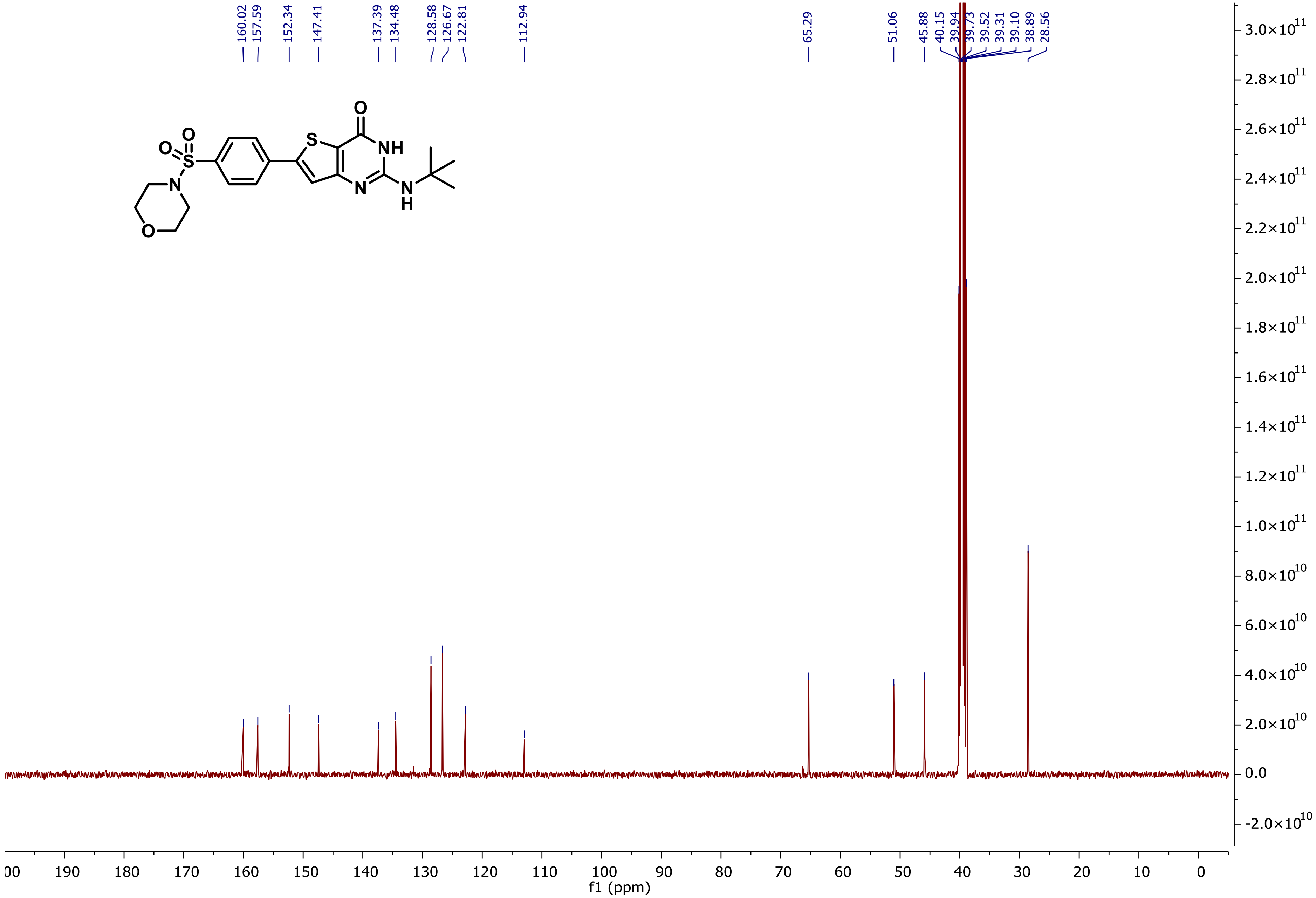

160.02
157.59
152.34
147.41
137.39
134.48
128.58
126.67
122.81
112.94
65.29
51.06
45.88
40.15
39.94
39.73
39.52
39.31
39.10
38.89
28.56
f1 (ppm)

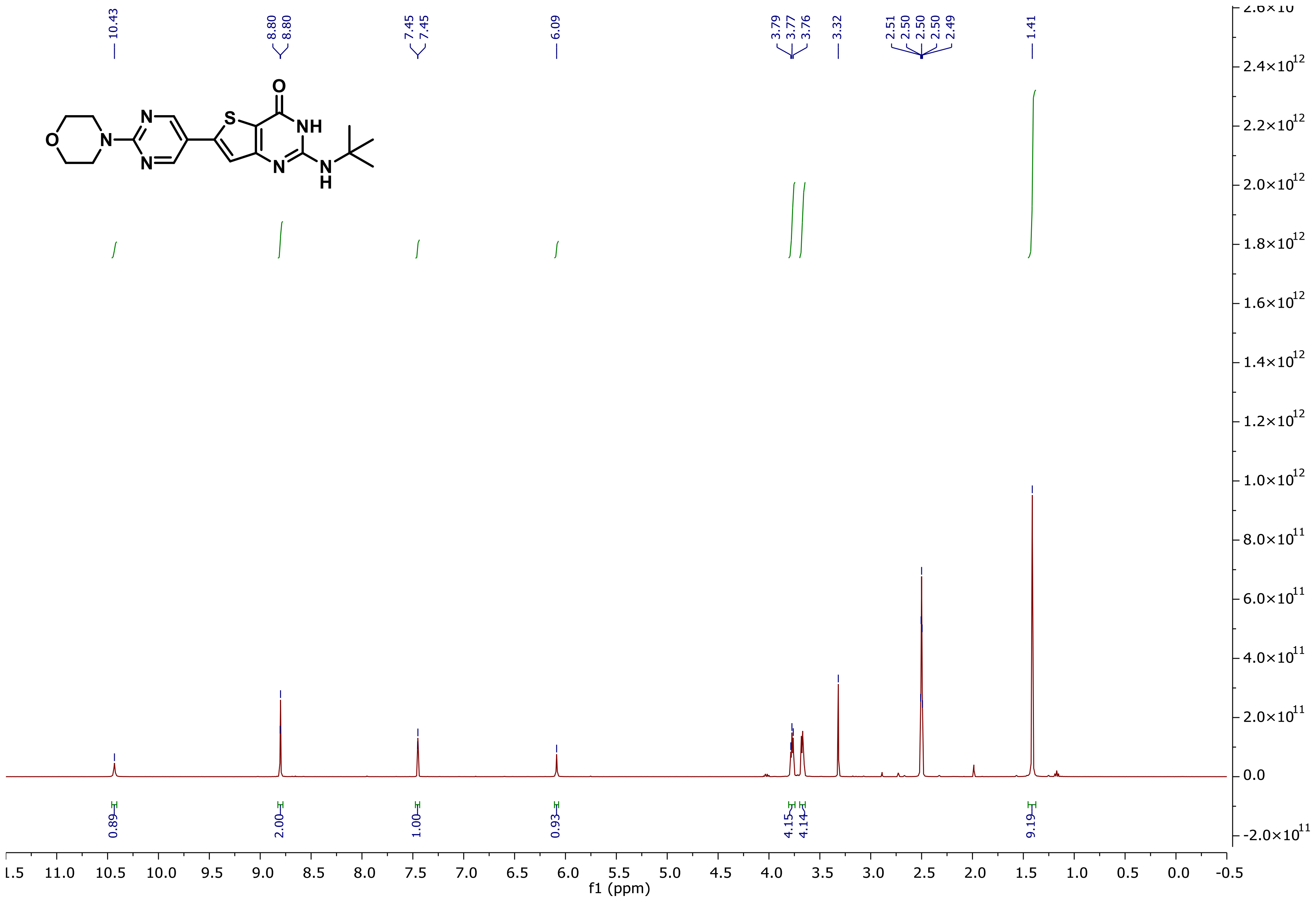
10.43
8.80
8.80
7.45
7.45
6.09
3.79
3.77
3.76
3.32
2.51
2.50
2.50
2.50
2.49
1.41
O
S
NH
N
N
N
N
N
O
H
0.89
2.00
1.00
0.93
4.15
4.14
9.19
f1 (ppm)

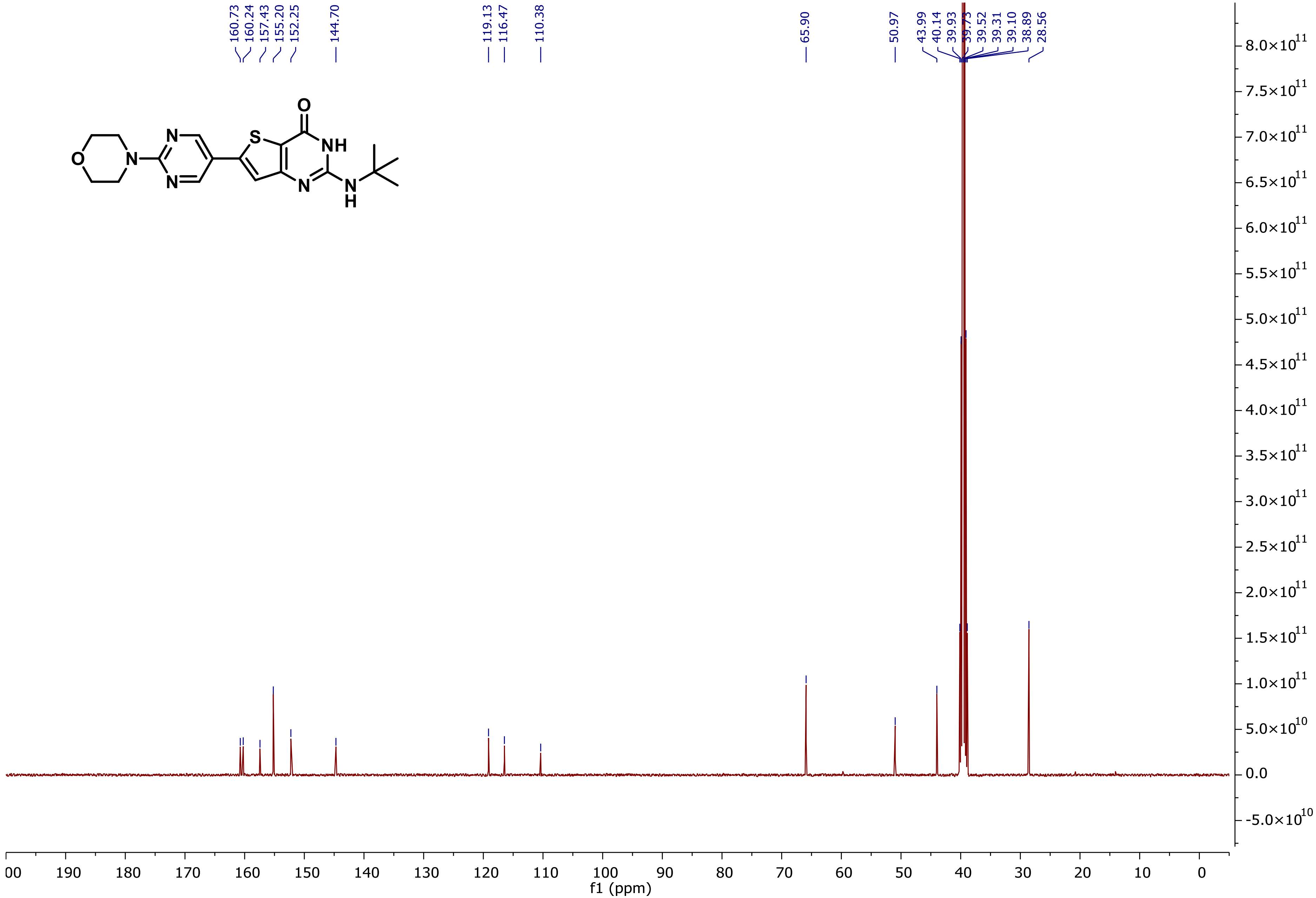

160.73
160.24
157.43
155.20
152.25
144.70
119.13
116.47
110.38
65.90
50.97
43.99
40.14
39.93
39.73
39.52
39.31
39.10
38.89
28.56
f1 (ppm)

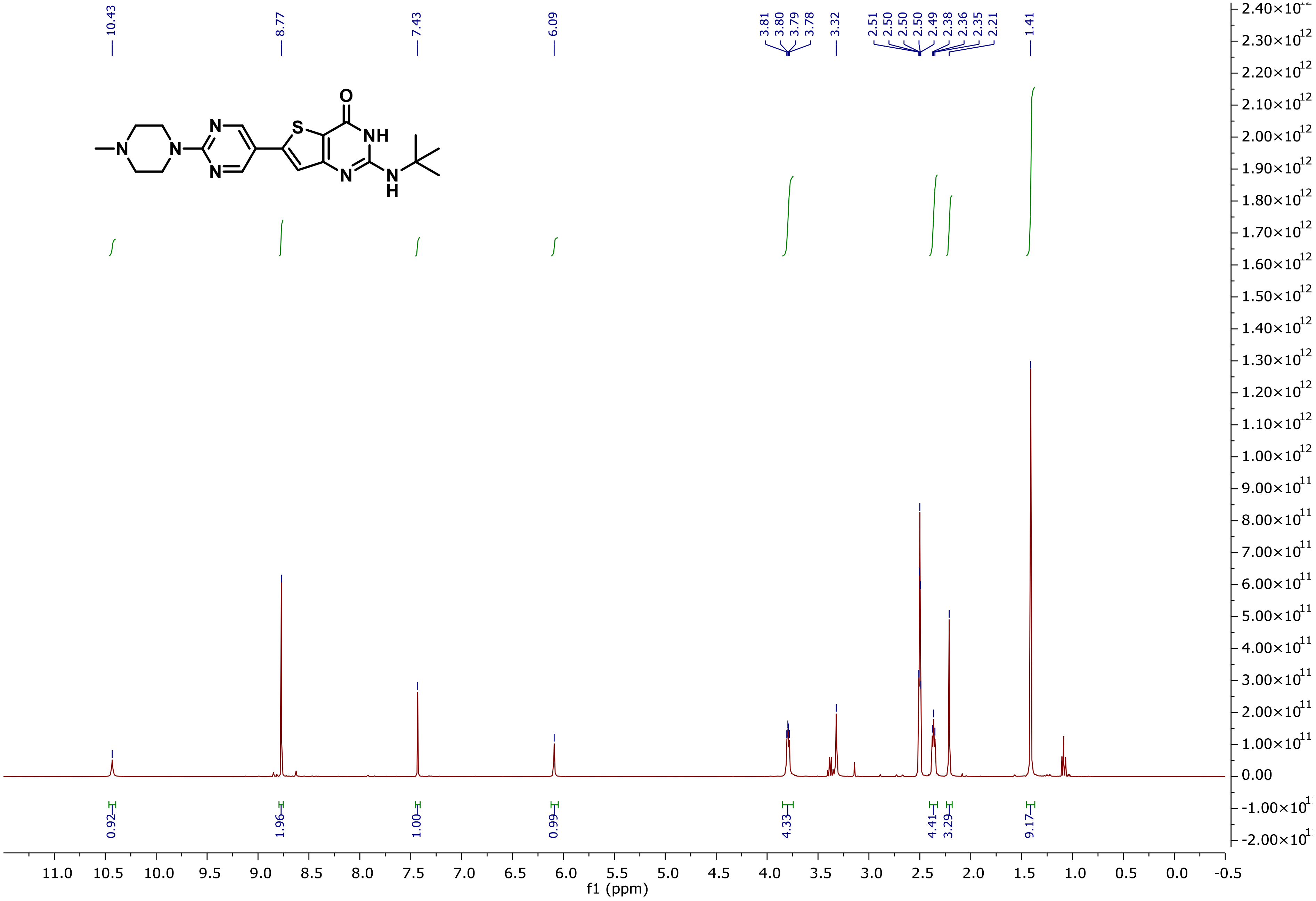
10.43
8.77
7.43
6.09
3.81
3.80
3.79
3.78
3.32
2.51
2.50
2.50
2.50
2.49
2.38
2.36
2.35
2.21
1.41
0.92
1.96
1.00
0.99
4.33
4.41
3.29
9.17
f1 (ppm)

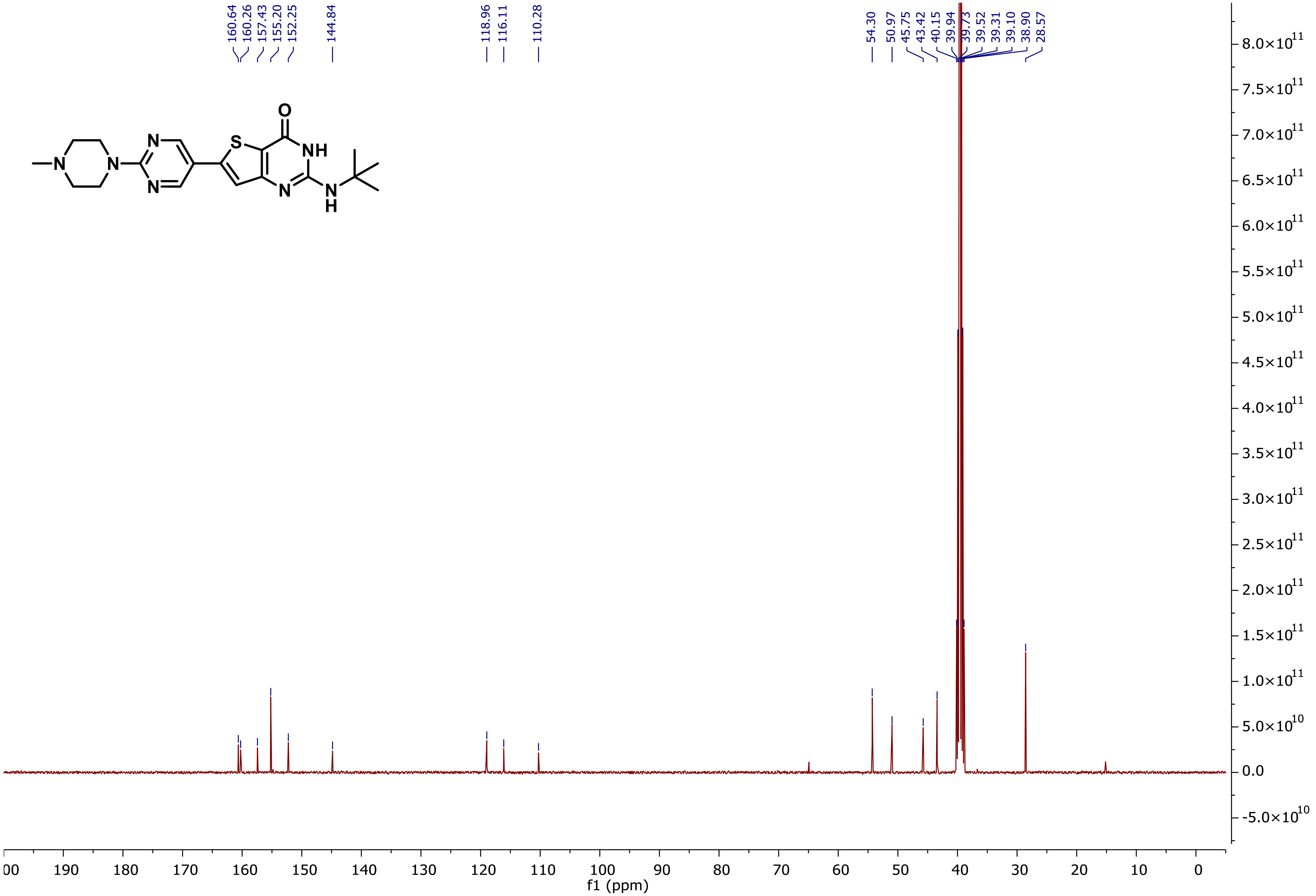

160.64
160.26
157.43
155.20
152.25
144.84
118.96
116.11
110.28
54.30
50.97
45.75
43.42
40.15
39.94
39.73
39.52
39.31
39.10
38.90
28.57
f1 (ppm)

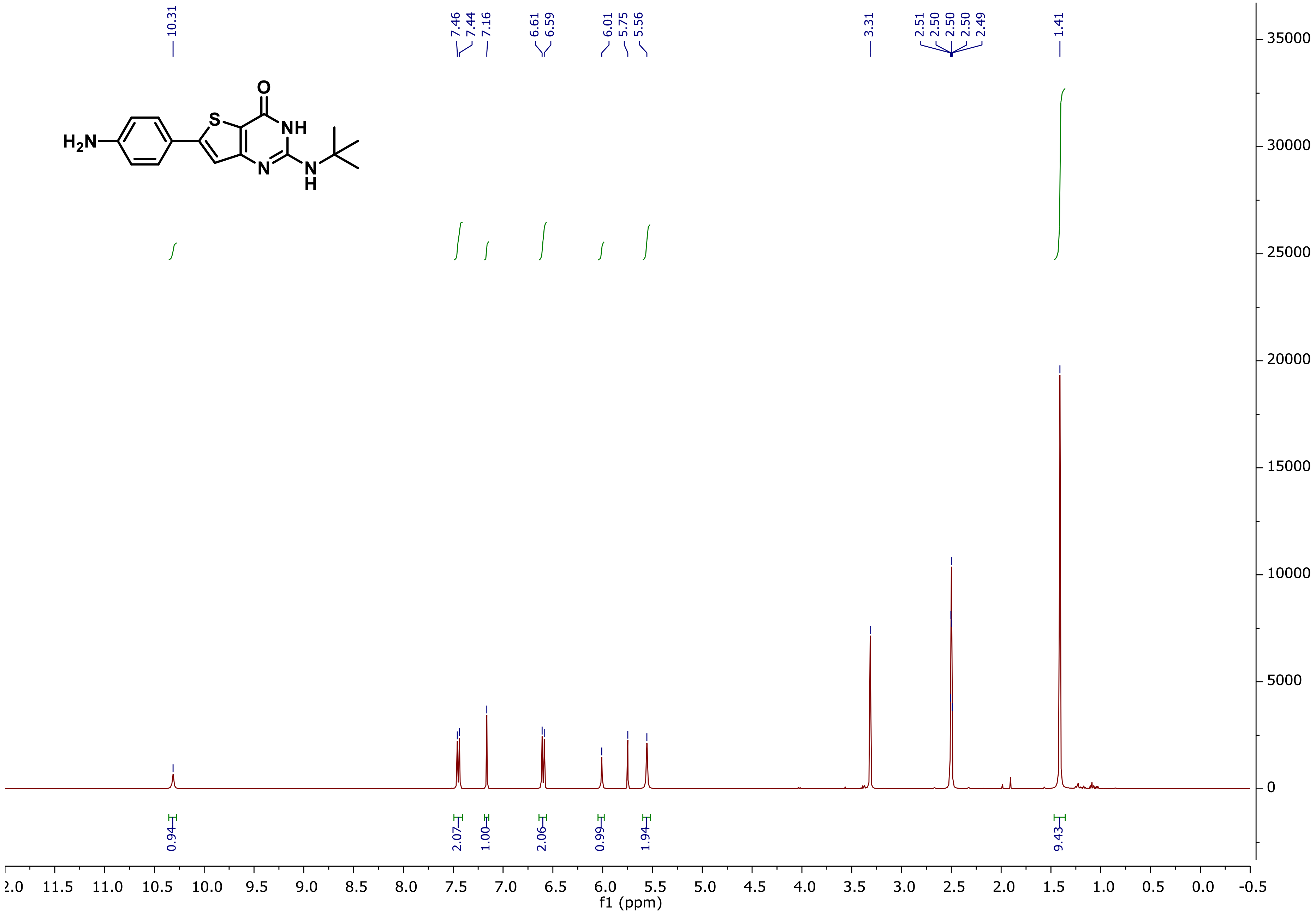
10.31
7.46
7.44
7.16
6.61
6.59
6.01
5.75
5.56
3.31
2.51
2.50
2.50
2.50
2.49
1.41
H2N
S
O
NH
N
N
H
0.94
2.07
1.00
2.06
0.99
1.94
9.43
35000
30000
25000
20000
15000
10000
5000
0
2.0 11.5 11.0 10.5 10.0 9.5 9.0 8.5 8.0 7.5 7.0 6.5 6.0 5.5 5.0 4.5 4.0 3.5 3.0 2.5 2.0 1.5 1.0 0.5 0.0 -0.5
f1 (ppm)

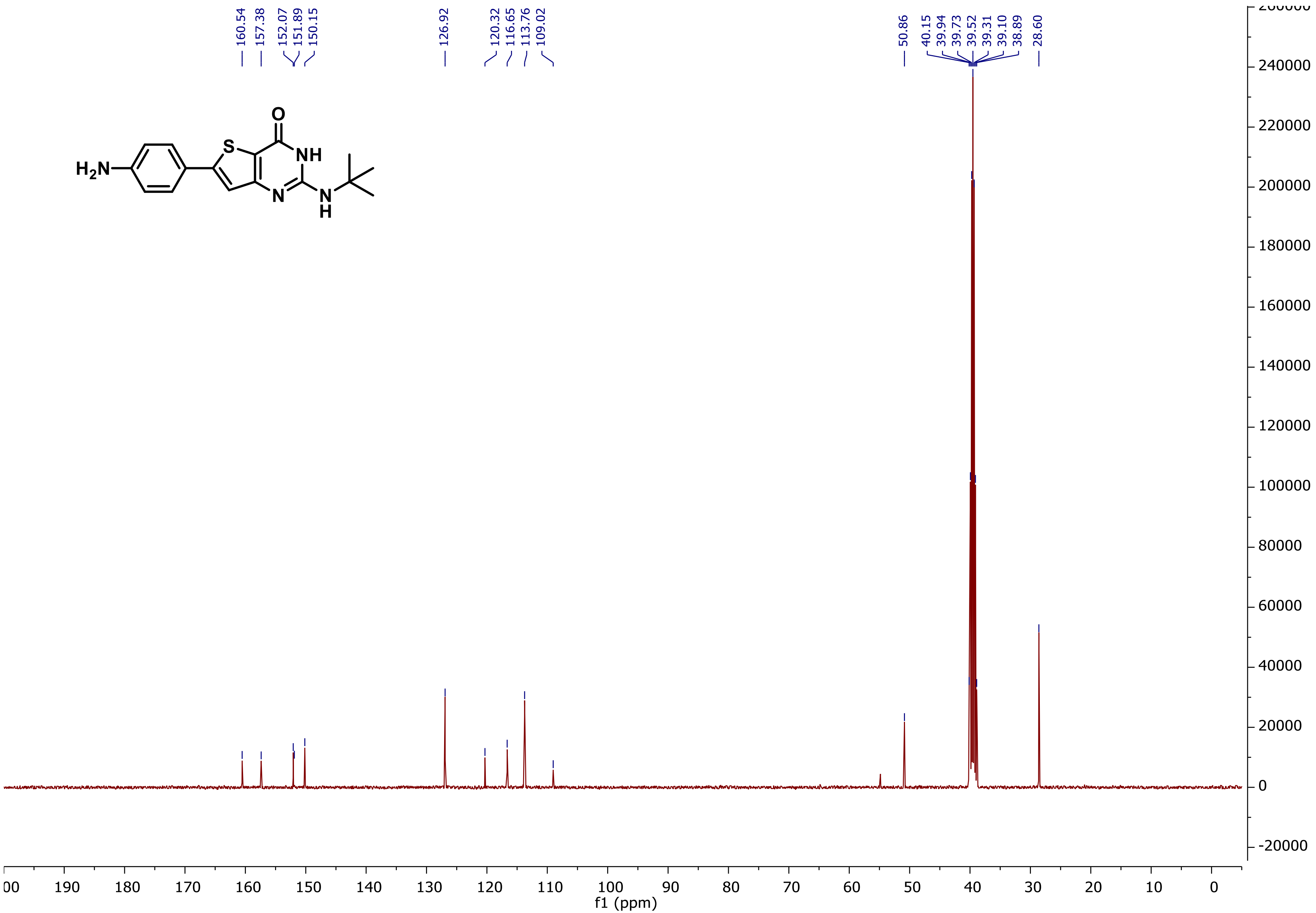

160.54
157.38
152.07
151.89
150.15
126.92
120.32
116.65
113.76
109.02
50.86
40.15
39.94
39.73
39.52
39.31
39.10
38.89
28.60
f1 (ppm)

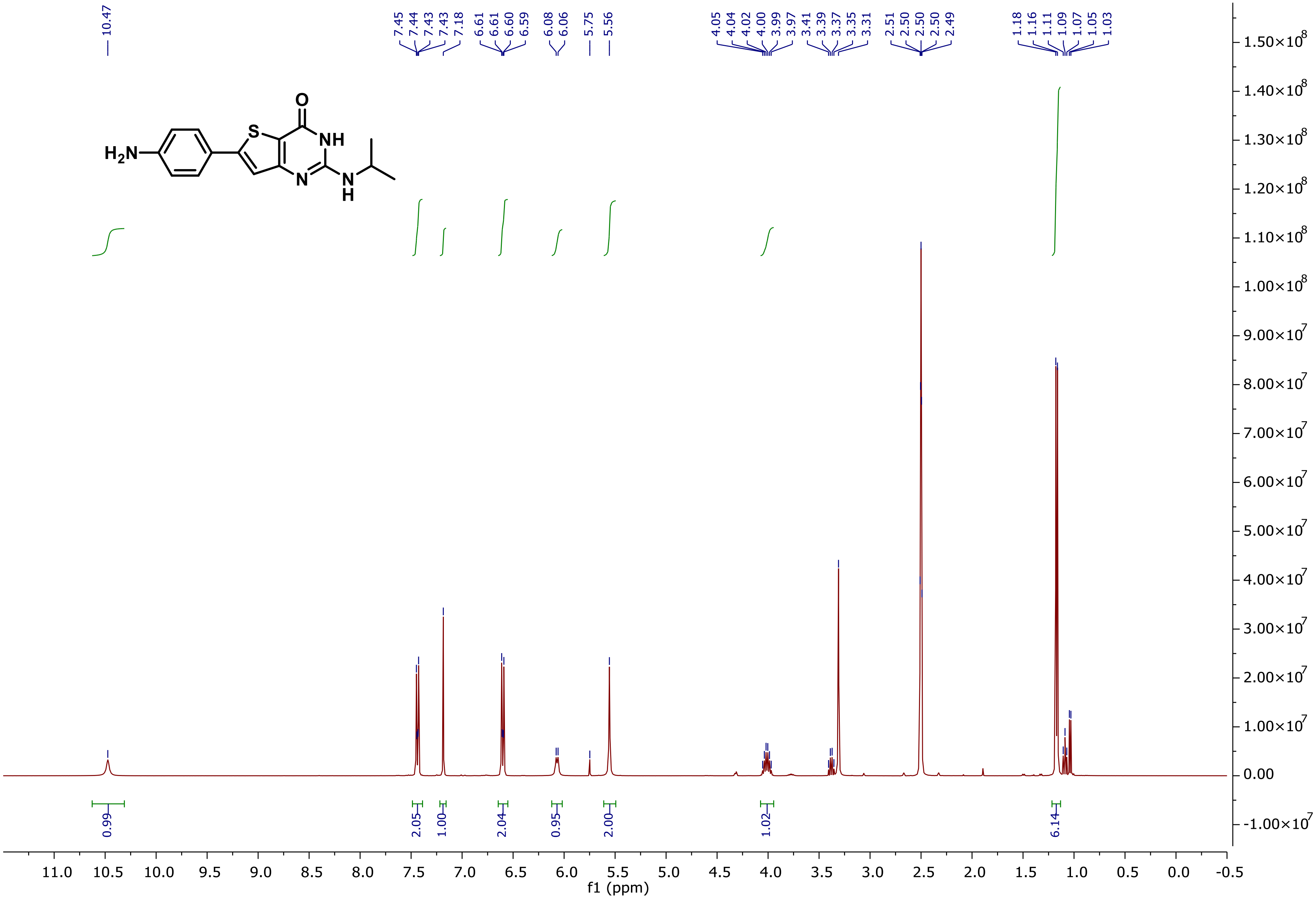

10.47
7.45
7.44
7.43
7.43
7.18
6.61
6.61
6.60
6.59
6.08
6.06
5.75
5.56
4.05
4.04
4.02
4.00
3.99
3.97
3.41
3.39
3.37
3.35
3.31
2.51
2.50
2.50
2.50
2.49
1.18
1.16
1.11
1.09
1.07
1.05
1.03
0.99
2.05
1.00
2.04
0.95
2.00
1.02
6.14
f1 (ppm)

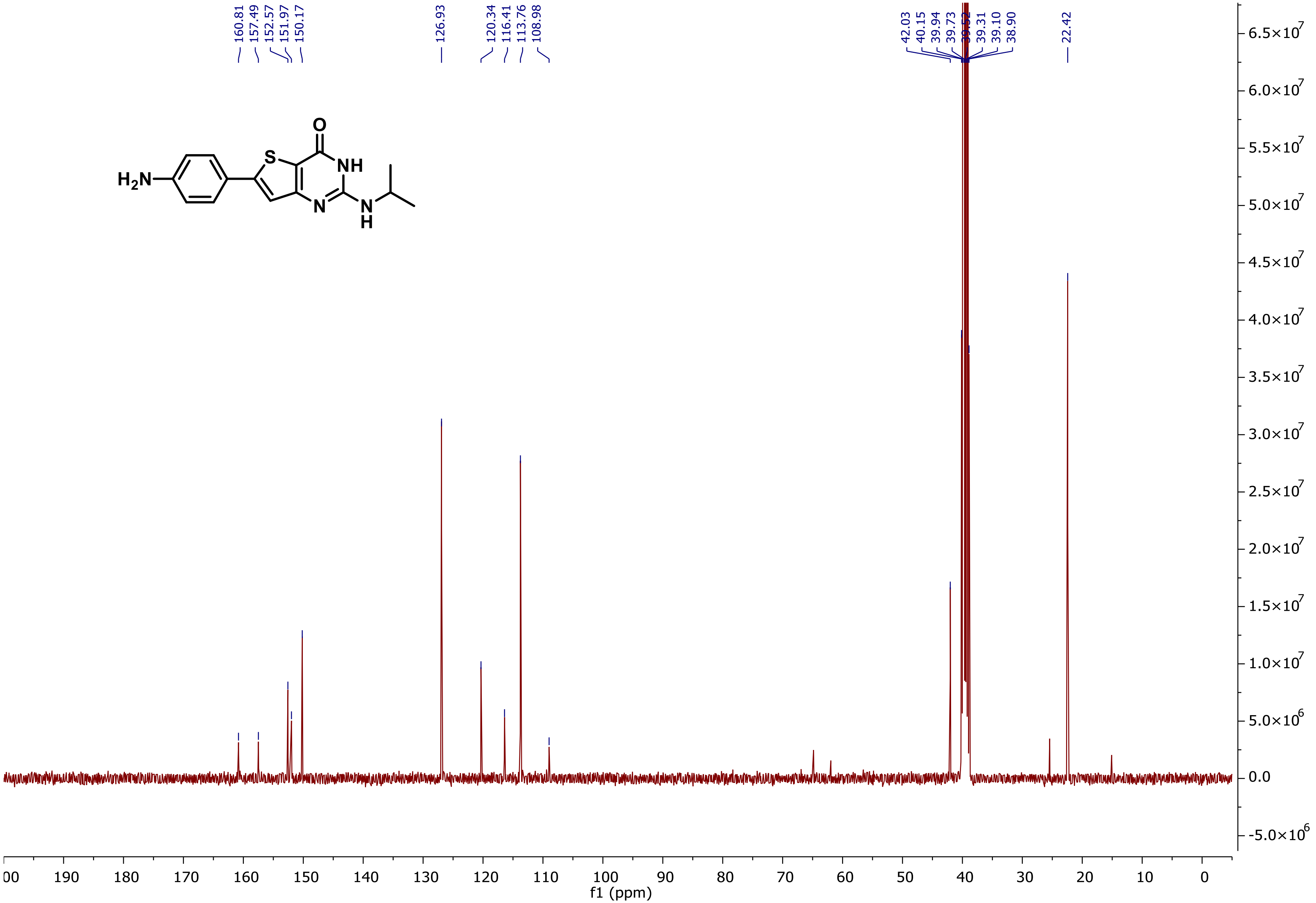
160.81
157.49
152.57
151.97
150.17
126.93
120.34
116.41
113.76
108.98
42.03
40.15
39.94
39.73
39.31
39.10
38.90
22.42
f1 (ppm)

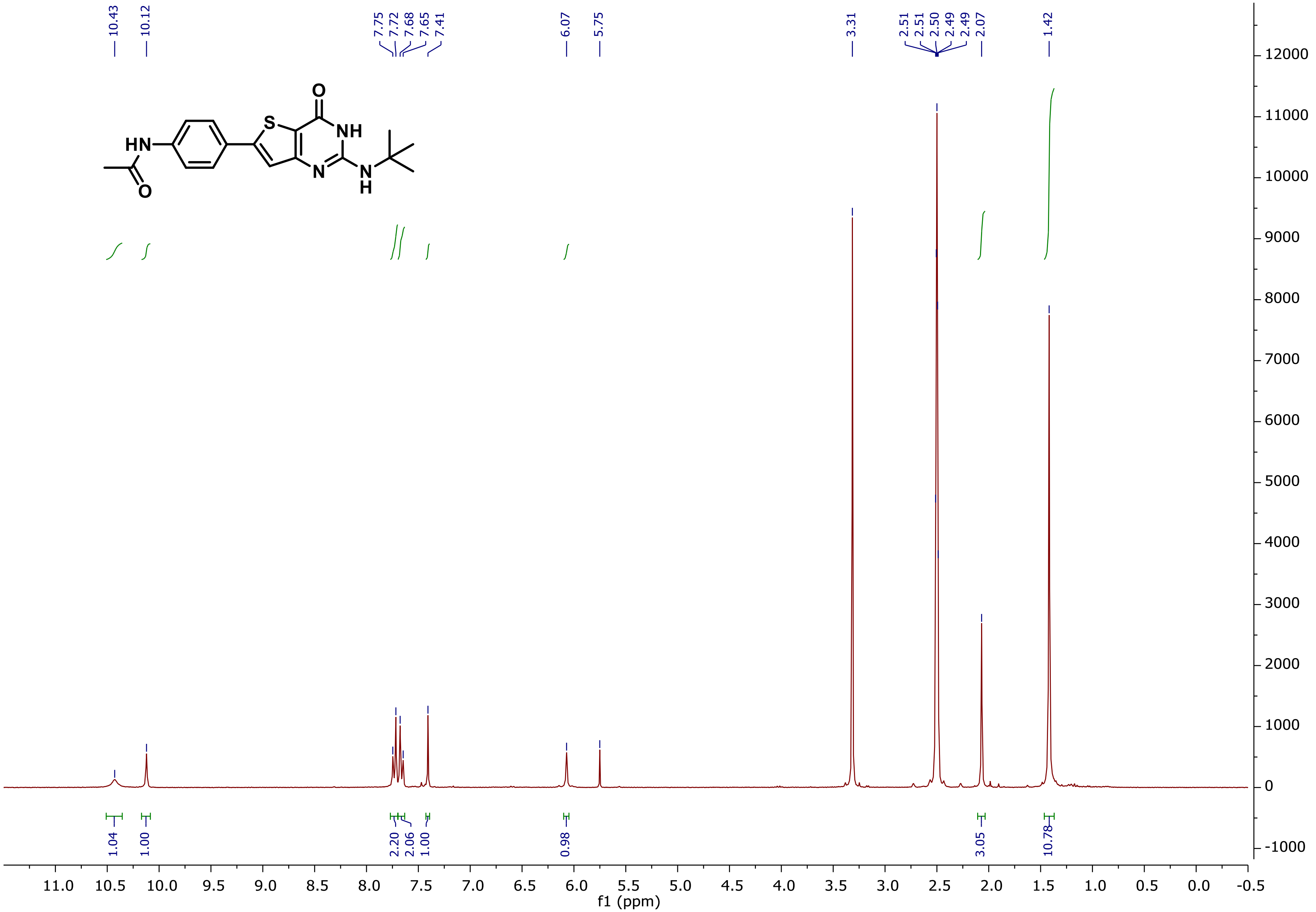

10.43
10.12
7.75
7.72
7.68
7.65
7.41
6.07
5.75
3.31
2.51
2.51
2.50
2.49
2.49
2.07
1.42
O
S
NH
HN
N
N
H
O
1.04
1.00
2.20
2.06
1.00
0.98
3.05
10.78
12000
11000
10000
9000
8000
7000
6000
5000
4000
3000
2000
1000
0
-1000
11.0
10.5
10.0
9.5
9.0
8.5
8.0
7.5
7.0
6.5
6.0
5.5
5.0
4.5
4.0
3.5
3.0
2.5
2.0
1.5
1.0
0.5
0.0
-0.5
f1 (ppm)

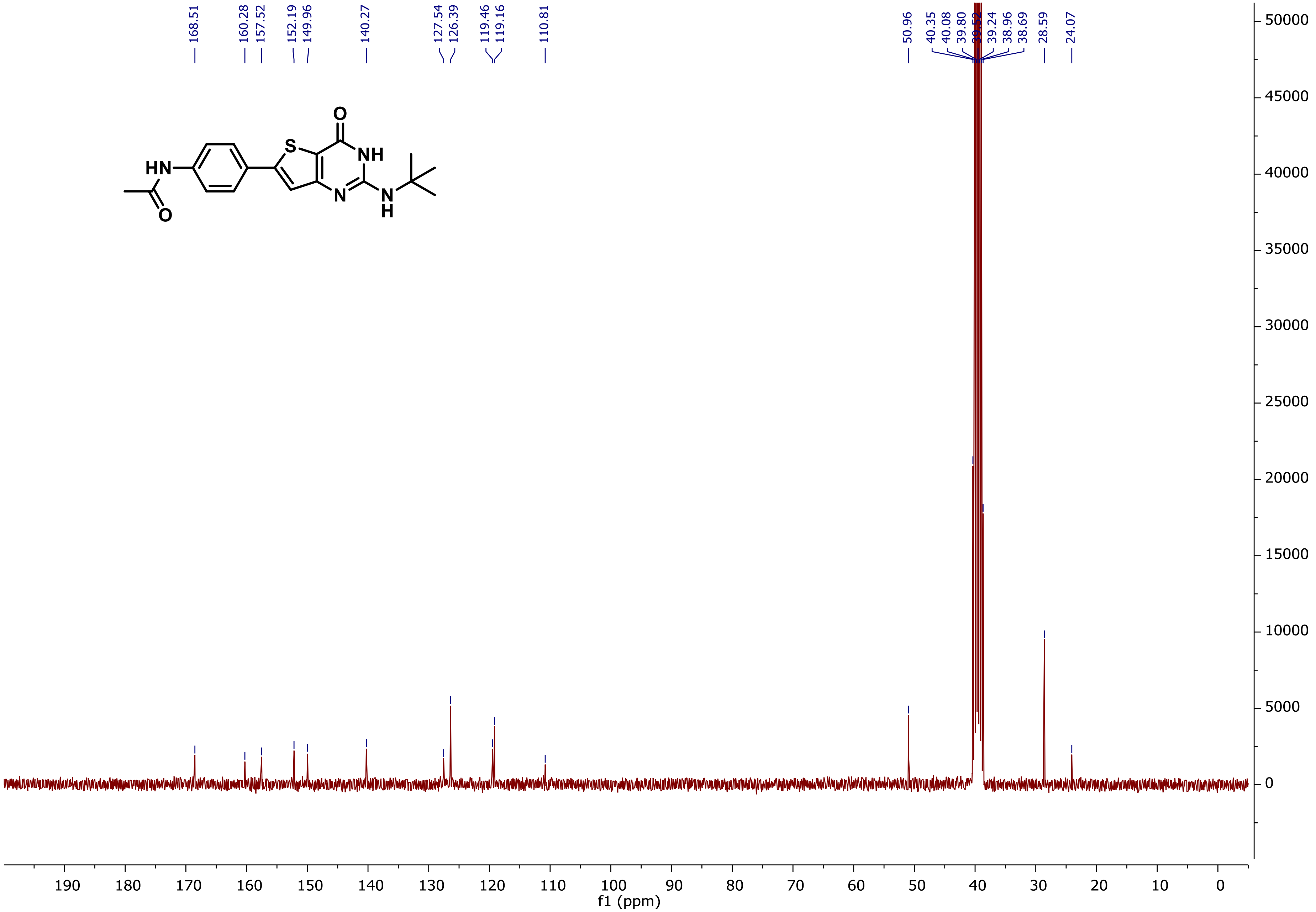
168.51
160.28
157.52
152.19
149.96
140.27
127.54
126.39
119.46
119.16
110.81
50.96
40.35
40.08
39.80
39.52
39.24
38.96
38.69
28.59
24.07
HN
O
S
NH
N
H
50000
45000
40000
35000
30000
25000
20000
15000
10000
5000
0
190
180
170
160
150
140
130
120
110
100
90
80
70
60
50
40
30
20
10
0
f1 (ppm)

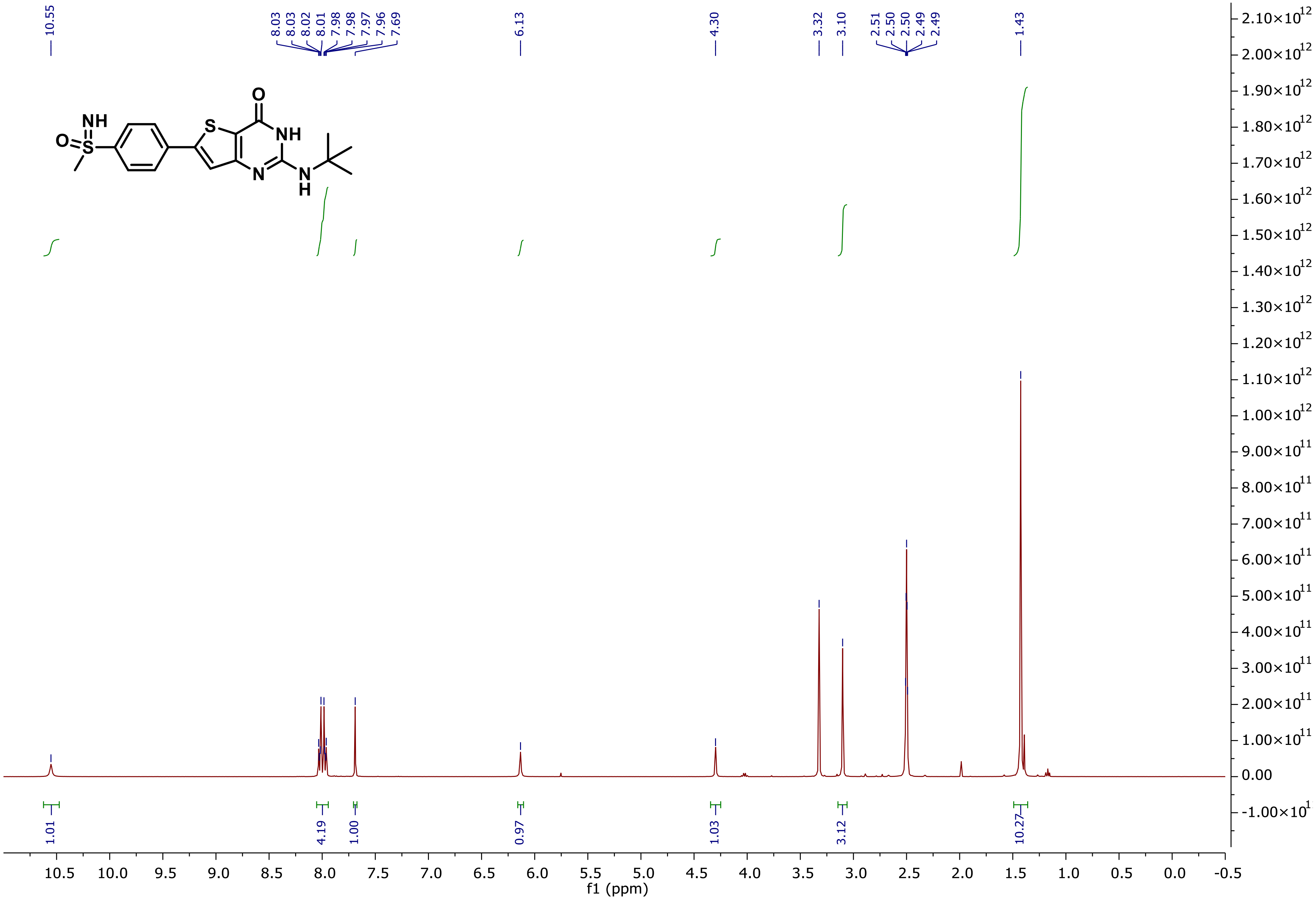
10.55
8.03
8.03
8.02
8.01
7.98
7.98
7.97
7.96
7.69
6.13
4.30
3.32
3.10
2.51
2.50
2.50
2.49
2.49
1.43
O
NH
O
S
S
NH
N
N
H
1.01
4.19
1.00
0.97
1.03
3.12
10.27
f1 (ppm)

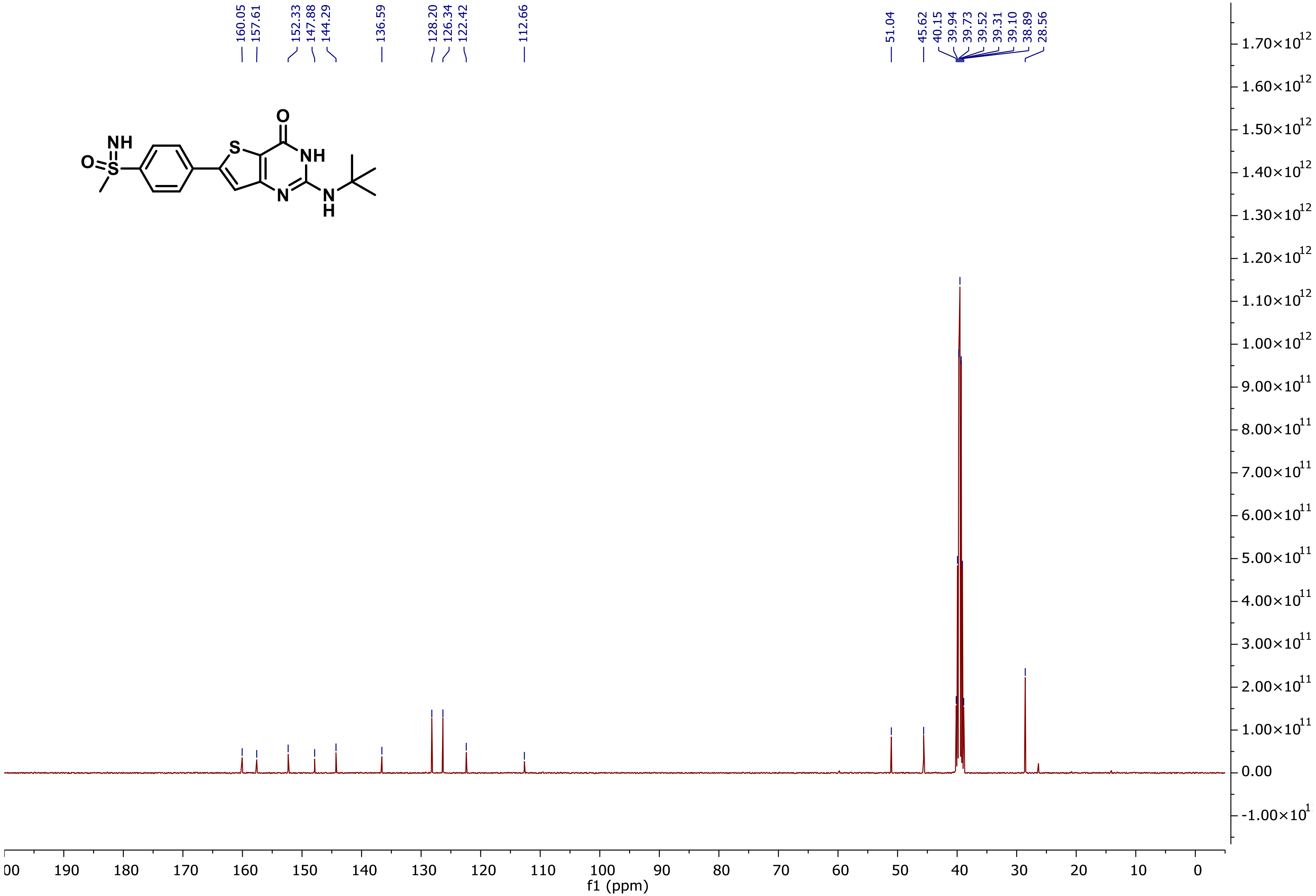
160.05
157.61
152.33
147.88
144.29
136.59
128.20
126.34
122.42
112.66
51.04
45.62
40.15
39.94
39.73
39.52
39.31
39.10
38.89
28.56
NH
O
S
NH
N
H
f1 (ppm)
